# Supplementary material for: Proteomic characterization of epithelial ovarian cancer delineates molecular signatures and therapeutic targets in distinct histological subtypes
Source: Nat Commun. 2023 Nov 28;14:7802. doi: 10.1038/s41467-023-43282-3 (PMC10684593; doi:10.1038/s41467-023-43282-3)
Supplement: Supplementary file 1 — Supplementary Information [file 41467_2023_43282_MOESM1_ESM.pdf]

Supplementary Information

Proteomic characterization of epithelial ovarian cancer delineates molecular signatures and therapeutic targets in distinct histological subtypes

Ting-Ting Gong [1,#], Shuang Guo [2,#], Fang-Hua Liu [3,4,5], Yun-Long Huo [6], Meng Zhang [3,4,5], Shi Yan [3,4,5], Han-Xiao Zhou [2], Xu Pan [2], Xin-Yue Wang [2], He-Li Xu [3,4,5], Ye Kang [6], Yi-Zi Li [3,4,5], Xue Qin [1], Qian Xiao [1,3], Dong-Hui Huang [3,4,5], Xiao-Ying Li [3,4,5], Yue-Yang Zhao [3], Xin-Xin Zhao [3], Ya-Li Wang [3], Xiao-Xin Ma [1], Song Gao [1], Yu-Hong Zhao [3,4,5], Shang-Wei Ning [2,*], Qi-Jun Wu [1,3,4,5,7,*]

[1]Department of Obstetrics and Gynecology, Shengjing Hospital of China Medical University, Shenyang, China.

[2]College of Bioinformatics Science and Technology, Harbin Medical University, Harbin, China.

[3]Department of Clinical Epidemiology, Shengjing Hospital of China Medical University, Shenyang, China.

[4]Clinical Research Center, Shengjing Hospital of China Medical University, Shenyang, China.

[5]Key Laboratory of Precision Medical Research on Major Chronic Disease, Shengjing Hospital of China Medical University, Shenyang, China.

[6]Department of Pathology, Shengjing Hospital of China Medical University, Shenyang, China.

[7]NHC Key Laboratory of Advanced Reproductive Medicine and Fertility (China Medical University), National Health Commission, Shenyang, China.

[#]These authors contributed equally to this work.

*Correspondence: ningsw@ems.hrbmu.edu.cn (S.N.), wuqj@sj-hospital.org (Q.W.).

Supplementary Figures

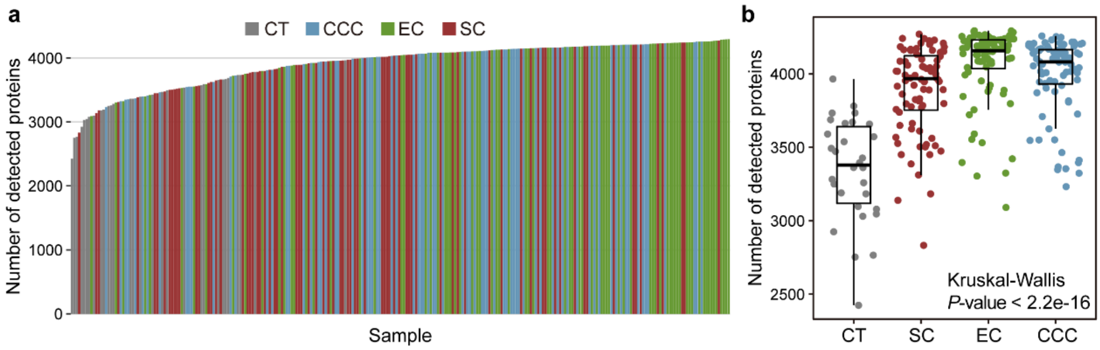

Supplementary Figure 1. Detected proteins in each sample. a Bar plot showing the number of detected proteins in each sample, colored by the epithelial ovarian cancer (EOC) subtypes. **b** Box plot showing the number of detected proteins across EOC subtypes. Clear cell carcinoma (CCC) samples (n = 80), endometrioid carcinoma (EC) samples (n = 79), serous carcinoma (SC) samples (n = 80), and control tissue (CT) samples (n = 30). Boxplots show median (central line), upper and lower quartiles (box limits), min to max range. The *P*-values are calculated using Kruskal-Wallis test.

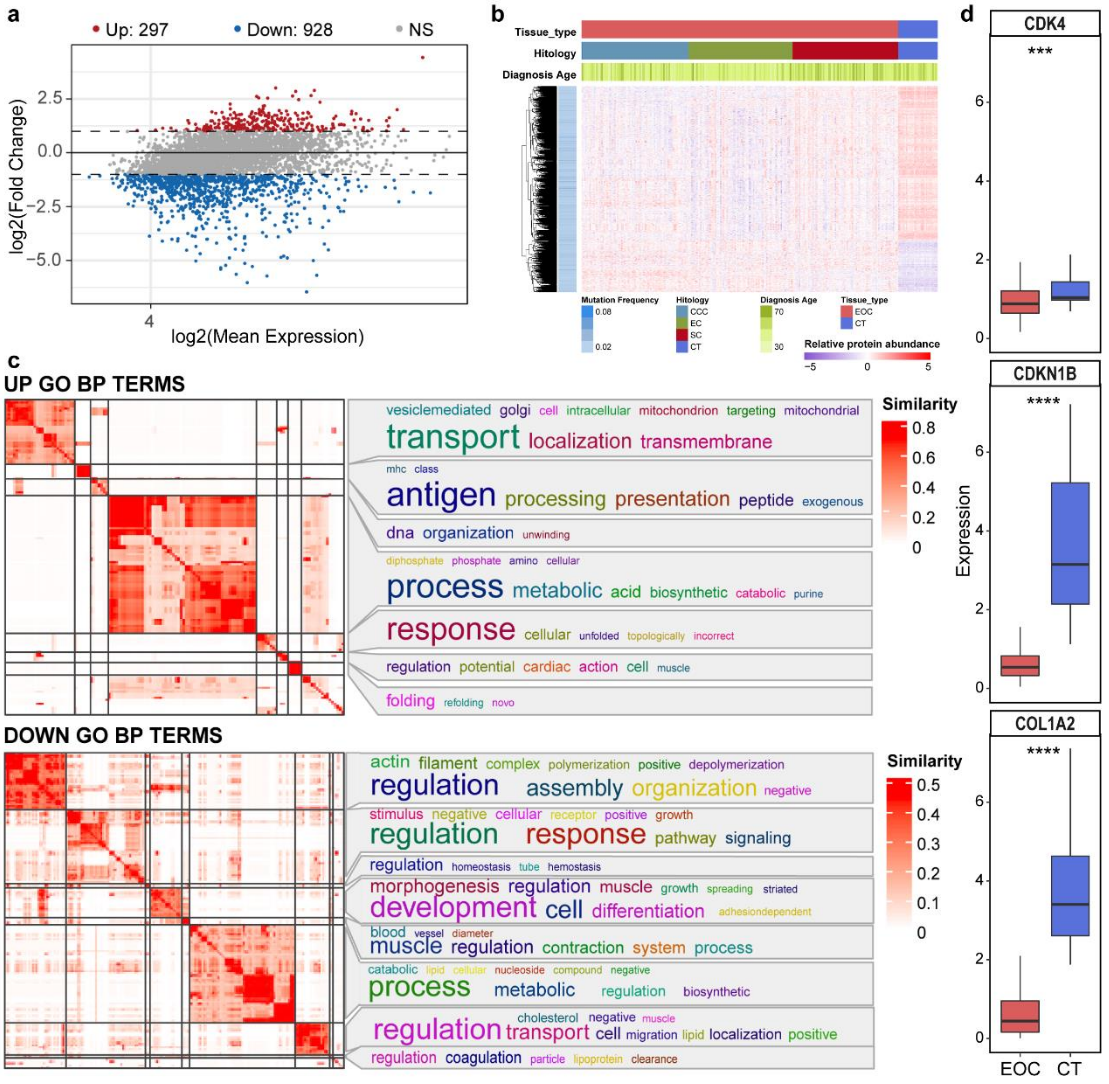

Supplementary Figure 2. Dysregulated proteins and biological processes in EOC. **a** MA plot shows the fold change and mean expression level of all proteins. **b** Heatmap of dysregulated proteins with mutation frequency in the TCGA mc3 project. CCC samples (n = 80), EC samples (n = 79), SC samples (n = 80), and CT samples (n = 30). **c** Similarity matrix for Gene Ontology-biological processes (GO-BP) terms based on the binary-cut method by R package "simplifyEnrichment". **d** The expression levels of these proteins, including CDK4 (*P*-value = 9.5e-04), CDKN1B (*P*-value < 2e-16) and COL4A2 (*P*-value < 2e-16), in EOC samples (n = 239) were significantly lower than in CT samples (n = 30), which were verified in the Parallel Reaction Monitoring (PRM) analysis. The *P*-values are calculated using two-sided Wilcoxon test. The asterisk character represents the significance of the expression discrepancy, ****P*-value < 0.001 and *****P*-value < 0.0001. Boxplots show median (central line), upper and lower quartiles (box limits), min to max range.

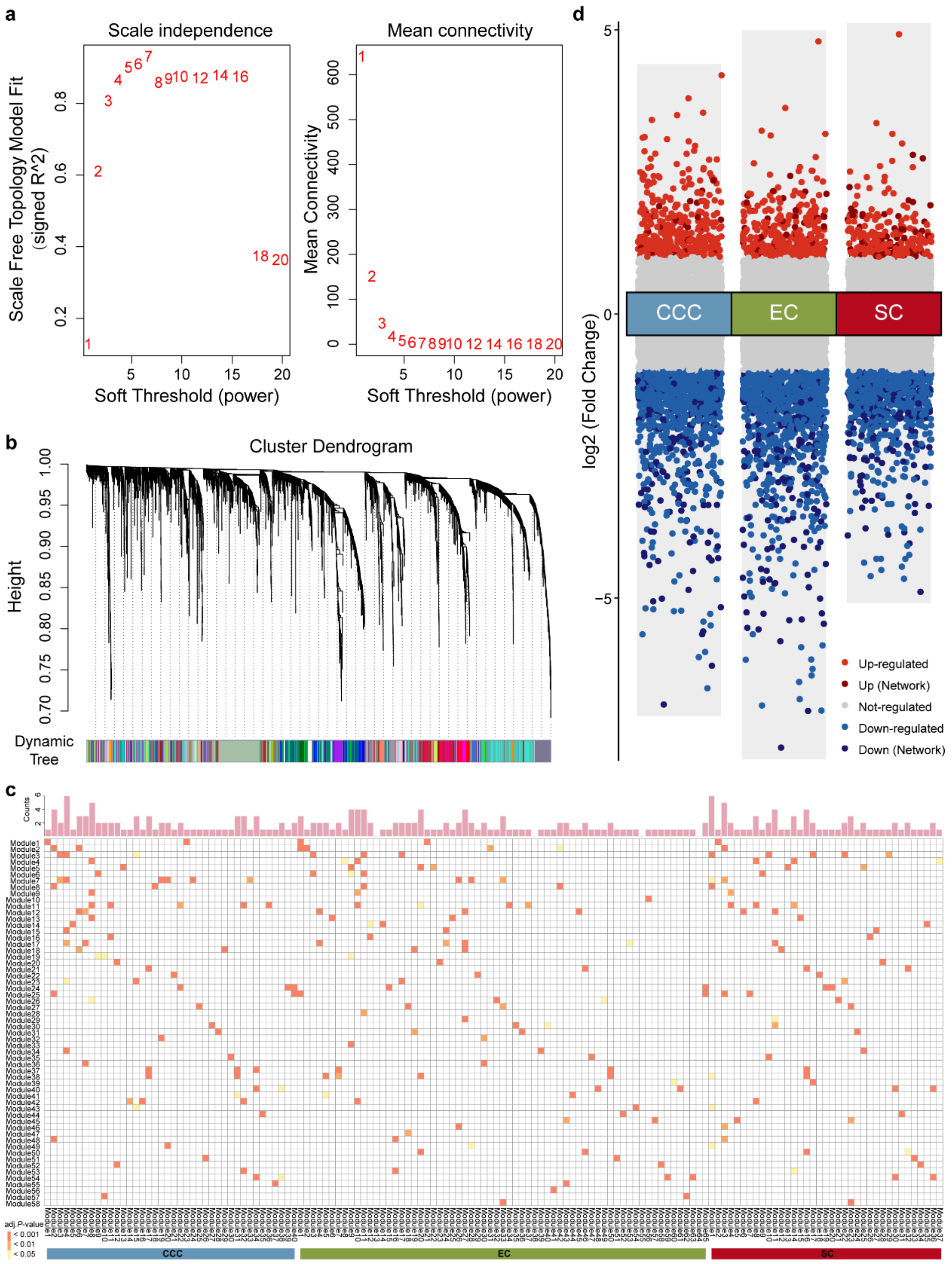

Supplementary Figure 3. Construction of protein co-expression network. a Analysis of the scale-free fit index and the mean connectivity for the determination of soft-thresholding powers. **b** Hierarchical clustering dendrogram of proteins in different EOC modules. **c** Degree of overlap between proteins in the EOC modules and the histological subtype modules (hypergeometric test and BH adjusted). **d** Aberrant protein expression in three histological subtypes.

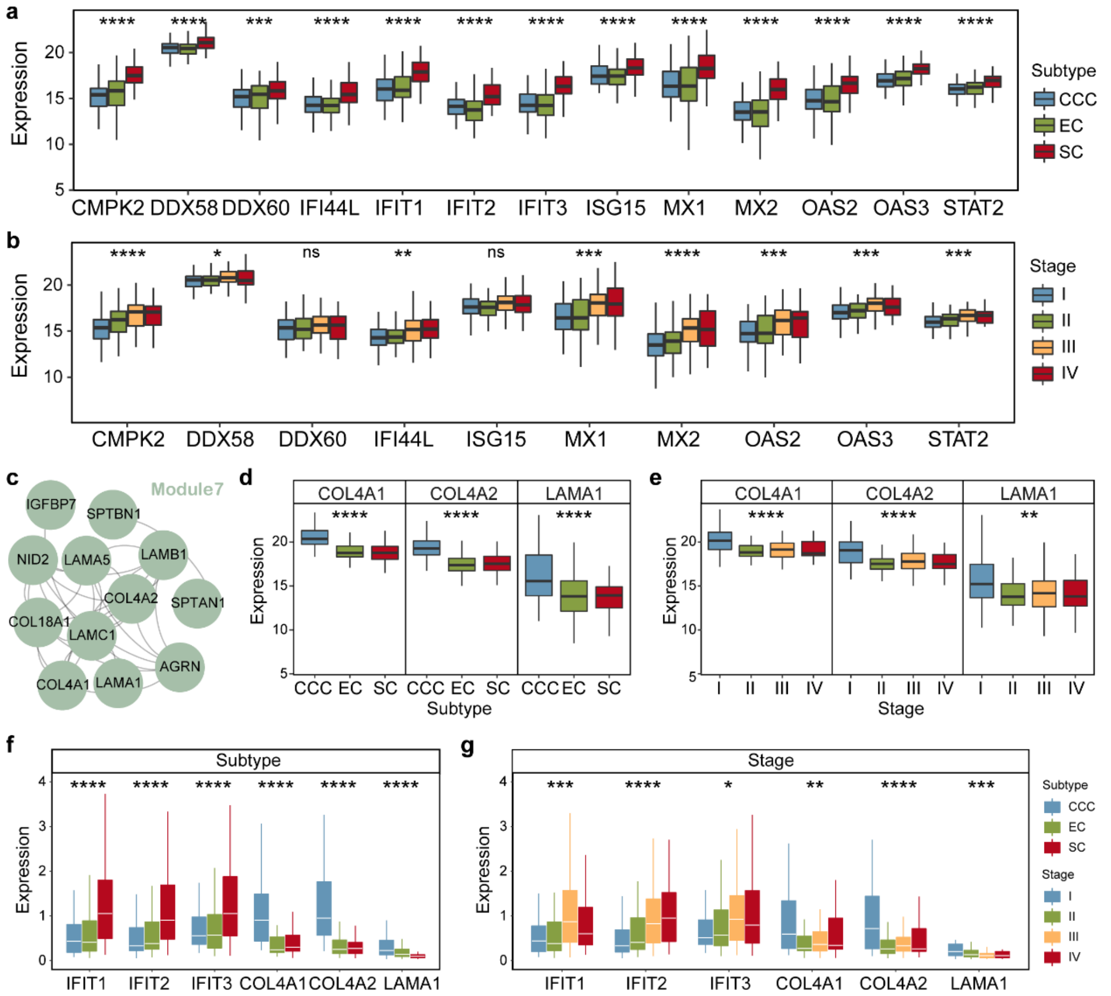

Supplementary Figure 4. Protein expression of core modules in EOC. a-b Boxplots illustrating the abundance of Module31 proteins in different histological subtypes and stages. **c** Sub-network of Module7. **d-e** Boxplots illustrated the abundance of COL4A1, COL4A2, and LAMA1 in the different histological subtypes and stages. **f-g** In the PRM analysis, the expression characteristics of these proteins, including IFIT1, IFIT2, IFIT3, COL4A1, COL4A2, and LAMA1, in histological subtypes and stages were verified. CCC samples (n = 80), EC samples (n = 79), and SC samples (n = 80). Stage I samples (n = 81), Stage II samples (n = 47), Stage III samples (n = 88), and Stage IV samples (n = 23). The *P*-values are calculated using Kruskal-Wallis test. The asterisk character represents the significance of the expression discrepancy, **P*-value < 0.05; ***P*-value < 0.01; ****P*-value < 0.001; *****P*-value < 0.0001. And, ns represents not significant. Boxplots show median (central line), upper and lower quartiles (box limits), min to max range.

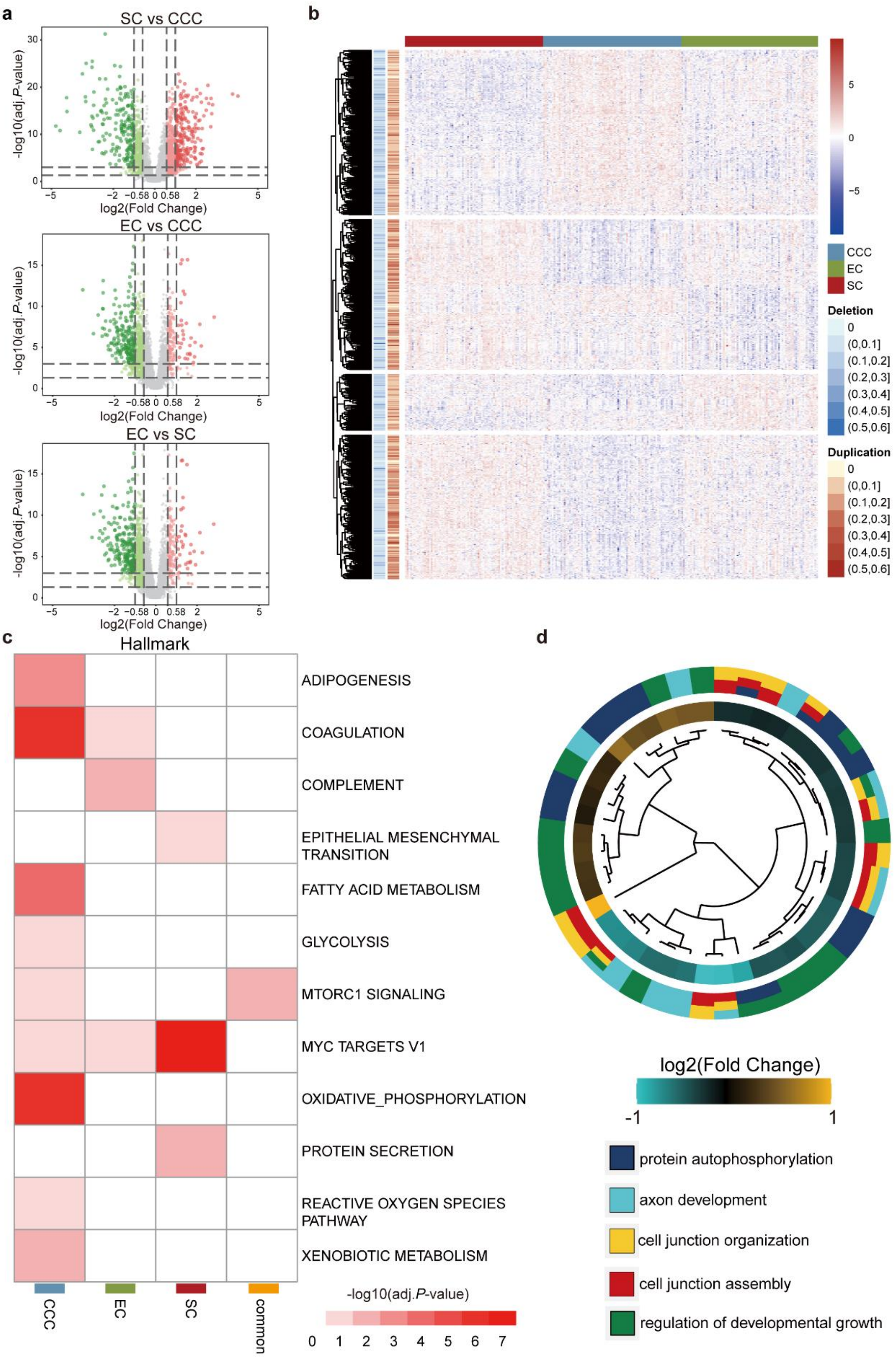

Supplementary Figure 5. Abnormally expressed proteins and their functional characteristics in distinct histological subtypes. a Volcano plot indicating proteins up-regulated and down-regulated in different subtypes. CCC samples (n = 80), EC

samples (n = 79), and SC samples (n = 80). *P*-values were calculated using the K-W test. Light red and green colors represent proteins with *P*-values < 0.05, whereas red and green represent proteins with *P*-values < 0.05 and $|\log2 \text{ (Fold Change)}| > 0.58$. Other proteins are colored in gray. **b** Heatmap representation of the relative protein abundance of common differentially expressed proteins among the three subtypes. CCC samples (n = 80), EC samples (n = 79), and SC samples (n = 80). The left panel shows the TCGA copy number variation frequency of these proteins. **c** Functional enrichment analysis revealed the hallmarks that were significantly enriched in the EOC subtypes and common proteins. CCC-specific proteins (n = 861), EC-specific proteins (n = 423), SC-specific proteins (n = 1094), and common proteins (n = 673). The adj.*P*-values are calculated using hypergeometric test (BH adjusted *P*-values). **d** Hierarchical clustering of the protein expression profiles in each GO term (common proteins (n = 673)).

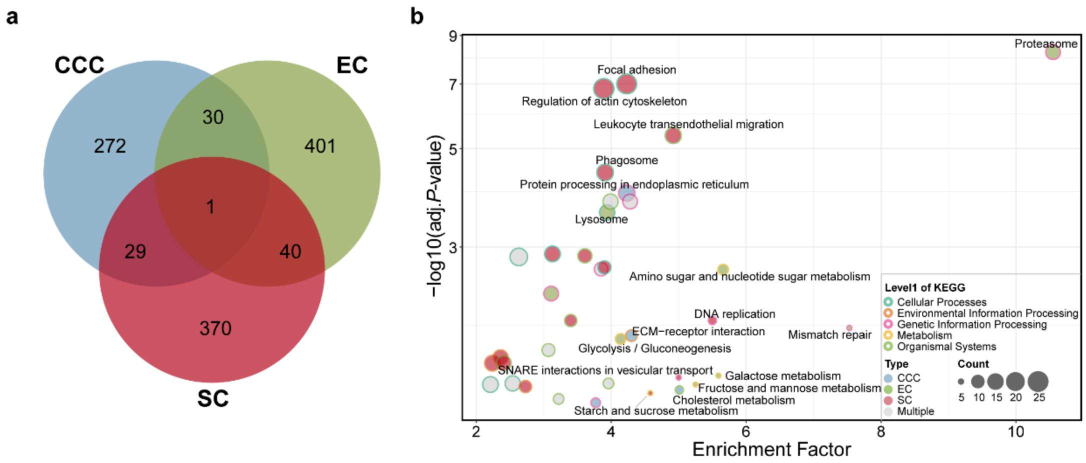

Supplementary Figure 6. Comparison and characterization of tumor progression-related proteins in three histological subtypes. a Venn diagram shows the overlap of differential tumor progression landmarks reported in different subtypes of EOC. CCC samples (n = 80), EC samples (n = 79), and SC samples (n = 80). **b** KEGG signaling pathways enriched for tumor progression landmarks in the three histological subtypes, respectively. The numbers of tumor progression markers for CCC, EC and SC subtypes were 332, 472 and 440, respectively. The adj.*P*-values are calculated using hypergeometric test (BH adjusted *P*-values).

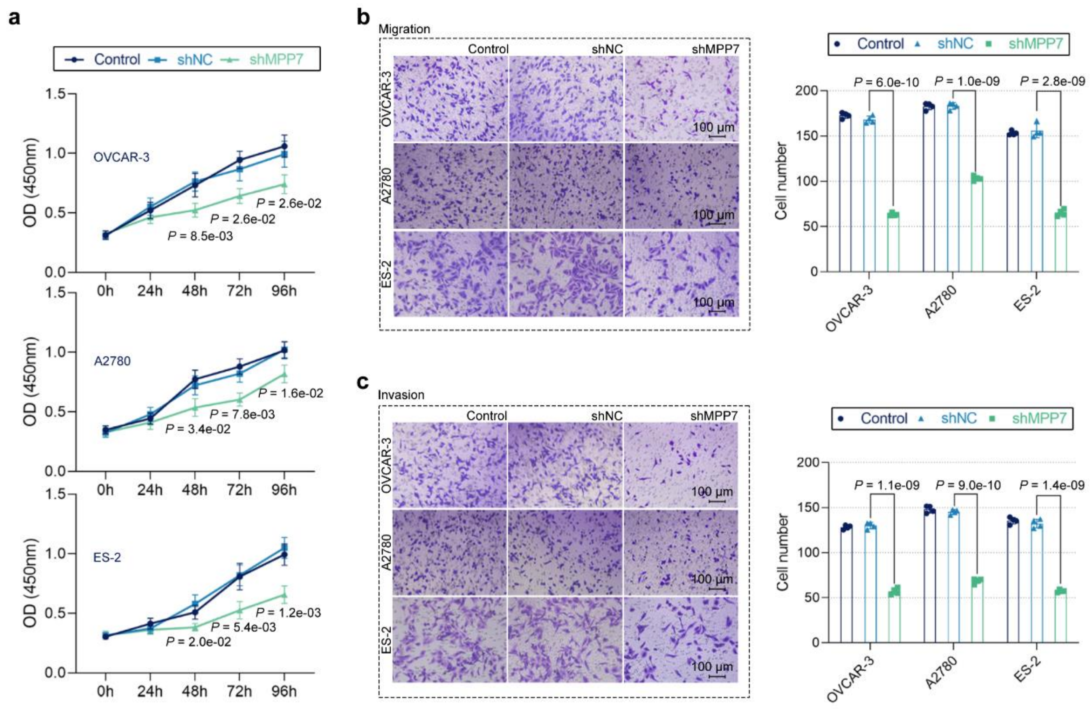

Supplementary Figure 7. Effect of MPP7 knockdown on the malignant behavior of epithelial ovarian cancer cells. OVCAR-3, A2780, and ES-2 cells were infected with lentiviral vectors carrying shMPP7 or shNC. **a** CCK-8 assays were performed at 0, 24, 48, 72, and 96 h after infection. *P*-values compared with the shNC group. All experiments were repeated four times. **b-c** Cell migration (b) and invasion (c) were determined by transwell assays at 48 h after infection. Scale bar: 100 μm. Data are presented by mean ± standard deviation and analyzed by the one-way or two-way analysis of variance (ANOVA) followed by Tukey's tests. All experiments were repeated four times.

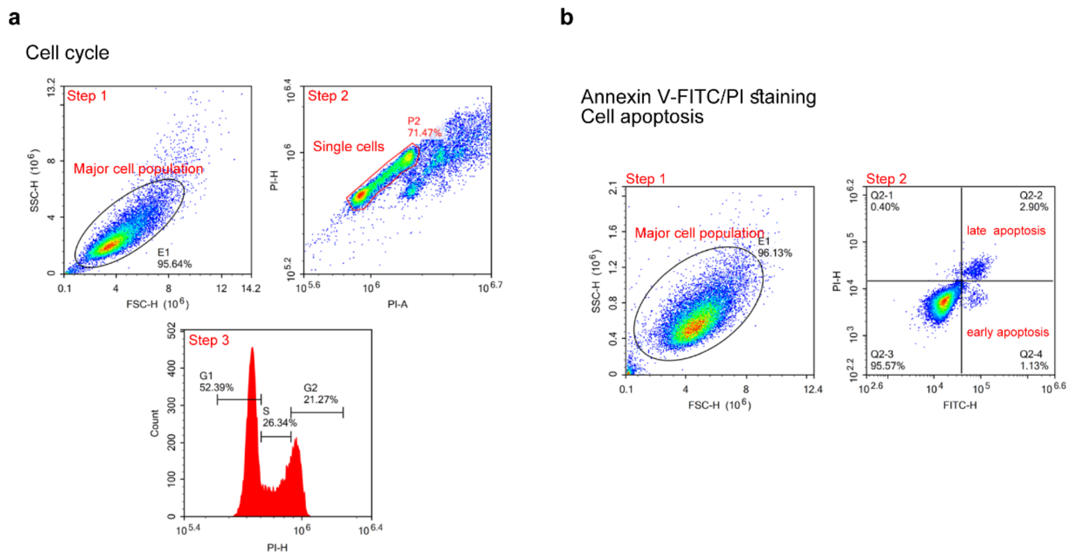

Supplementary Figure 8. Gating strategies for flow cytometric analysis. a Gating strategies for cell cycle analysis. The major cell population was selected based on FSC and SSC, and then the single cells were gated by PI-A and PI-H. The signal of PI in the single-cell population was analyzed. **b** Gating strategies for cell apoptosis analysis (Annexin V-FITC-PI staining). Cells were examined by FSC and SSC to obtain the major cell population. Then, the signal of FITC and PI in the cell population was analyzed.
