# Supplementary material for: Proteomic characterization of epithelial ovarian cancer delineates molecular signatures and therapeutic targets in distinct histological subtypes
Source: Nat Commun. 2023 Nov 28;14:7802. doi: 10.1038/s41467-023-43282-3 (PMC10684593; doi:10.1038/s41467-023-43282-3)
Supplement: Supplementary file 3 — Description of Additional Supplementary Files [file 41467_2023_43282_MOESM3_ESM.pdf]

Description of Additional Supplementary Files

Supplementary Data 1. Clinicopathological characteristics of EOC patients.

Supplementary Data 2. Summary of clinical information for CCC, EC, and SC patients.

Supplementary Data 3. Dysregulated proteins in EOC.

Supplementary Data 4. Biological processes or signaling pathways enriched for up- and down-regulated proteins, respectively.

Supplementary Data 5. GO biological processes and KEGG pathways with GSEA enrichment.

Supplementary Data 6. Prognostic value of key proteins in disordered signaling pathways.

Supplementary Data 7. Dysregulated proteins in CCC, EC and SC histological subtypes.

Supplementary Data 8. KEGG signaling pathways enriched for tumor progression landmarks of each histological subtype.

Supplementary Data 9. Raw data of CCK-8 assays.

Supplementary Data 10. Raw data of flow cytometric analysis of cell cycle.

Supplementary Data 11. Raw data of flow cytometric analysis of cell apoptosis.

Supplementary Data 12. Raw data of transwell assay (cell migration).

Supplementary Data 13. Raw data of matrigel-coated transwell assay (cell invasion).

Supplementary Data 14. Materials and reagents required for sample preparation.
