# Supplementary material for: Proteomic characterization of epithelial ovarian cancer delineates molecular signatures and therapeutic targets in distinct histological subtypes
Source: Nat Commun. 2023 Nov 28;14:7802. doi: 10.1038/s41467-023-43282-3 (PMC10684593; doi:10.1038/s41467-023-43282-3)
Supplement: Supplementary file 18 — Reporting Summary [file 41467_2023_43282_MOESM18_ESM.pdf]

nature portfolio

Corresponding author(s): Shangwei Ning

Last updated by author(s): Oct 31, 2023

Reporting Summary

Nature Portfolio wishes to improve the reproducibility of the work that we publish. This form provides structure for consistency and transparency in reporting. For further information on Nature Portfolio policies, see our Editorial Policies and the Editorial Policy Checklist.

Statistics

For all statistical analyses, confirm that the following items are present in the figure legend, table legend, main text, or Methods section.

n/a	Confirmed	
☐	☒	The exact sample size (*n*) for each experimental group/condition, given as a discrete number and unit of measurement
☐	☒	A statement on whether measurements were taken from distinct samples or whether the same sample was measured repeatedly
☐	☒	The statistical test(s) used AND whether they are one- or two-sided<br>*Only common tests should be described solely by name; describe more complex techniques in the Methods section.*
☒	☐	A description of all covariates tested
☐	☒	A description of any assumptions or corrections, such as tests of normality and adjustment for multiple comparisons
☐	☒	A full description of the statistical parameters including central tendency (e.g. means) or other basic estimates (e.g. regression coefficient) AND variation (e.g. standard deviation) or associated estimates of uncertainty (e.g. confidence intervals)
☐	☒	For null hypothesis testing, the test statistic (e.g. *F*, *t*, *r*) with confidence intervals, effect sizes, degrees of freedom and *P* value noted<br>*Give P values as exact values whenever suitable.*
☒	☐	For Bayesian analysis, information on the choice of priors and Markov chain Monte Carlo settings
☒	☐	For hierarchical and complex designs, identification of the appropriate level for tests and full reporting of outcomes
☐	☒	Estimates of effect sizes (e.g. Cohen's *d*, Pearson's *r*), indicating how they were calculated

Our web collection on statistics for biologists contains articles on many of the points above.

Software and code

Policy information about availability of computer code

Data collection	A total of 239 formalin-fixed, paraffin-embedded (FFPE) ovarian epithelial tissues were acquired from newly diagnosed EOC patients undergoing primary debulking surgery at the Shengjing Hospital of China Medical University (Shenyang, China) from 2013 to 2019. The 239 patients were included in the present analysis with three histological types: SC samples (n = 80), EC samples (n = 79) and CCC samples (n = 80). The histologically normal ovarian tissues (n=30) taken from cases of uterine fibroids were used as CT samples, in which the ovary was surgically removed incidental to radical surgery. The proteome analysis was performed using label-free technology on the same mass spectrometer with consistent quality control.
Data analysis	The LFQ intensity of the proteins was normalized using the normalized quantile functions in the R package 'limma'. Missing values were imputed using the DreamAI algorithm. Statistical analysis, included Fisher's exact test, Wilcoxon test, Kruskal-Wallis test, Post-hoc tests, one-way or two-way analysis of ANOVA, Benjamini-Hochberg (BH) correction, were realized by R (v3.6.1). Survival analysis, included Cox regression analysis, Kaplan–Meier curve and log-rank test, were realized by R (v3.6.1). Functional enrichment analysis was performed in R (v3.6.1), using packages: clusterProfiler, fgsea, simplifyEnrichment and GOSemSim. Protein network was performed in R (v3.6.1) package WGCNA and Cytoscape (v3.6.0). All code for computational analyses were derived from publicly available websites and previous publications, and are cited in the corresponding Methods sections.

For manuscripts utilizing custom algorithms or software that are central to the research but not yet described in published literature, software must be made available to editors and reviewers. We strongly encourage code deposition in a community repository (e.g. GitHub). See the Nature Portfolio guidelines for submitting code & software for further information.

Data

Policy information about availability of data

All manuscripts must include a data availability statement. This statement should provide the following information, where applicable:

- Accession codes, unique identifiers, or web links for publicly available datasets
- A description of any restrictions on data availability
- For clinical datasets or third party data, please ensure that the statement adheres to our policy

All relevant data supporting the key findings of this study are either downloaded from open repositories or have been uploaded to such repositories and are publicly available. The mass spectrometry proteomic data generated in this study have been deposited in the ProteomeXchange Consortium via the PRIDE partner repository under accession code PXD033741 [https://www.ebi.ac.uk/pride/archive/projects/PXD033741]. The mass spectrometry proteomic data generated in this study have been deposited in the OMIX under accession code OMIX002719 [https://ngdc.cncb.ac.cn/omix/release/OMIX002719]. The exosome protein lists used in this study are available in the ExoCarta (http://www.exocarta.org/) and Vesiclepedia (http://microvesicles.org/) databases. The protein-protein interactions used in this study are available in the STRING (https://cn.string-db.org/) database. The approved drug-target protein lists used in this study are available in the Drug-Gene Interaction database (DGIdb, https://dgidb.genome.wustl.edu/). The remaining data are available within the Article, Supplementary Information or Source Data file. Source data are provided with this paper.

Human research participants

Policy information about studies involving human research participants and Sex and Gender in Research.

Reporting on sex and gender	The study population was epithelial ovarian cancer patients, therefore all sexes were female.
Population characteristics	Patient data is available in Supplementary Data 1. Histopathology data was obtained from the respective pathology reports.
Recruitment	Samples were acquired from newly diagnosed epithelial ovarian cancer patients undergoing primary debulking surgery at the Shengjing Hospital of China Medical University (Shenyang, China) from 2013 to 2019.
Ethics oversight	The present study was approved by the institutional Review Board of Shenging Hospital of China Medical University (2020P5265K).

Note that full information on the approval of the study protocol must also be provided in the manuscript.

Field-specific reporting

Please select the one below that is the best fit for your research. If you are not sure, read the appropriate sections before making your selection.

[x] Life sciences [ ] Behavioural & social sciences [ ] Ecological, evolutionary & environmental sciences

For a reference copy of the document with all sections, see nature.com/documents/nr-reporting-summary-flat.pdf

Life sciences study design

All studies must disclose on these points even when the disclosure is negative.

Sample size	269 samples (239 epithelial ovarian cancer samples and 30 control tissue samples).
Data exclusions	No data exclusions.
Replication	Reproducible.
Randomization	Not applicable for this study.
Blinding	Not applicable for this study.

Reporting for specific materials, systems and methods

We require information from authors about some types of materials, experimental systems and methods used in many studies. Here, indicate whether each material, system or method listed is relevant to your study. If you are not sure if a list item applies to your research, read the appropriate section before selecting a response.

Materials & experimental systems

n/a	Involved in the study	
☒	☐	Antibodies
☐	☒	Eukaryotic cell lines
☒	☐	Palaeontology and archaeology
☒	☐	Animals and other organisms
☒	☐	Clinical data
☒	☐	Dual use research of concern

Methods

n/a	Involved in the study	
☒	☐	ChIP-seq
☐	☒	Flow cytometry
☒	☐	MRI-based neuroimaging

Eukaryotic cell lines

Policy information about cell lines and Sex and Gender in Research

Cell line source(s)	Ovarian cancer cell lines OVCAR-3, A2780, and ES-2 were purchased from icellbioscience (China).
Authentication	All human cancer cell lines were authenticated by STR profiling.
Mycoplasma contamination	Mycoplasma tests were performed. All human cancer cell lines were mycoplasma-negative.
Commonly misidentified lines (See ICLAC register)	None

Flow Cytometry

Plots

Confirm that:

☒ The axis labels state the marker and fluorochrome used (e.g. CD4-FITC).

☒ The axis scales are clearly visible. Include numbers along axes only for bottom left plot of group (a 'group' is an analysis of identical markers).

☒ All plots are contour plots with outliers or pseudocolor plots.

☒ A numerical value for number of cells or percentage (with statistics) is provided.

Methodology

Sample preparation	For cell cycle analysis, cells were fixed with 70% cold ethanol and washed with PBS at 48 h after infection. After incubation with RNase A at 37℃ for 30 min, the cells were stained with Propidium Iodide (PI) for 30 min.<br>For cell apoptosis detection, cells were washed with PBS at 48 h after infection and resuspended in binding buffer. Then, the cells were stained with Annexin V-FITC and PI for 15 min.
Instrument	The signal of PI and Annexin V-FITC was detected by a NovoCyte flow cytometer (Agilent, USA).
Software	The results obtained from flow cytometric analysis were analyzed by the NovoExpress software (version 1.4.1, Agilent, USA).
Cell population abundance	N\A
Gating strategy	The gating strategy was shown in Supplementary Figure 8A (cell cycle; PI) and Supplementary Figure 8B (cell apoptosis; Annexin V-FITC/PI).

☒ Tick this box to confirm that a figure exemplifying the gating strategy is provided in the Supplementary Information.
