# Supplementary figures and images for: Proteomic characterization of epithelial ovarian cancer delineates molecular signatures and therapeutic targets in distinct histological subtypes
Source: Nat Commun. 2023 Nov 28;14:7802. doi: 10.1038/s41467-023-43282-3 (PMC10684593; doi:10.1038/s41467-023-43282-3)

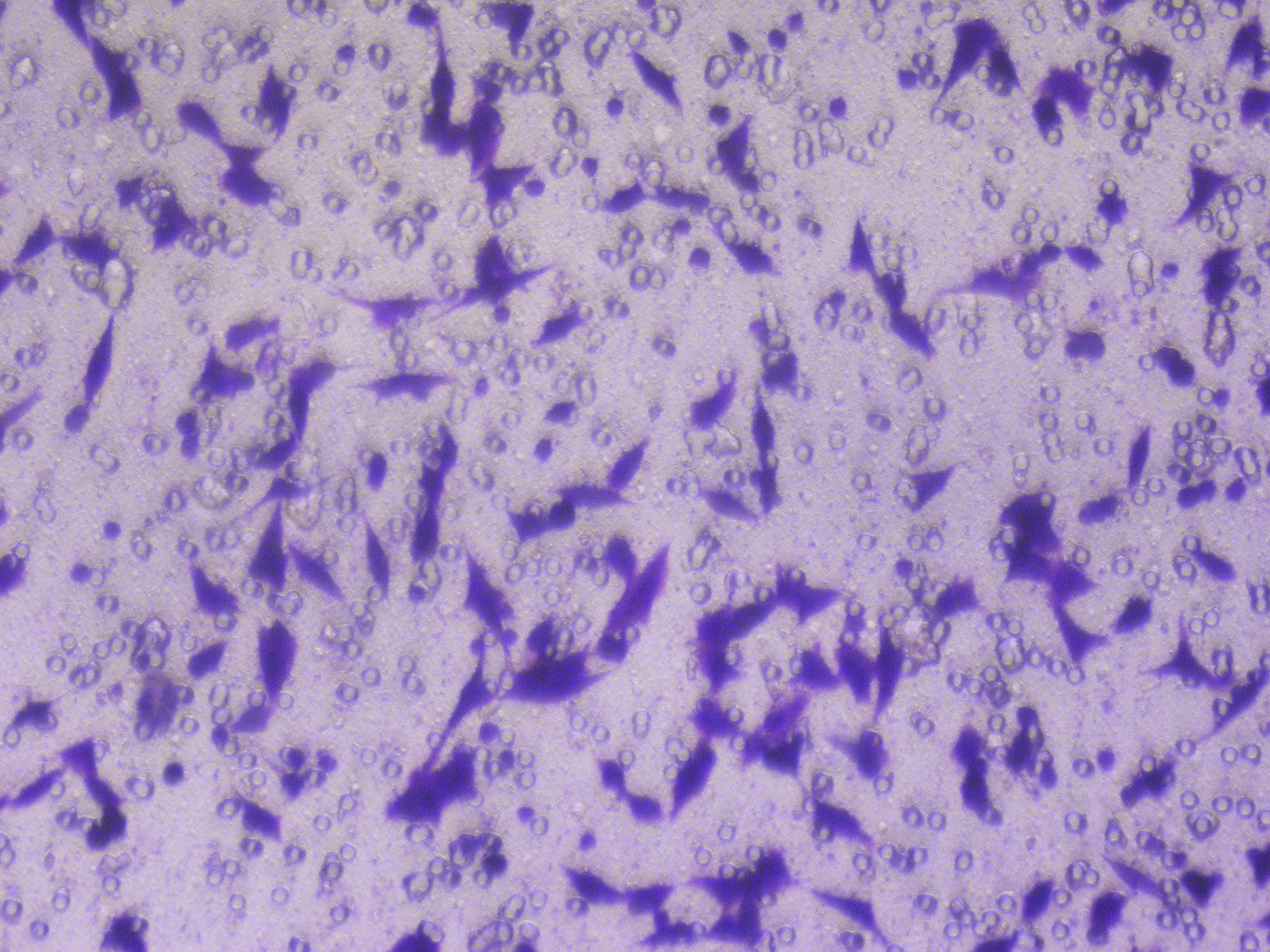

Supplement: Supplementary file 19 — Source Data [file 41467_2023_43282_MOESM19_ESM.zip › Source Data/Source data-Transwell raw images/Invasion/A OVCAR-3/Control-rep1.jpg]

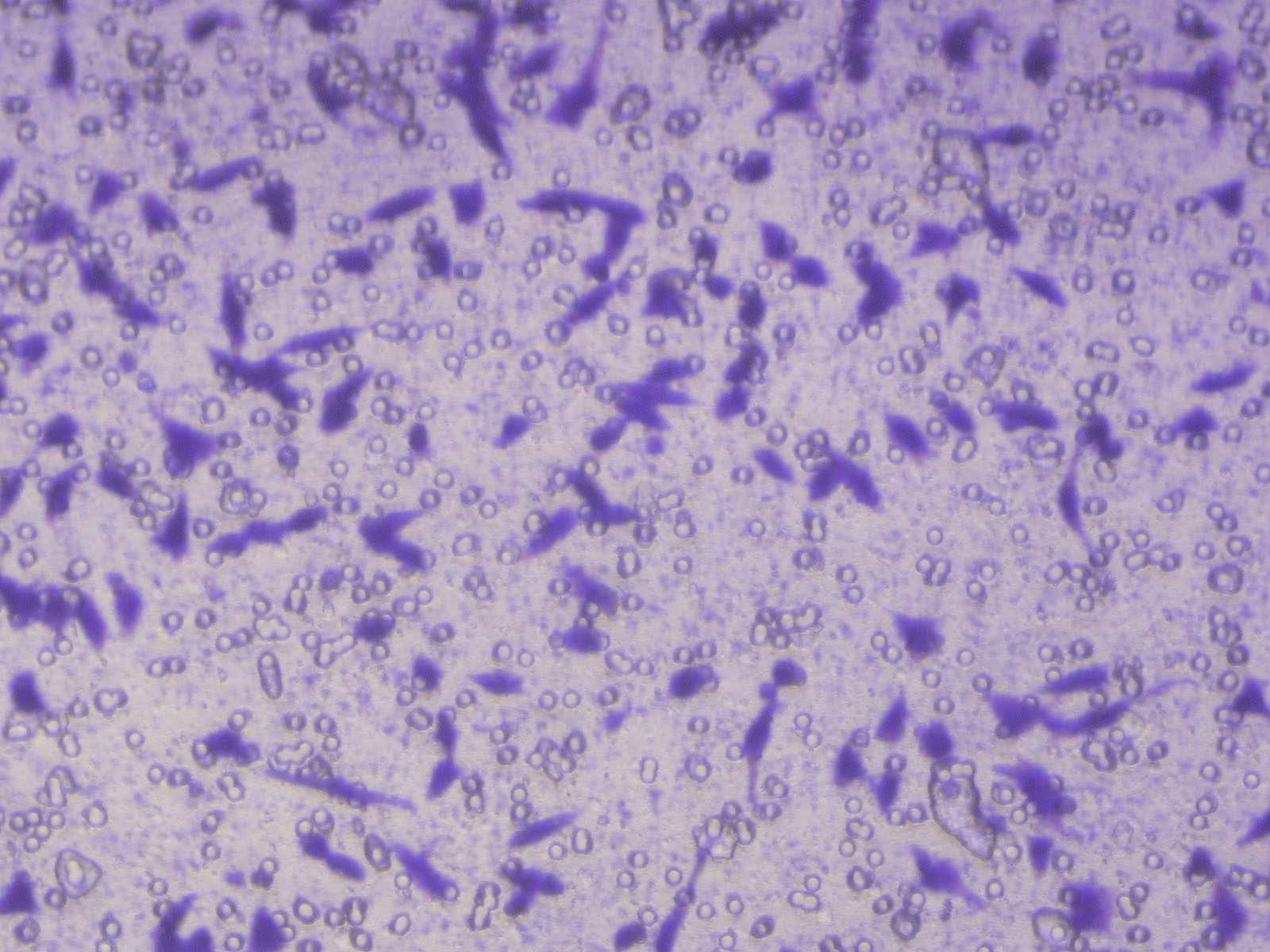

Supplement: Supplementary file 19 — Source Data [file 41467_2023_43282_MOESM19_ESM.zip › Source Data/Source data-Transwell raw images/Invasion/A OVCAR-3/Control-rep2.jpg]

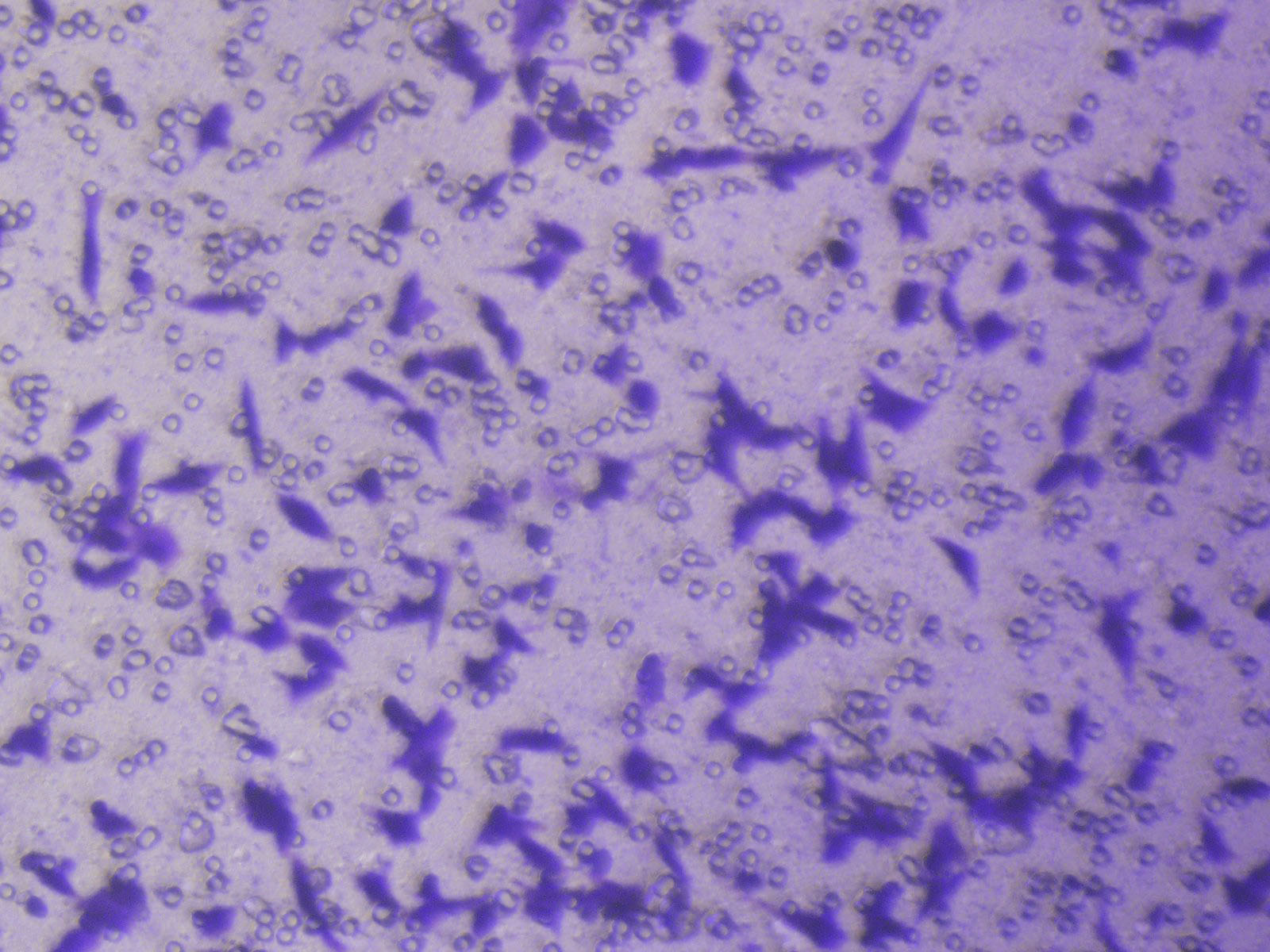

Supplement: Supplementary file 19 — Source Data [file 41467_2023_43282_MOESM19_ESM.zip › Source Data/Source data-Transwell raw images/Invasion/A OVCAR-3/Control-rep3.jpg]

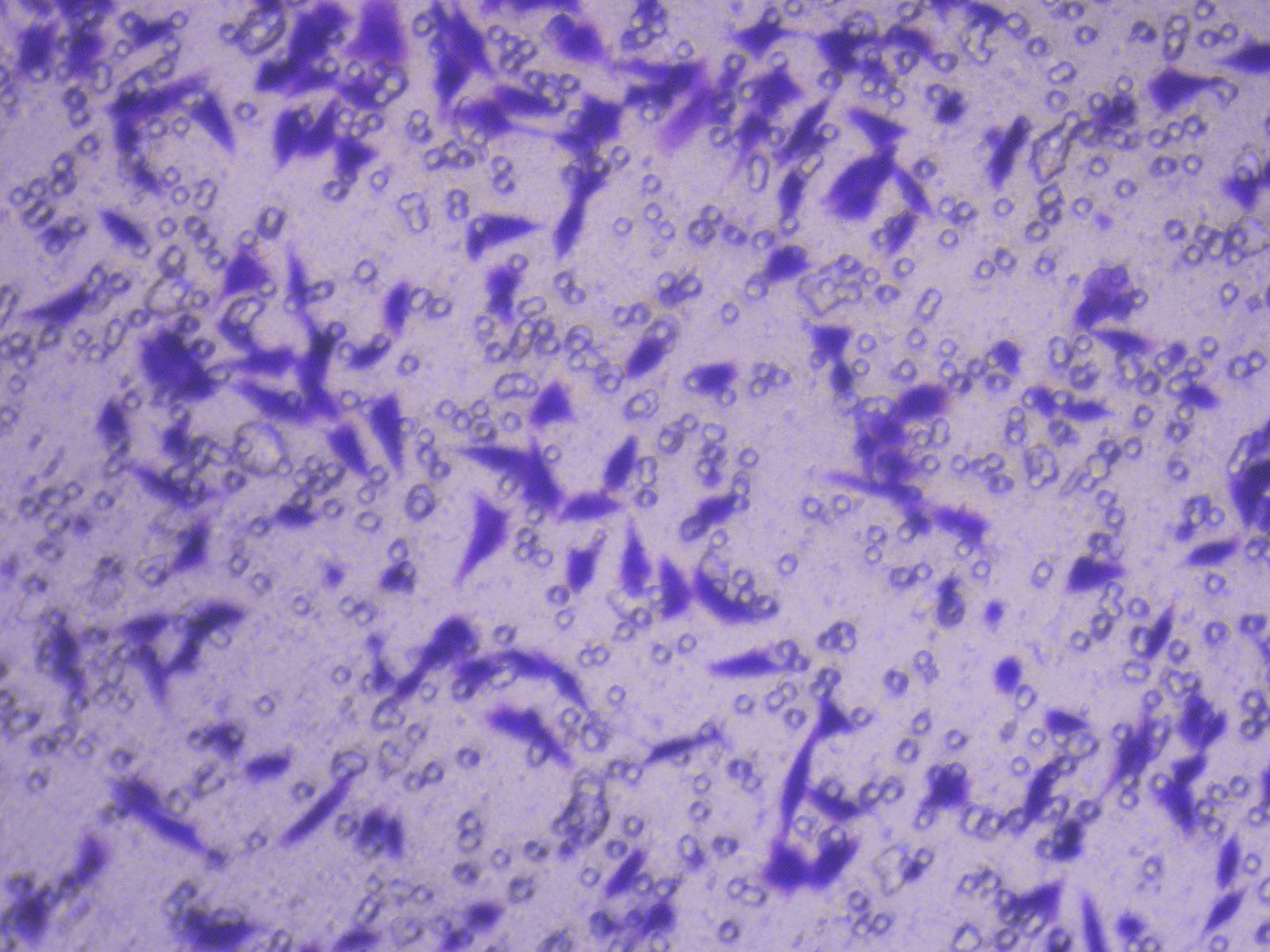

Supplement: Supplementary file 19 — Source Data [file 41467_2023_43282_MOESM19_ESM.zip › Source Data/Source data-Transwell raw images/Invasion/A OVCAR-3/Control-rep4.jpg]

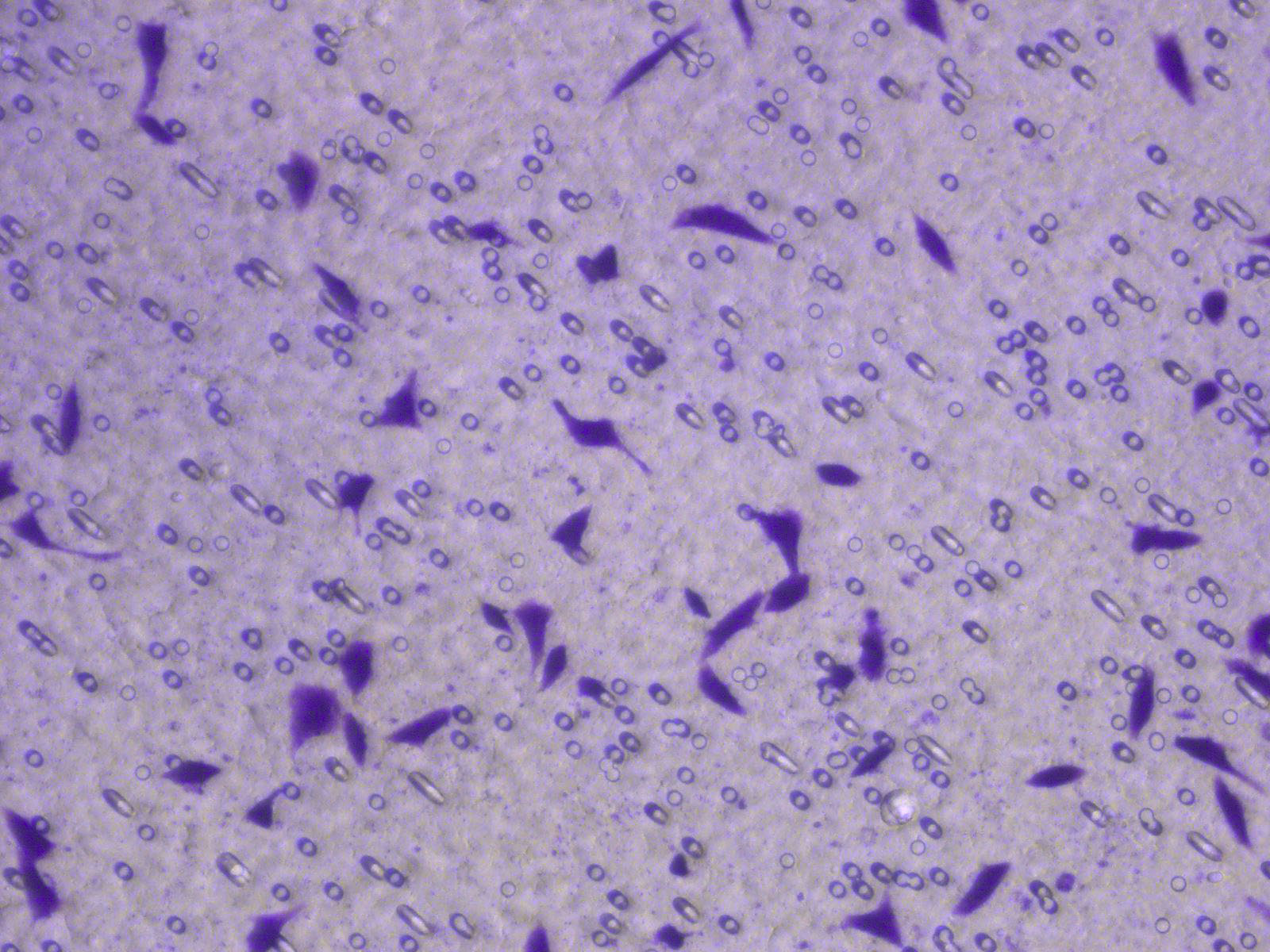

Supplement: Supplementary file 19 — Source Data [file 41467_2023_43282_MOESM19_ESM.zip › Source Data/Source data-Transwell raw images/Invasion/A OVCAR-3/shMPP7-rep1.jpg]

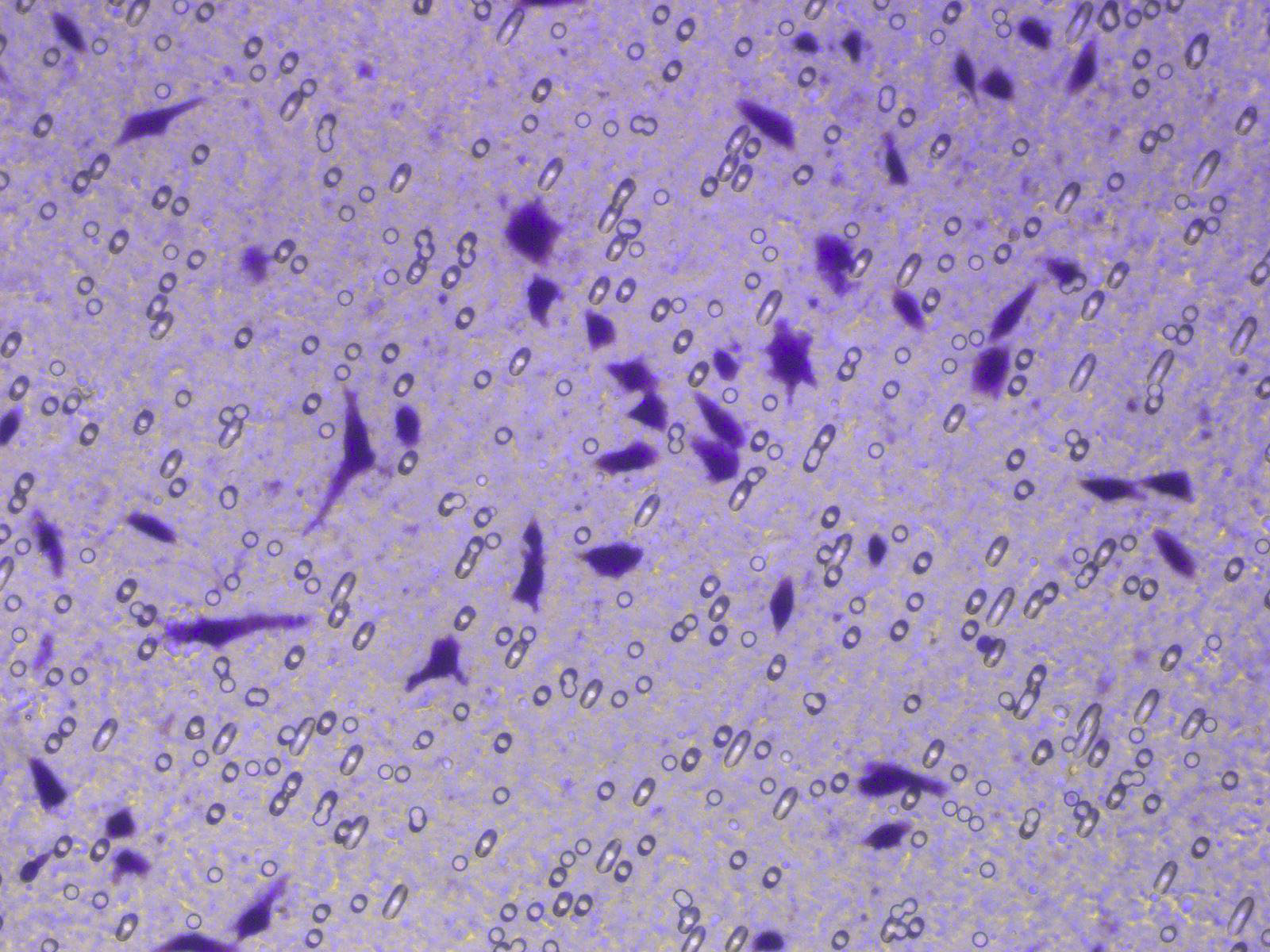

Supplement: Supplementary file 19 — Source Data [file 41467_2023_43282_MOESM19_ESM.zip › Source Data/Source data-Transwell raw images/Invasion/A OVCAR-3/shMPP7-rep2.jpg]

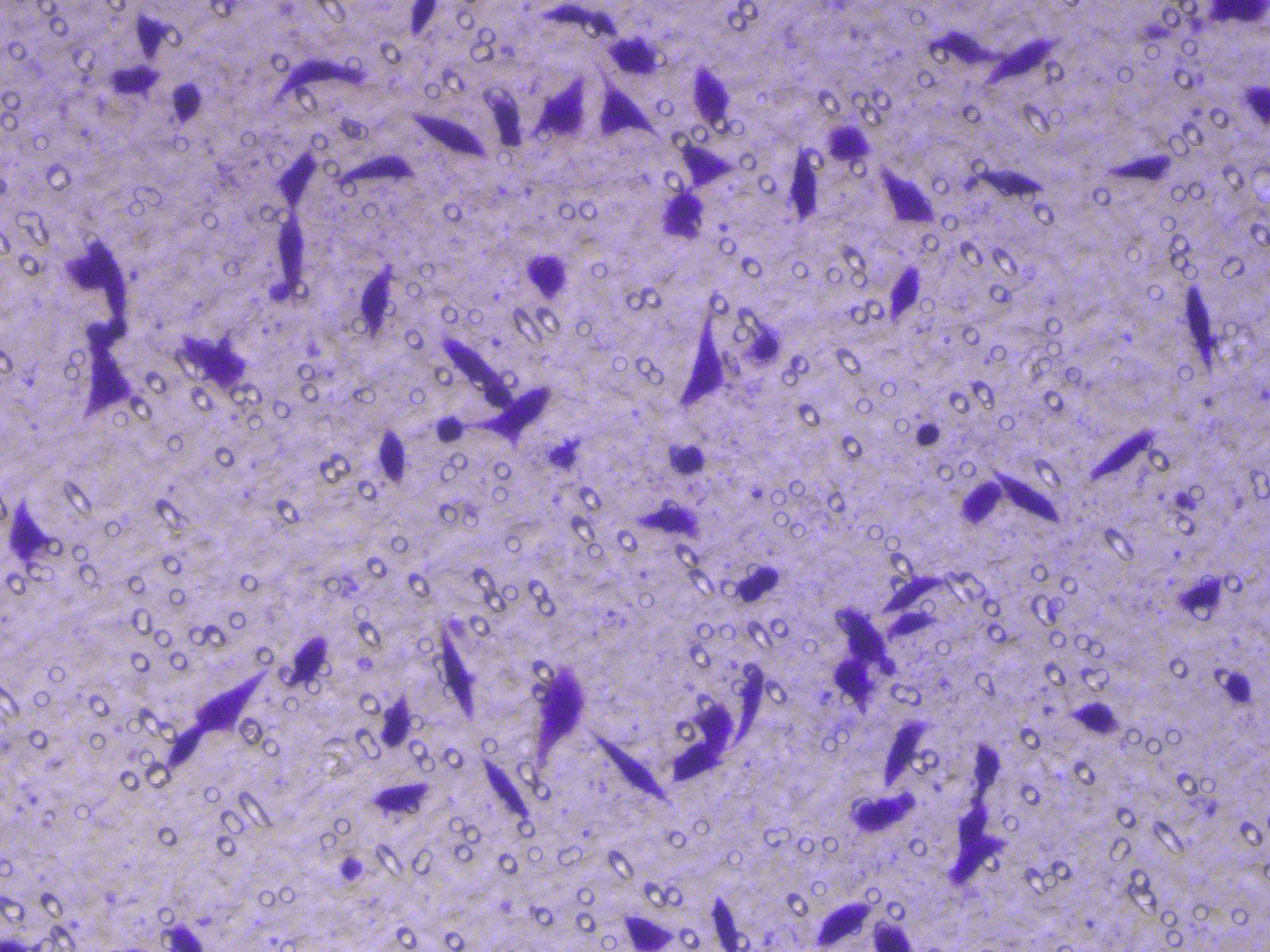

Supplement: Supplementary file 19 — Source Data [file 41467_2023_43282_MOESM19_ESM.zip › Source Data/Source data-Transwell raw images/Invasion/A OVCAR-3/shMPP7-rep3.jpg]

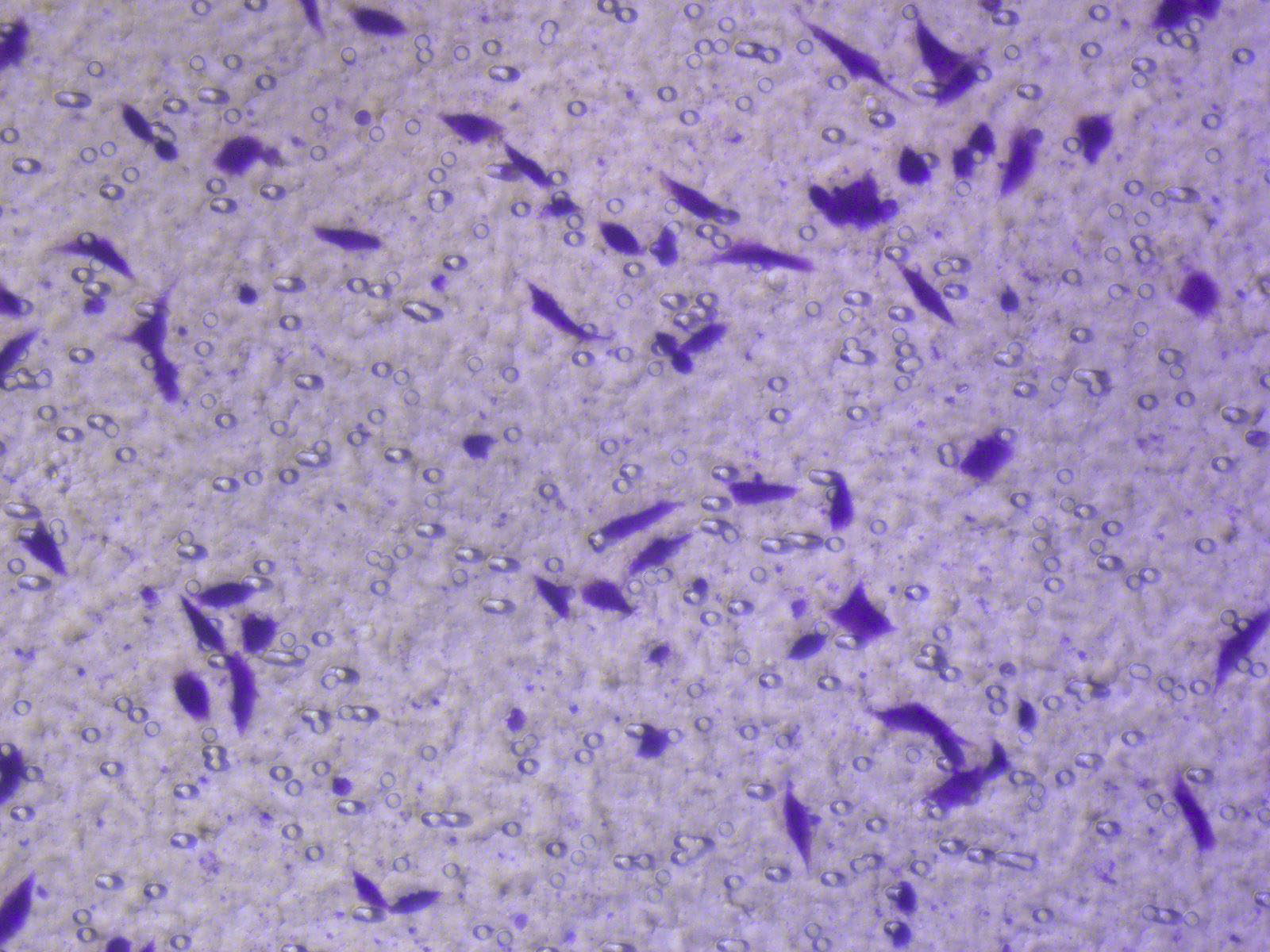

Supplement: Supplementary file 19 — Source Data [file 41467_2023_43282_MOESM19_ESM.zip › Source Data/Source data-Transwell raw images/Invasion/A OVCAR-3/shMPP7-rep4.jpg]

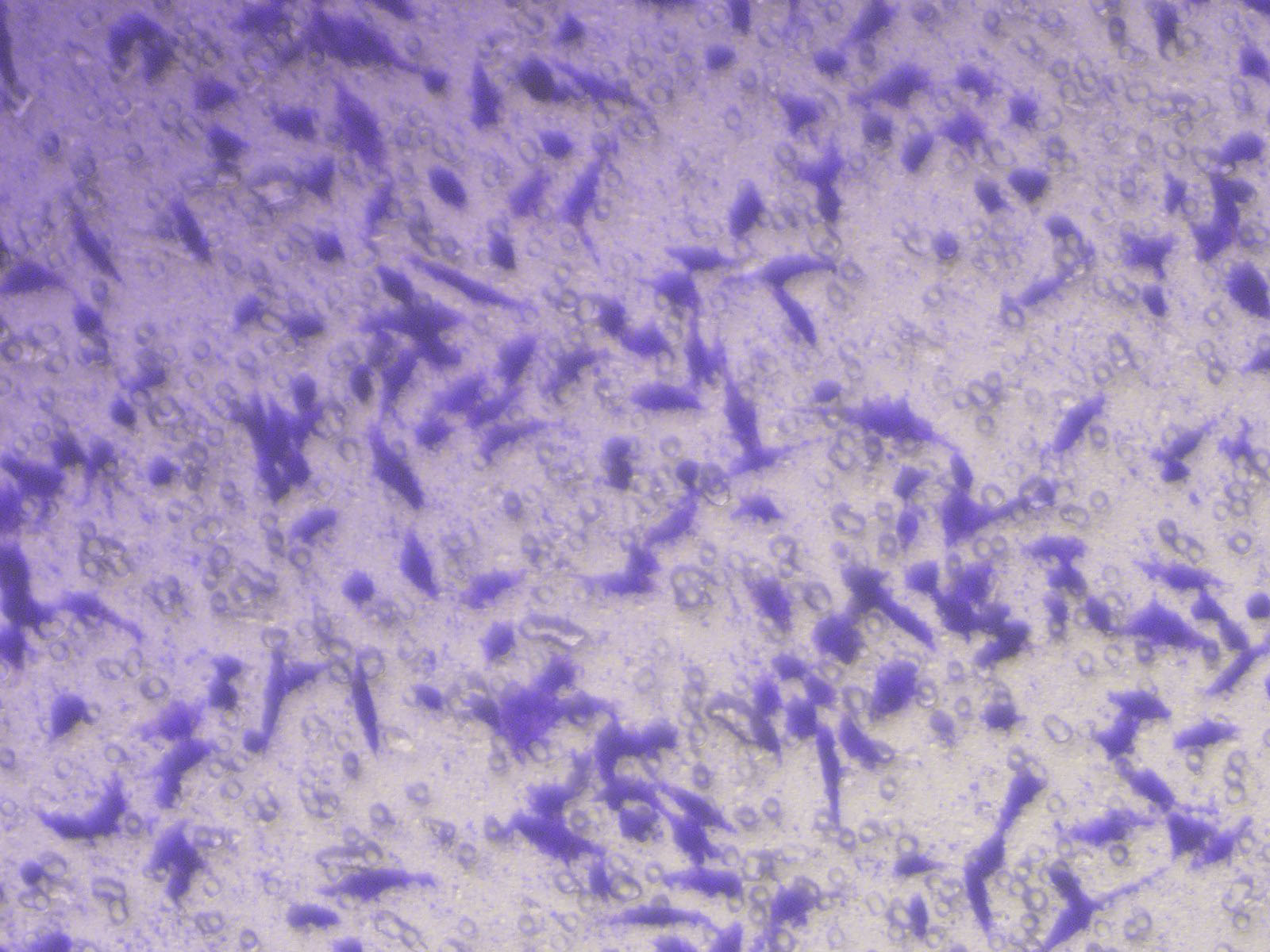

Supplement: Supplementary file 19 — Source Data [file 41467_2023_43282_MOESM19_ESM.zip › Source Data/Source data-Transwell raw images/Invasion/A OVCAR-3/shNC-rep1.jpg]

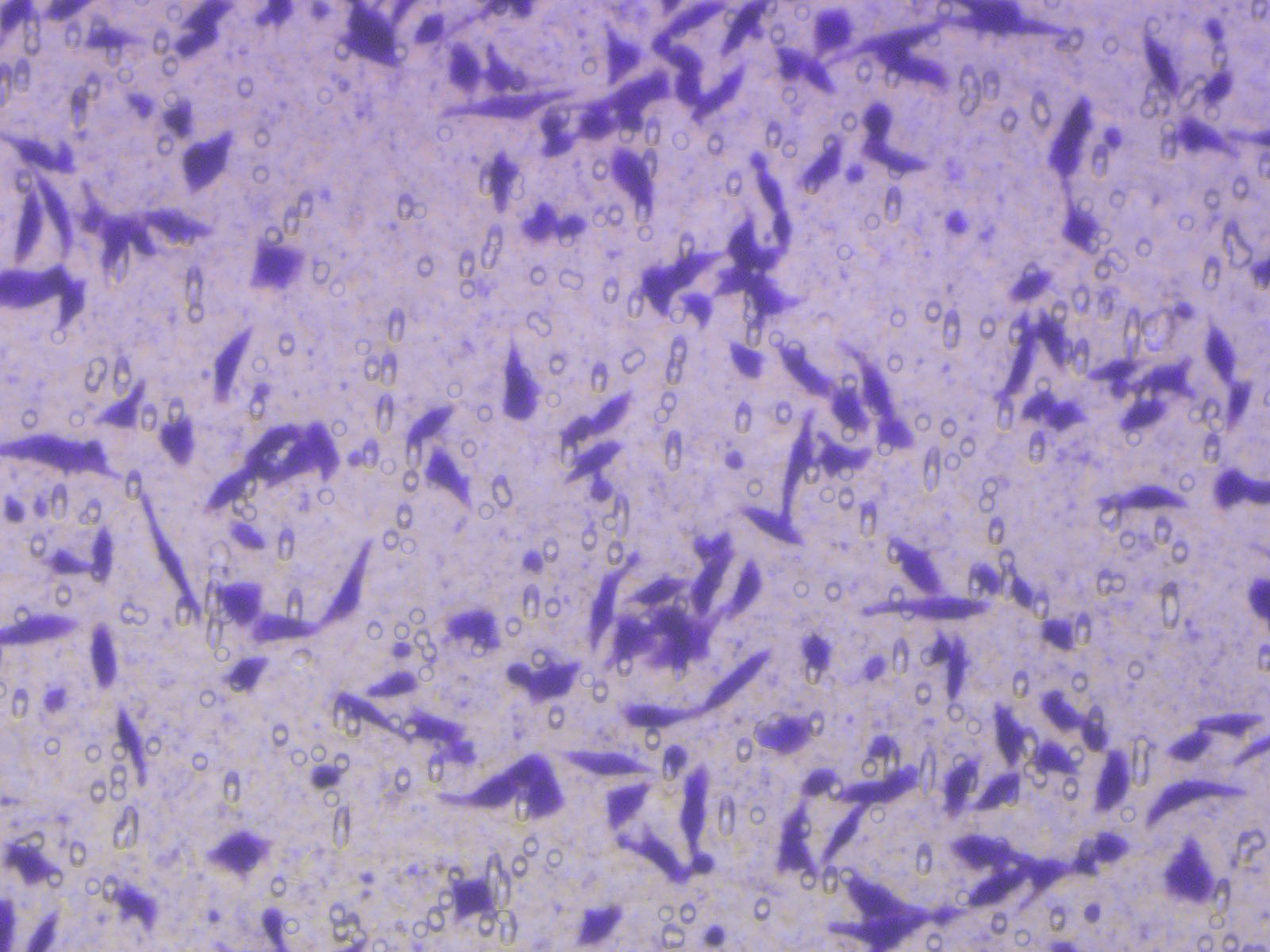

Supplement: Supplementary file 19 — Source Data [file 41467_2023_43282_MOESM19_ESM.zip › Source Data/Source data-Transwell raw images/Invasion/A OVCAR-3/shNC-rep2.jpg]

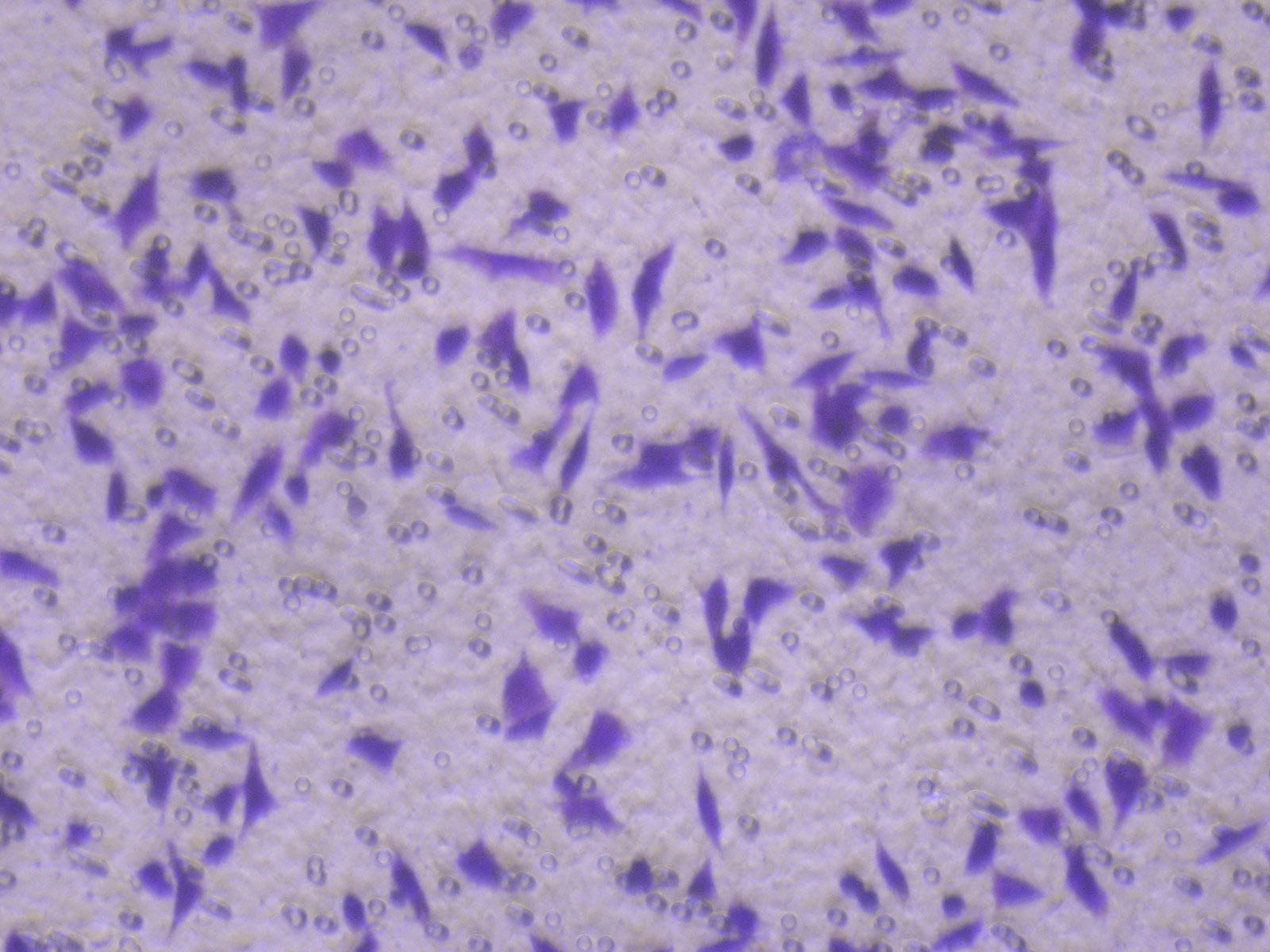

Supplement: Supplementary file 19 — Source Data [file 41467_2023_43282_MOESM19_ESM.zip › Source Data/Source data-Transwell raw images/Invasion/A OVCAR-3/shNC-rep3.jpg]

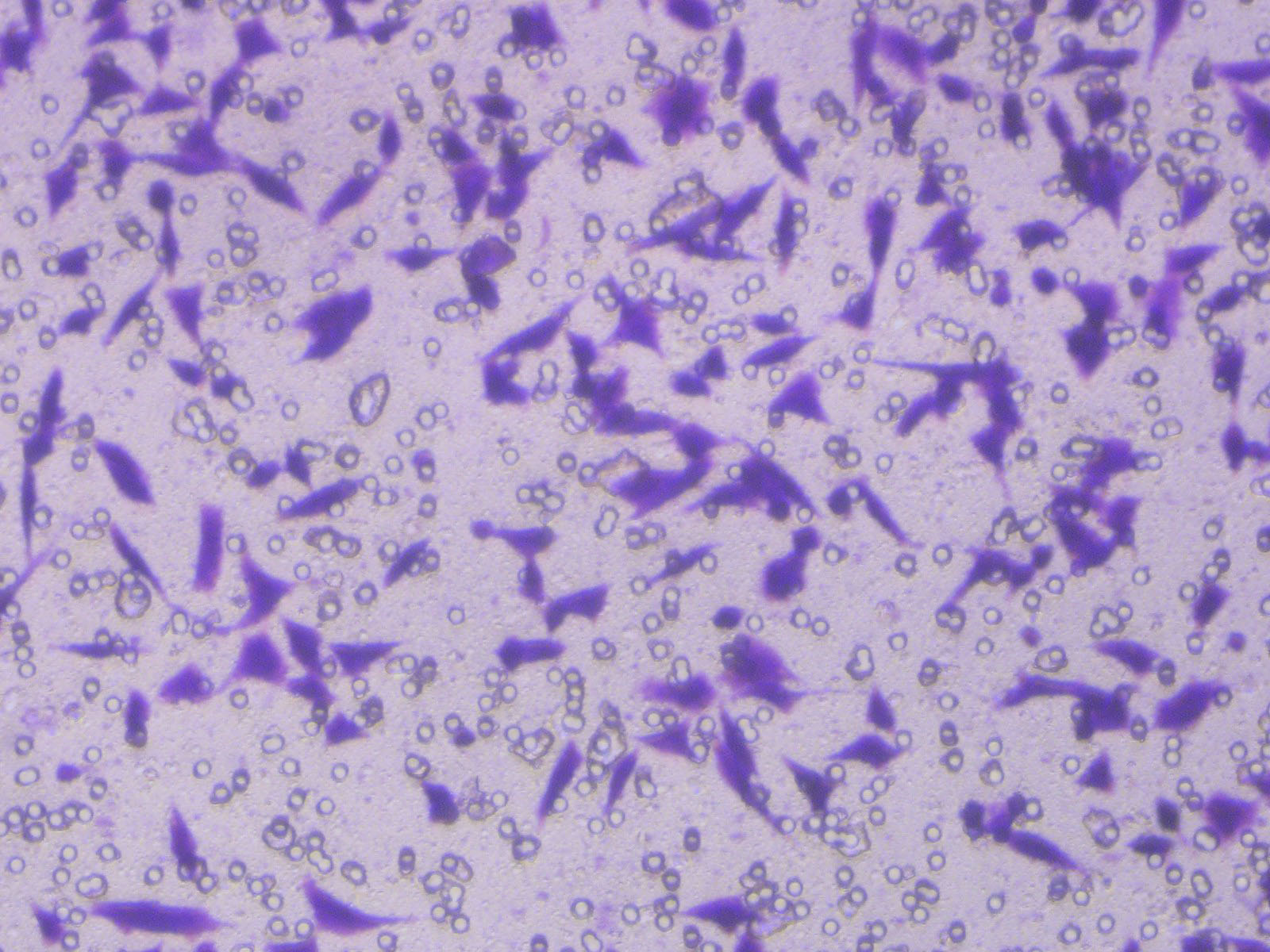

Supplement: Supplementary file 19 — Source Data [file 41467_2023_43282_MOESM19_ESM.zip › Source Data/Source data-Transwell raw images/Invasion/A OVCAR-3/shNC-rep4.jpg]

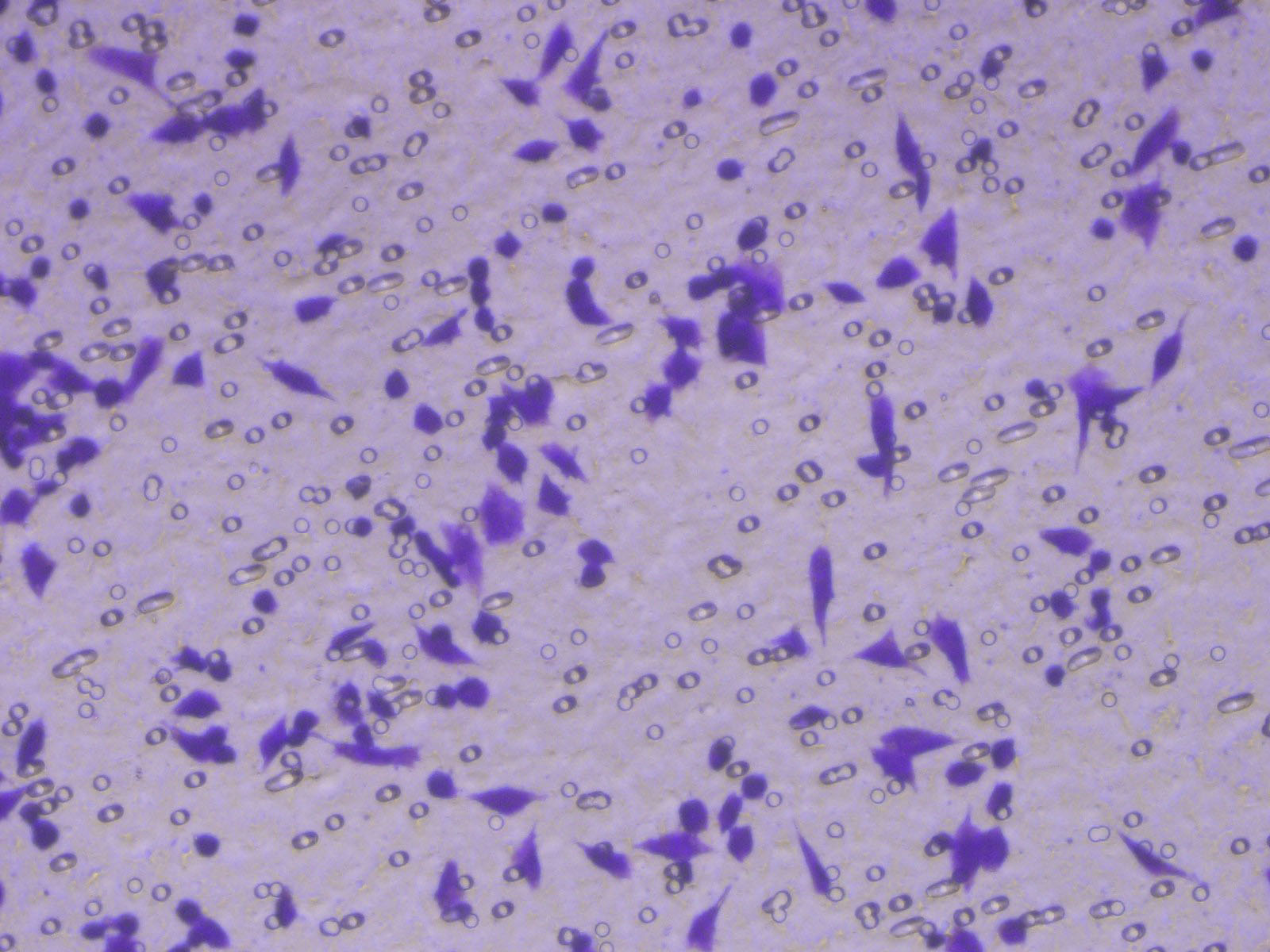

Supplement: Supplementary file 19 — Source Data [file 41467_2023_43282_MOESM19_ESM.zip › Source Data/Source data-Transwell raw images/Invasion/B A2780/Control-rep1.jpg]

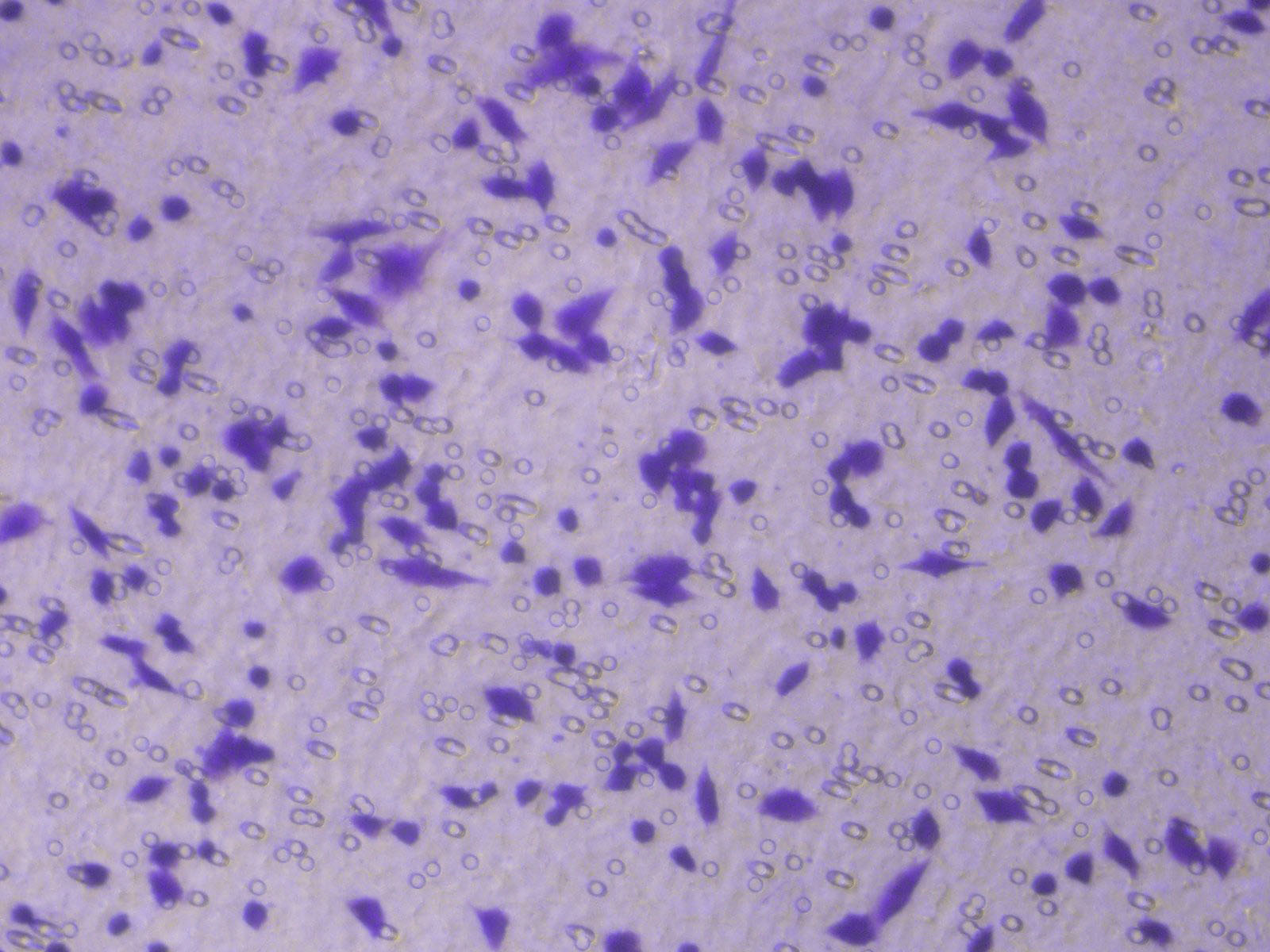

Supplement: Supplementary file 19 — Source Data [file 41467_2023_43282_MOESM19_ESM.zip › Source Data/Source data-Transwell raw images/Invasion/B A2780/Control-rep2.jpg]

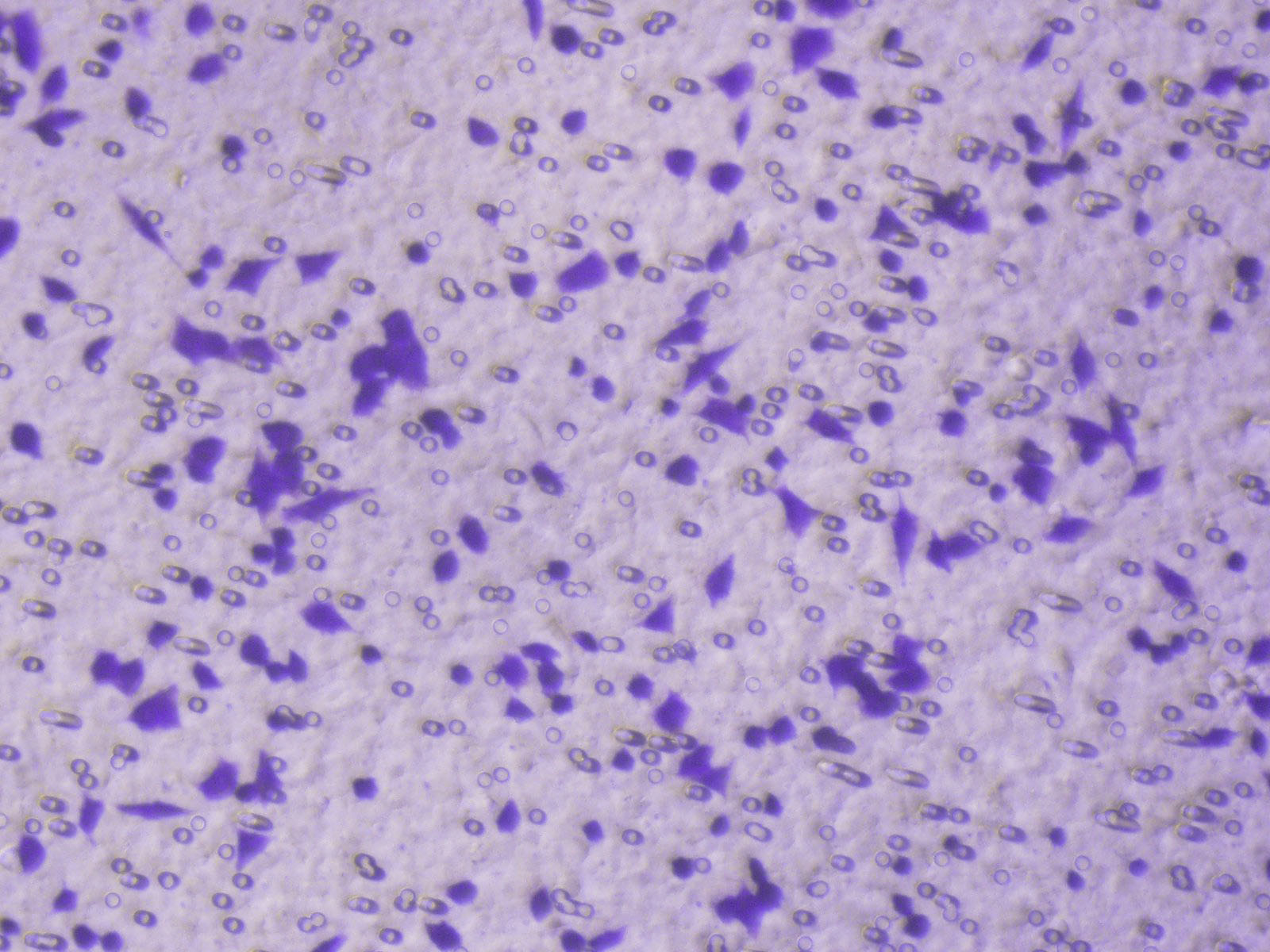

Supplement: Supplementary file 19 — Source Data [file 41467_2023_43282_MOESM19_ESM.zip › Source Data/Source data-Transwell raw images/Invasion/B A2780/Control-rep3.jpg]

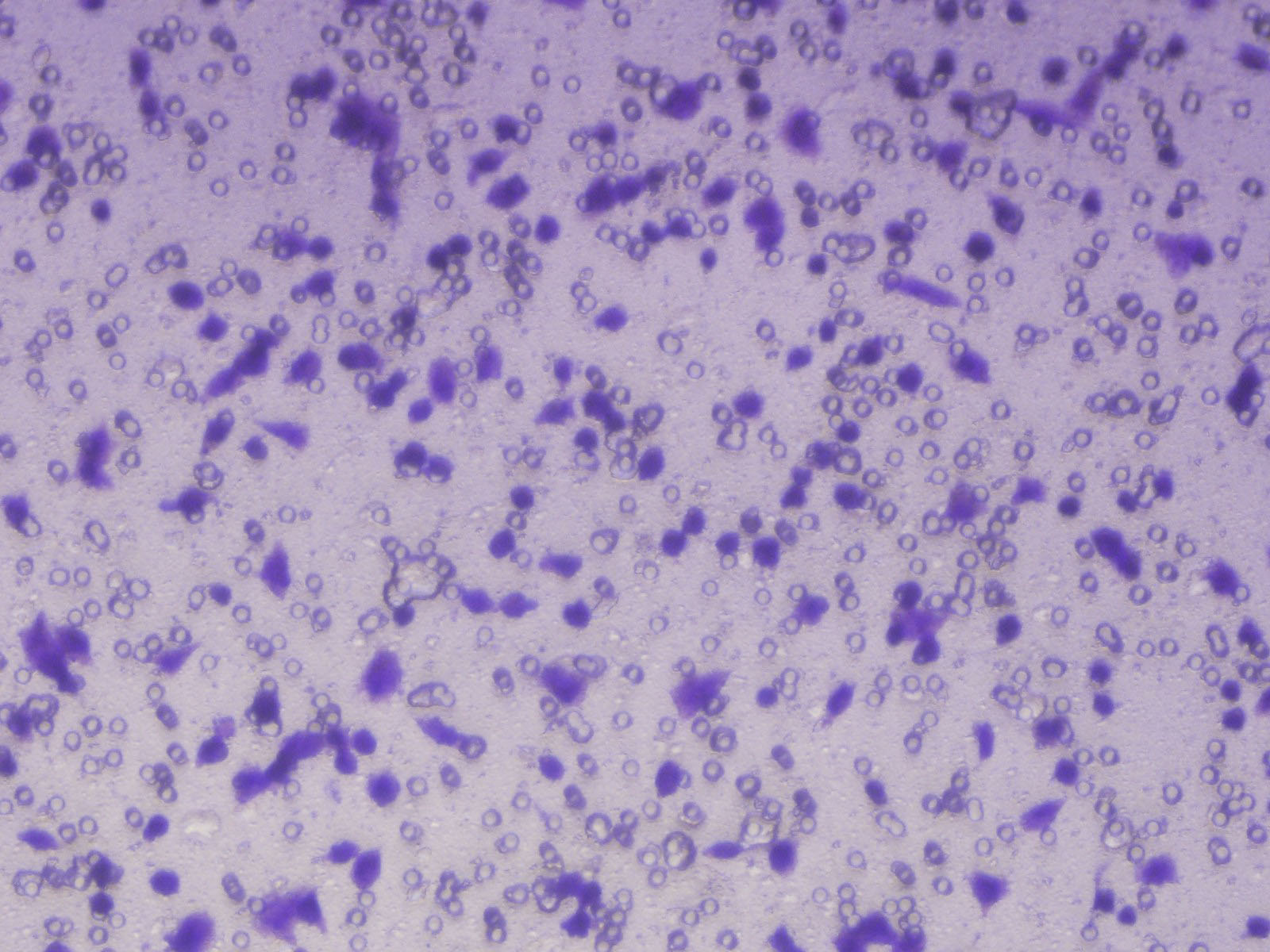

Supplement: Supplementary file 19 — Source Data [file 41467_2023_43282_MOESM19_ESM.zip › Source Data/Source data-Transwell raw images/Invasion/B A2780/Control-rep4.jpg]

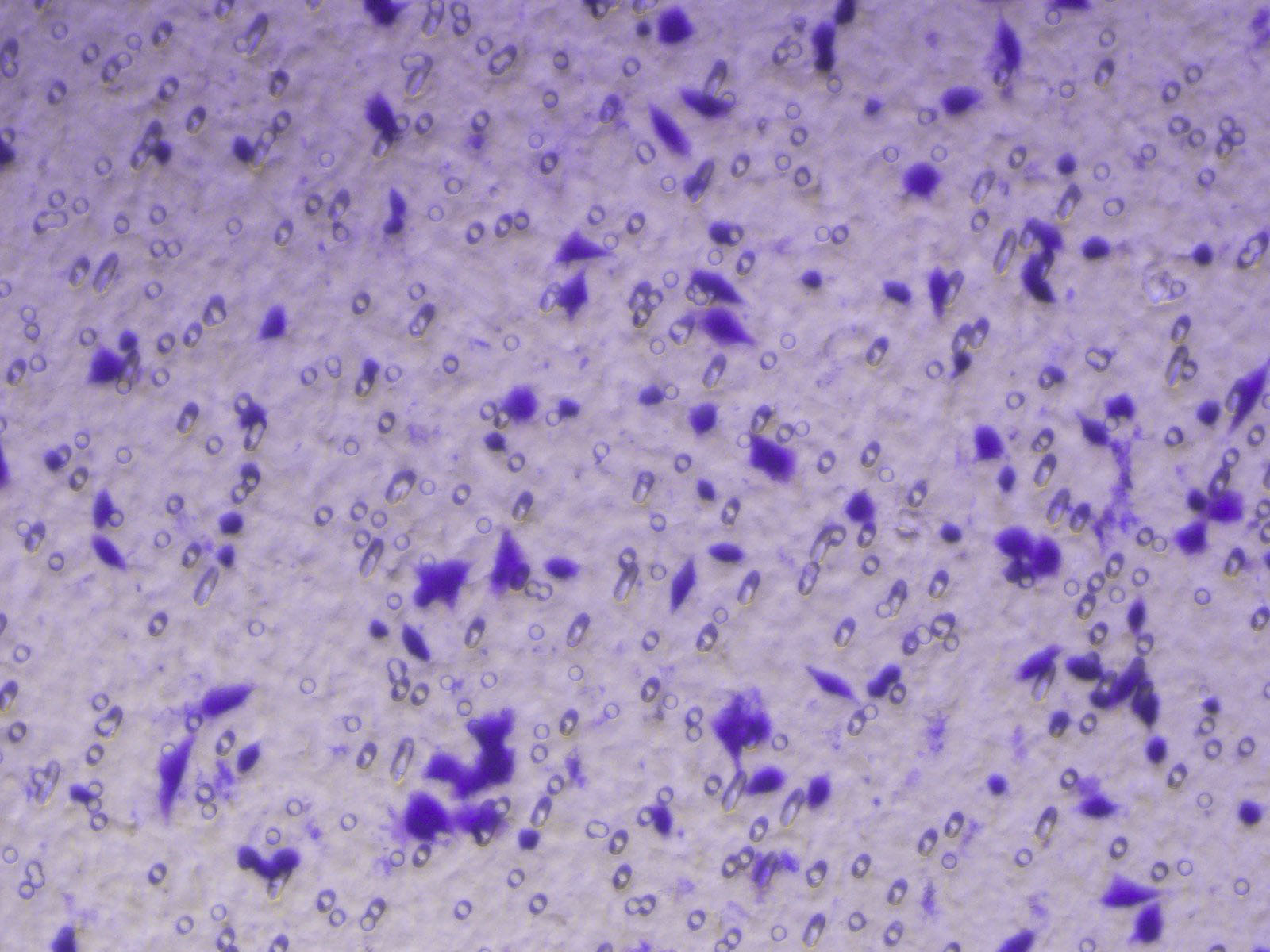

Supplement: Supplementary file 19 — Source Data [file 41467_2023_43282_MOESM19_ESM.zip › Source Data/Source data-Transwell raw images/Invasion/B A2780/shMPP7-rep1.jpg]

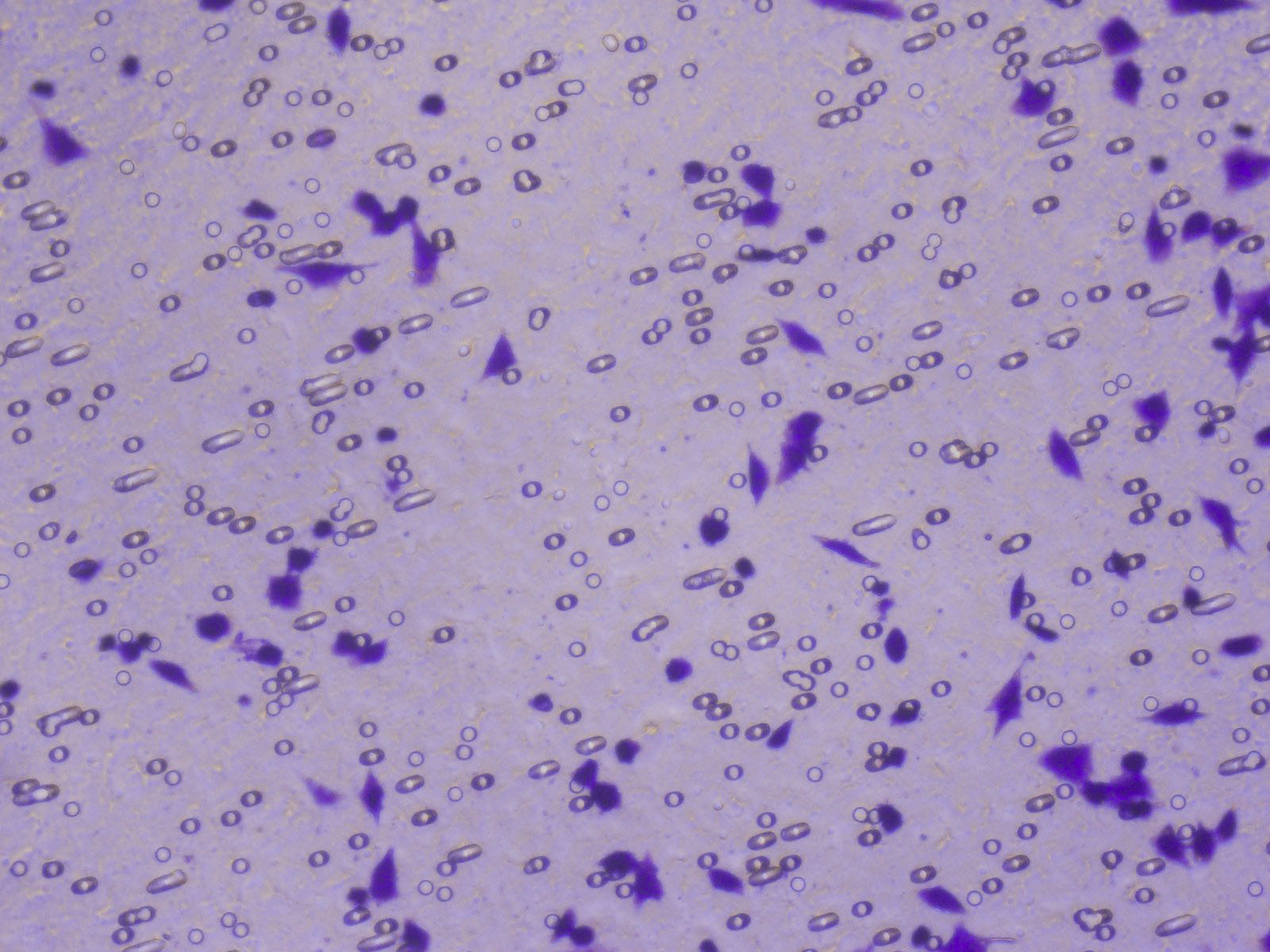

Supplement: Supplementary file 19 — Source Data [file 41467_2023_43282_MOESM19_ESM.zip › Source Data/Source data-Transwell raw images/Invasion/B A2780/shMPP7-rep2.jpg]

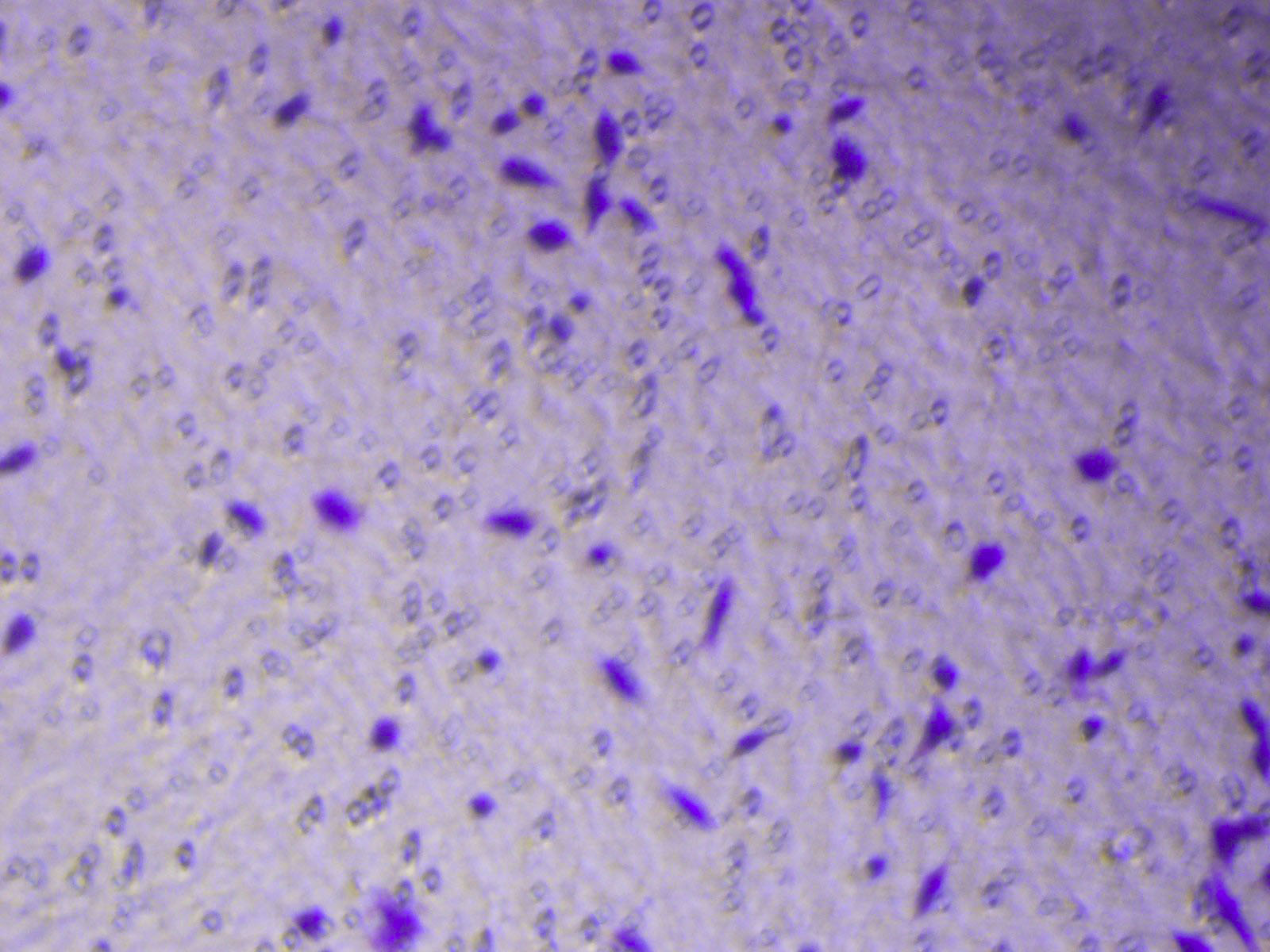

Supplement: Supplementary file 19 — Source Data [file 41467_2023_43282_MOESM19_ESM.zip › Source Data/Source data-Transwell raw images/Invasion/B A2780/shMPP7-rep3.jpg]

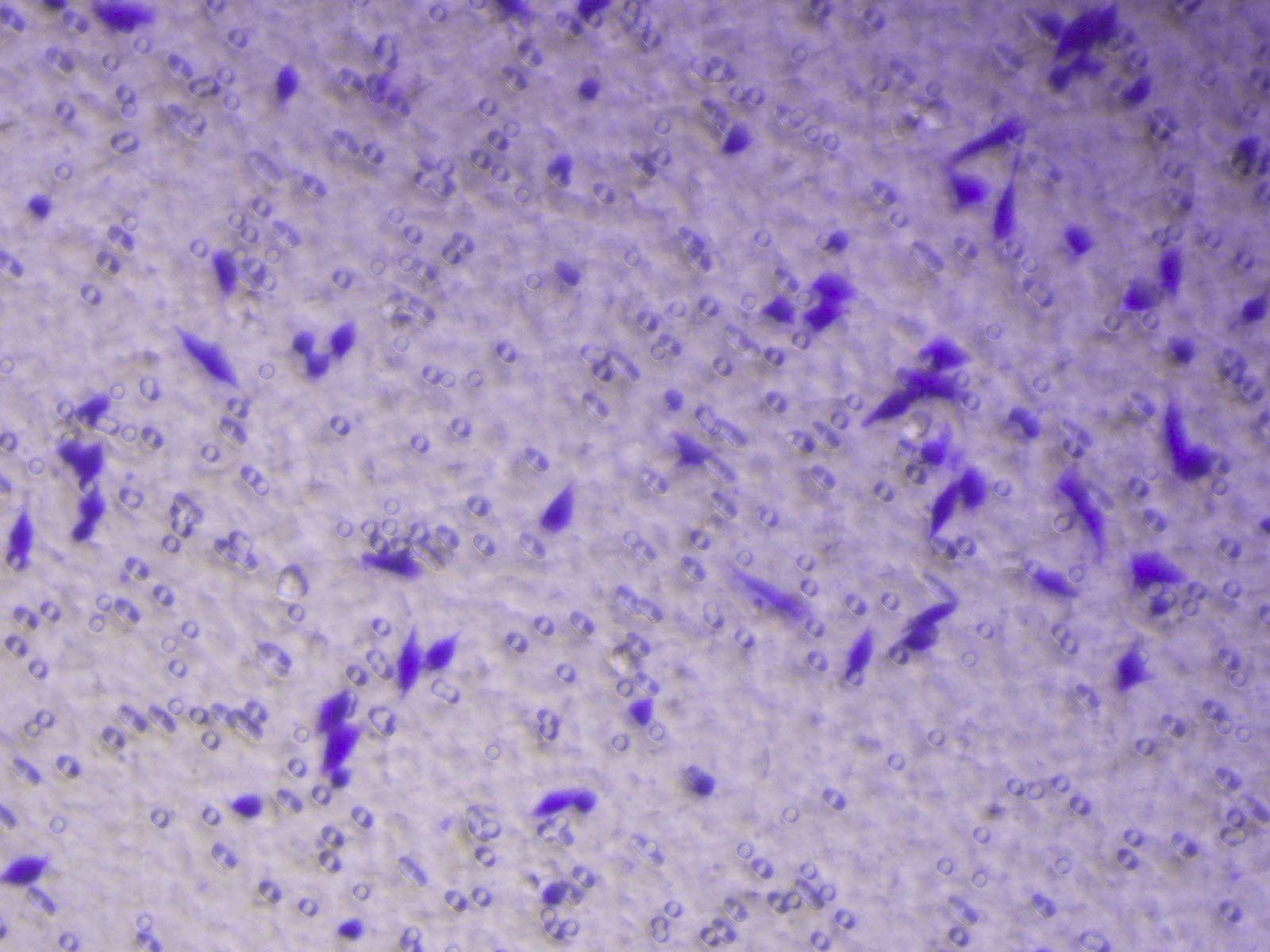

Supplement: Supplementary file 19 — Source Data [file 41467_2023_43282_MOESM19_ESM.zip › Source Data/Source data-Transwell raw images/Invasion/B A2780/shMPP7-rep4.jpg]

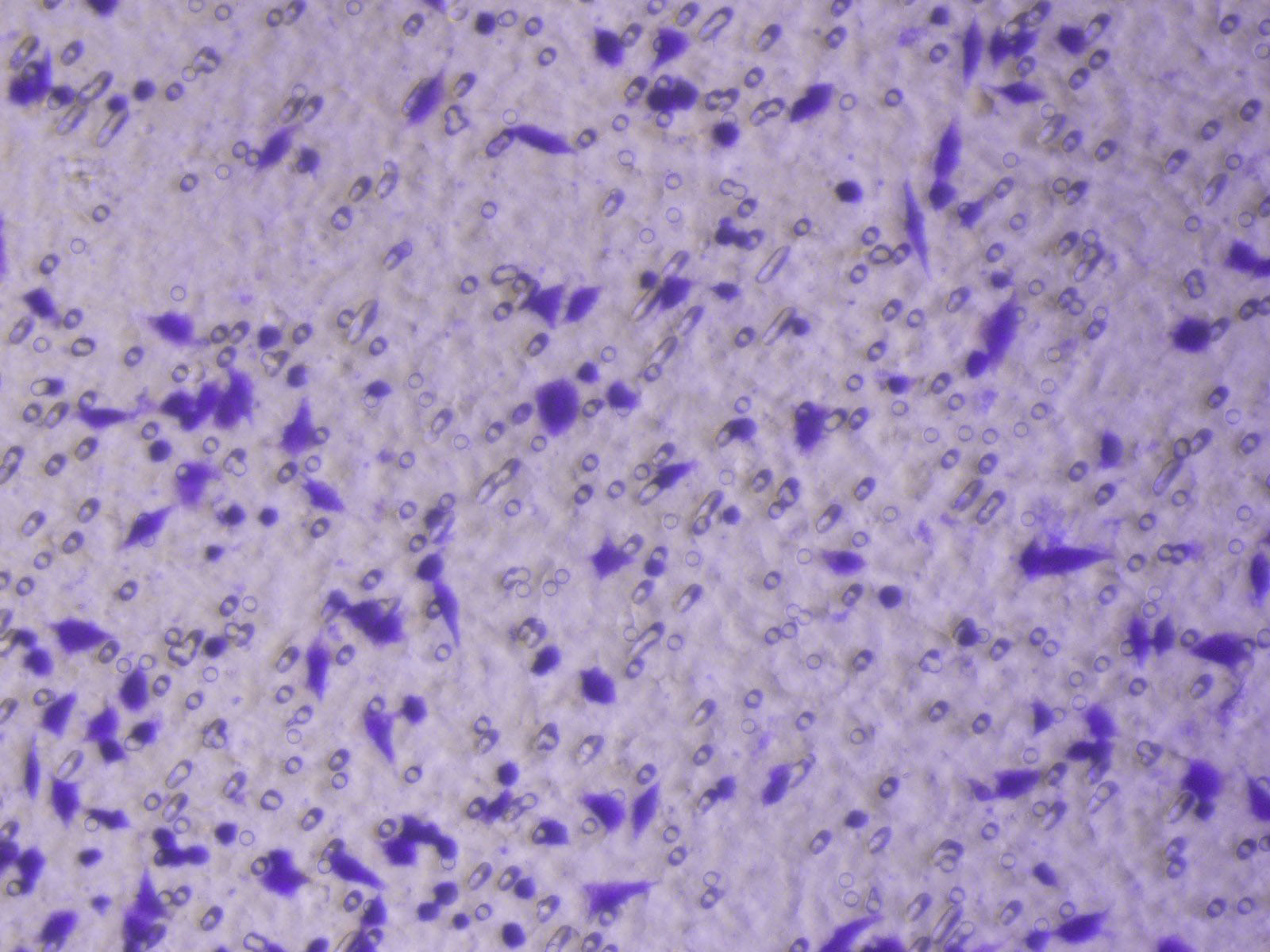

Supplement: Supplementary file 19 — Source Data [file 41467_2023_43282_MOESM19_ESM.zip › Source Data/Source data-Transwell raw images/Invasion/B A2780/shNC-rep1.jpg]

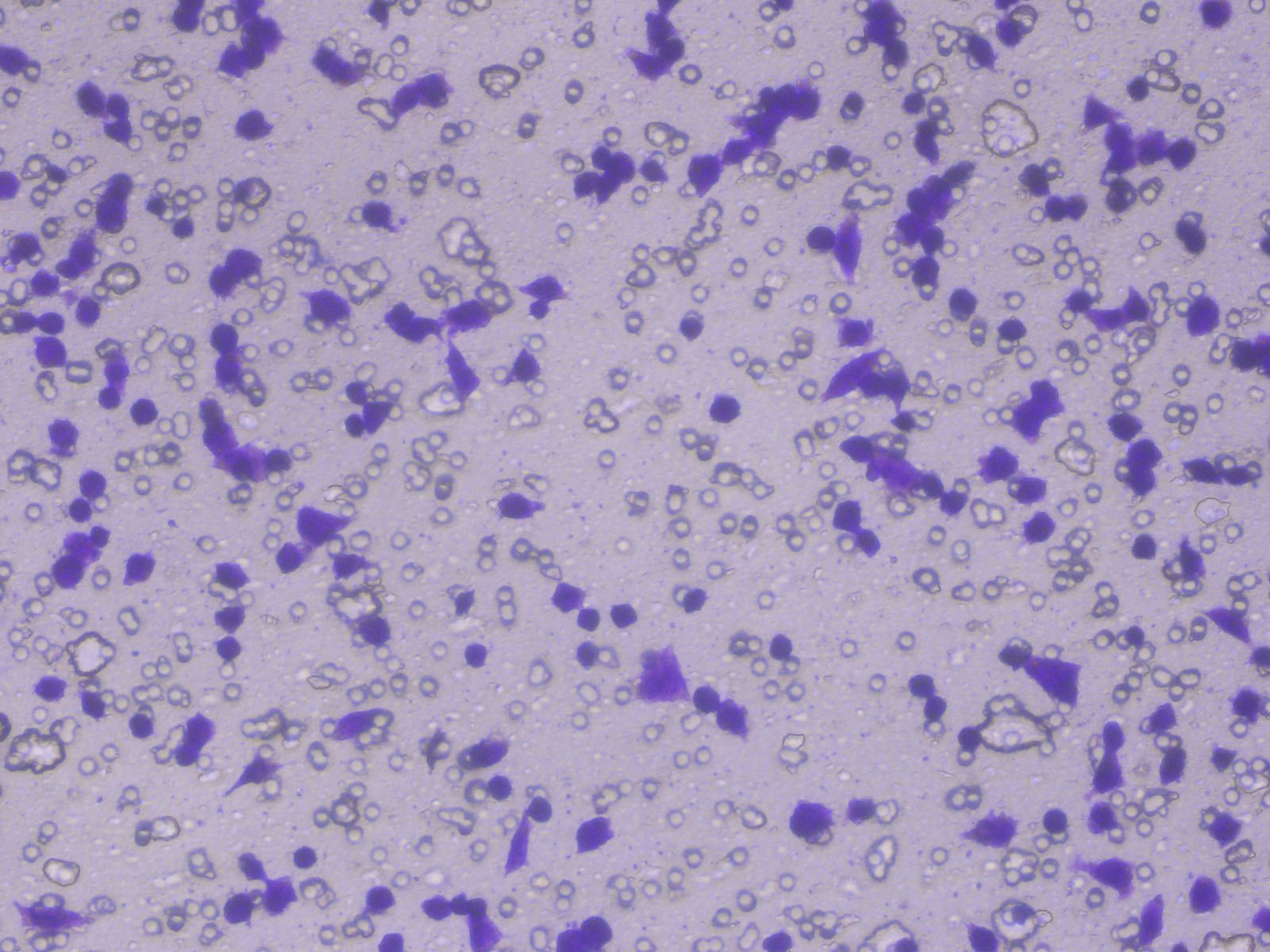

Supplement: Supplementary file 19 — Source Data [file 41467_2023_43282_MOESM19_ESM.zip › Source Data/Source data-Transwell raw images/Invasion/B A2780/shNC-rep2.jpg]

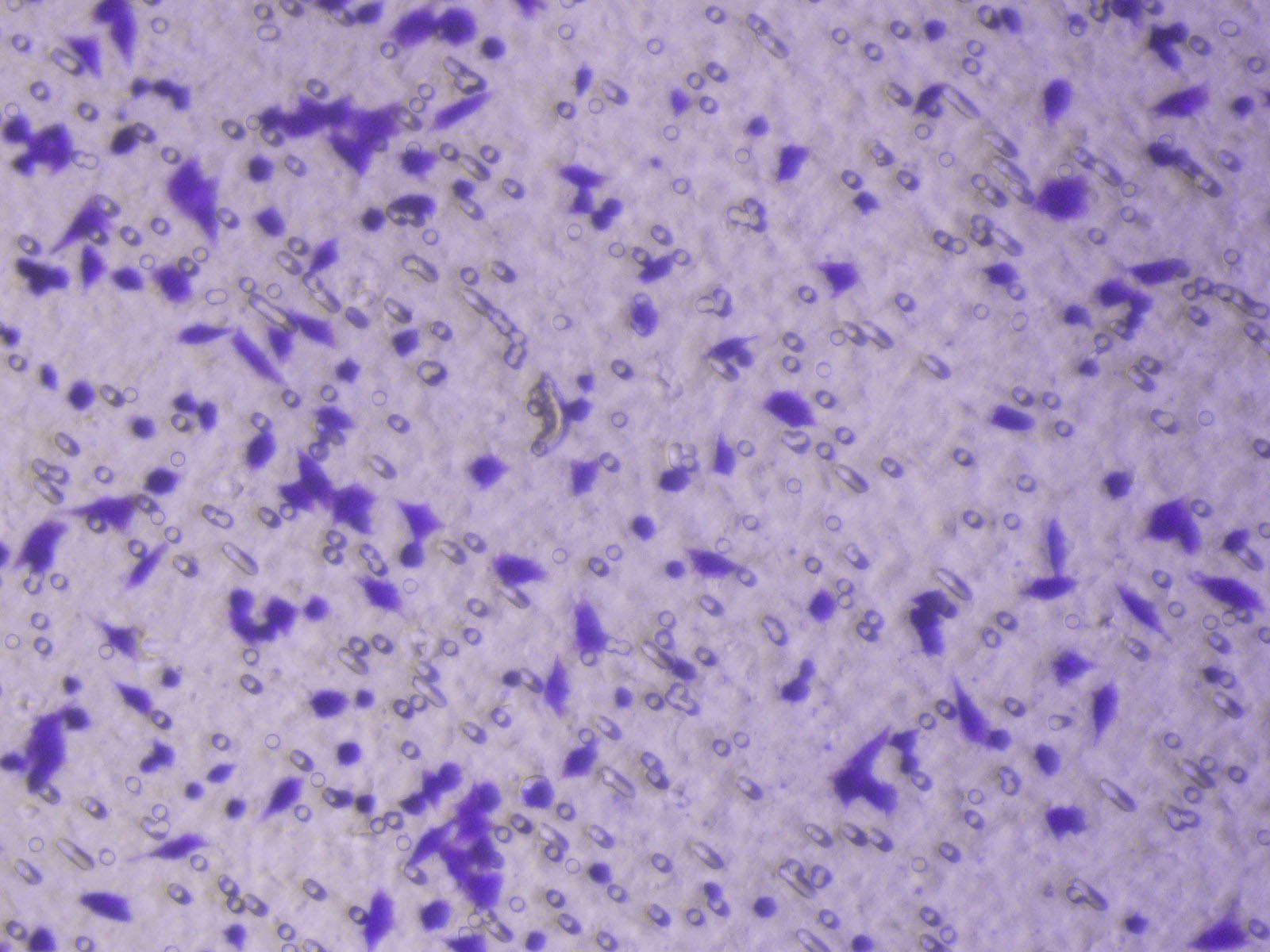

Supplement: Supplementary file 19 — Source Data [file 41467_2023_43282_MOESM19_ESM.zip › Source Data/Source data-Transwell raw images/Invasion/B A2780/shNC-rep3.jpg]

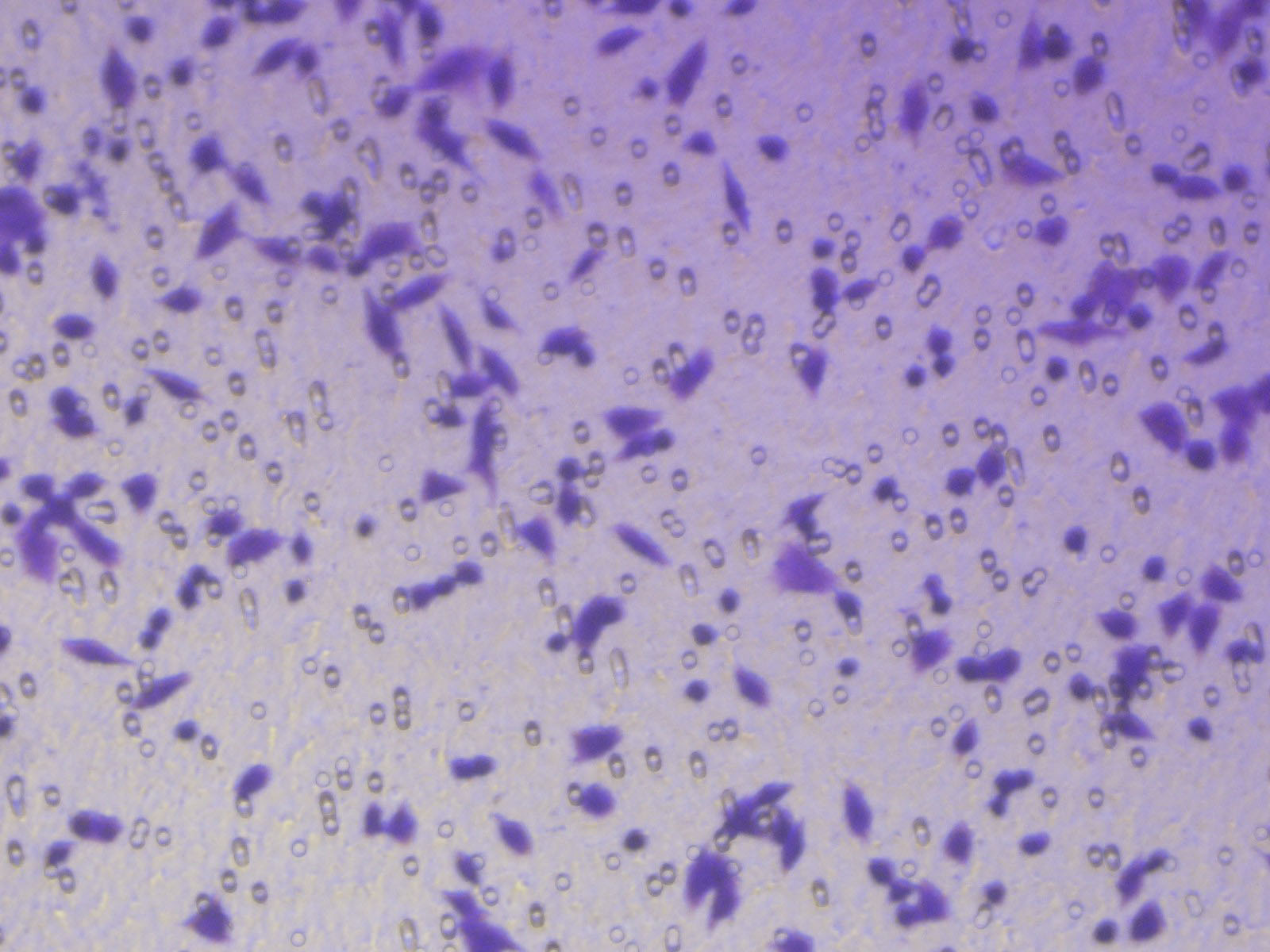

Supplement: Supplementary file 19 — Source Data [file 41467_2023_43282_MOESM19_ESM.zip › Source Data/Source data-Transwell raw images/Invasion/B A2780/shNC-rep4.jpg]

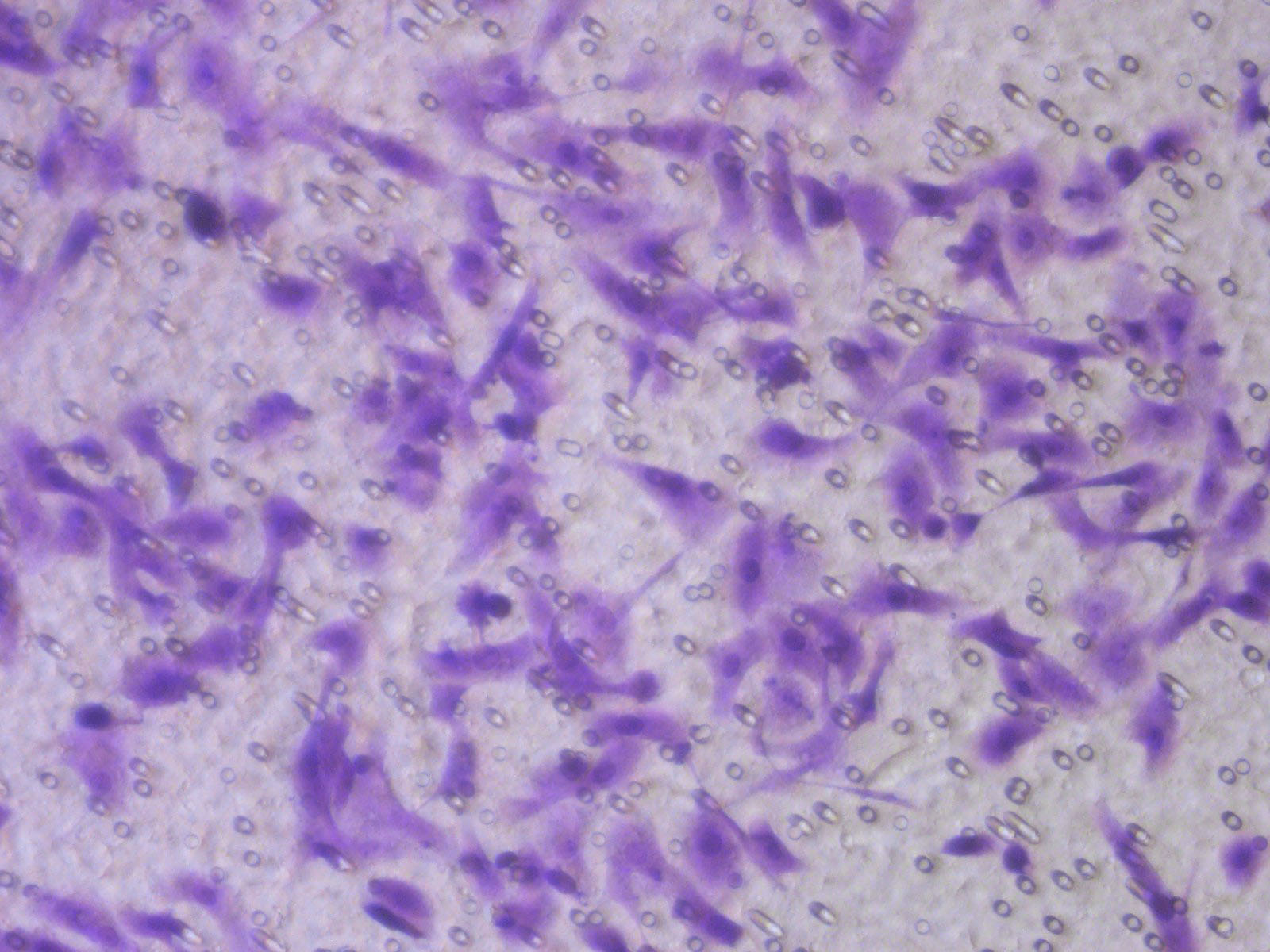

Supplement: Supplementary file 19 — Source Data [file 41467_2023_43282_MOESM19_ESM.zip › Source Data/Source data-Transwell raw images/Invasion/C ES-2/Control-rep1.jpg]

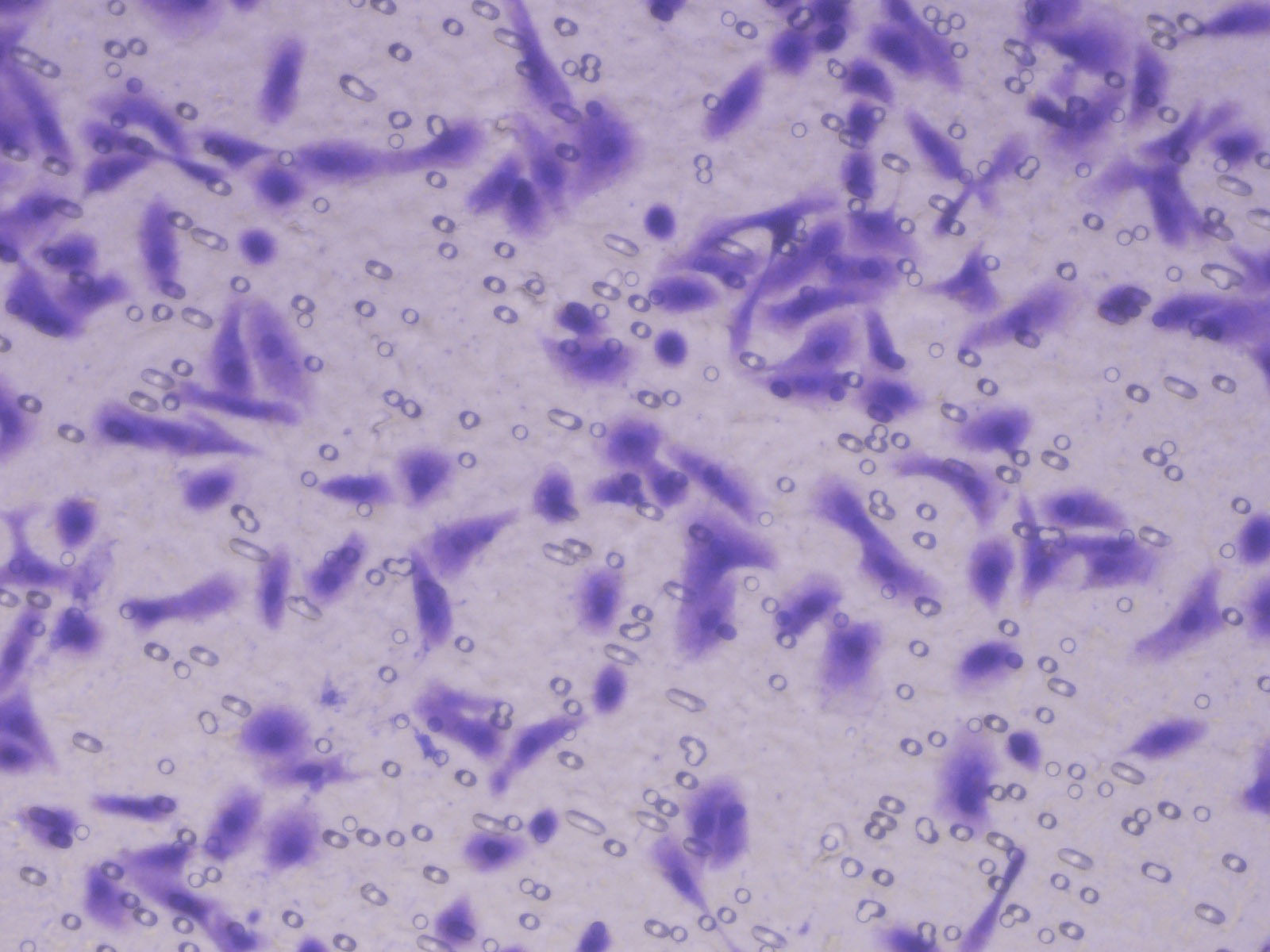

Supplement: Supplementary file 19 — Source Data [file 41467_2023_43282_MOESM19_ESM.zip › Source Data/Source data-Transwell raw images/Invasion/C ES-2/Control-rep2.jpg]

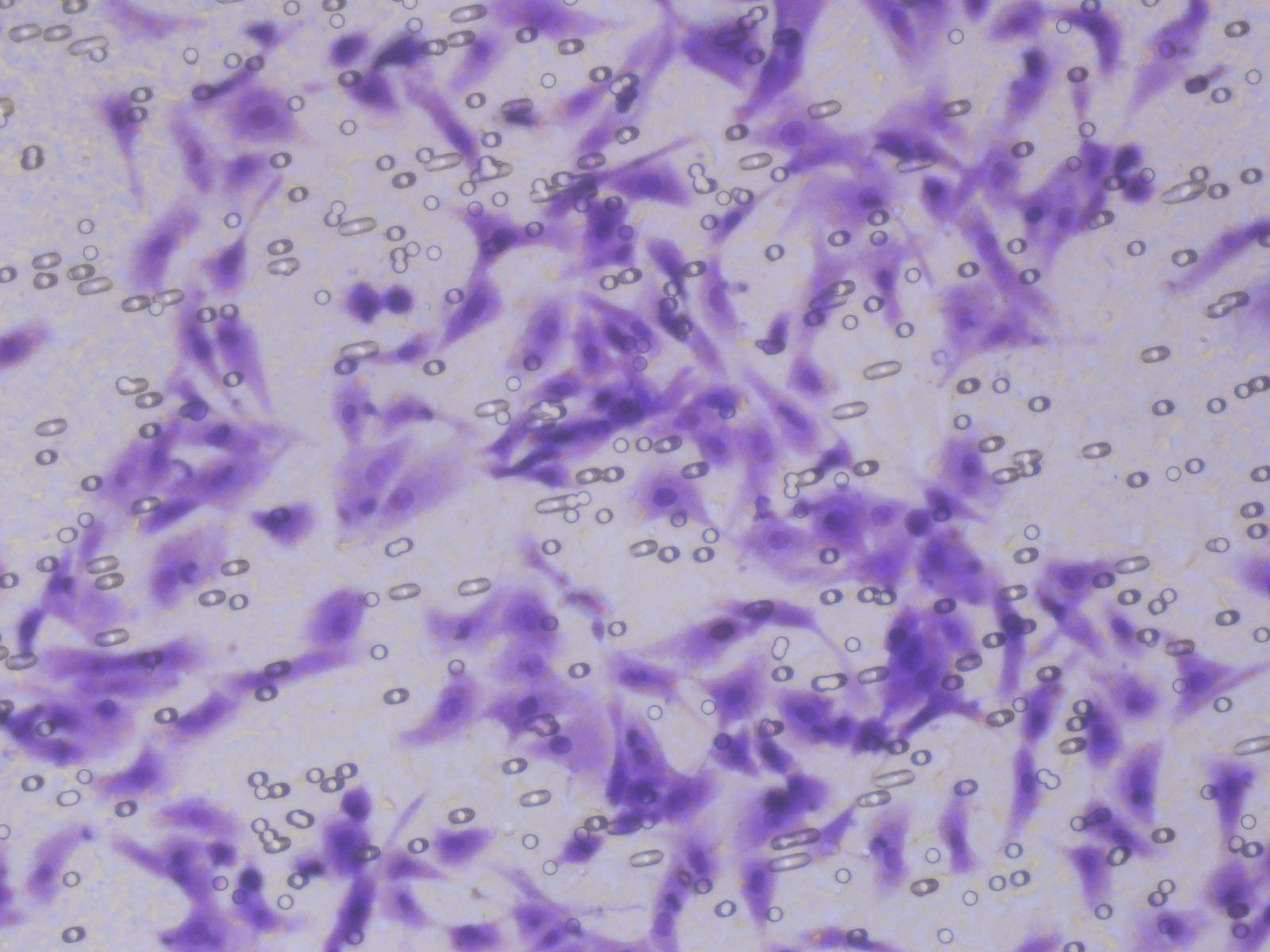

Supplement: Supplementary file 19 — Source Data [file 41467_2023_43282_MOESM19_ESM.zip › Source Data/Source data-Transwell raw images/Invasion/C ES-2/Control-rep3.jpg]

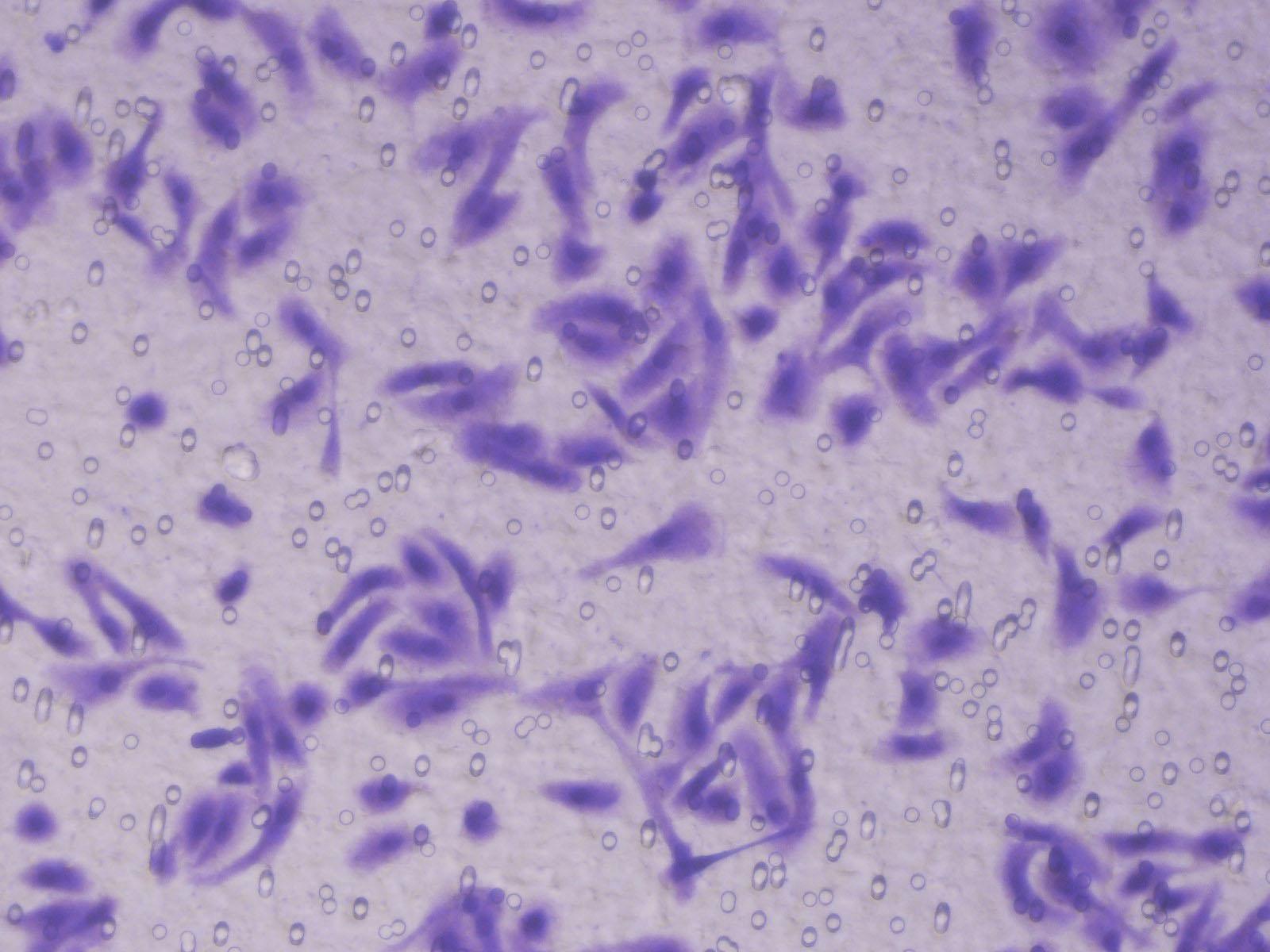

Supplement: Supplementary file 19 — Source Data [file 41467_2023_43282_MOESM19_ESM.zip › Source Data/Source data-Transwell raw images/Invasion/C ES-2/Control-rep4.jpg]

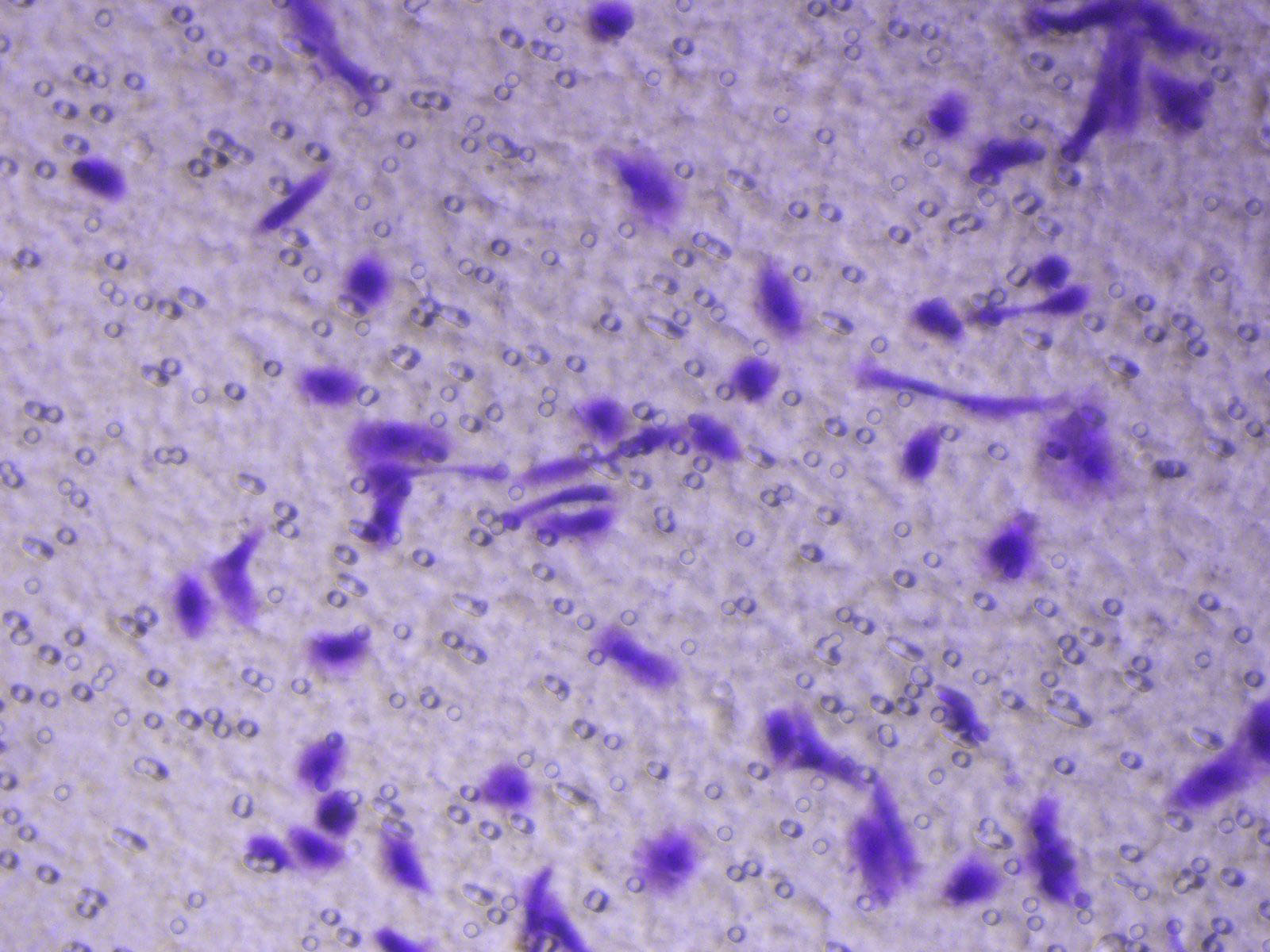

Supplement: Supplementary file 19 — Source Data [file 41467_2023_43282_MOESM19_ESM.zip › Source Data/Source data-Transwell raw images/Invasion/C ES-2/shMPP7-rep1.jpg]

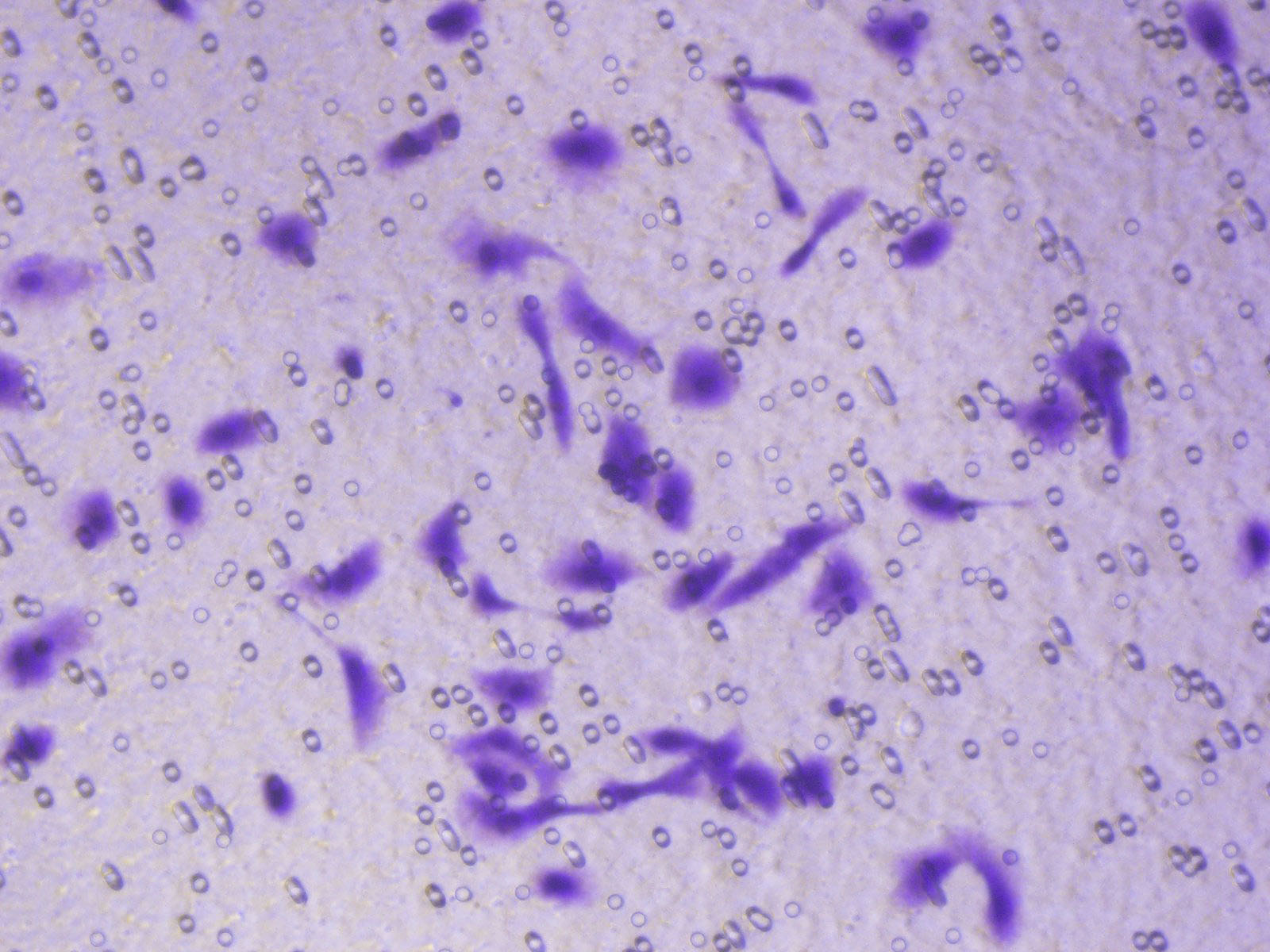

Supplement: Supplementary file 19 — Source Data [file 41467_2023_43282_MOESM19_ESM.zip › Source Data/Source data-Transwell raw images/Invasion/C ES-2/shMPP7-rep2.jpg]

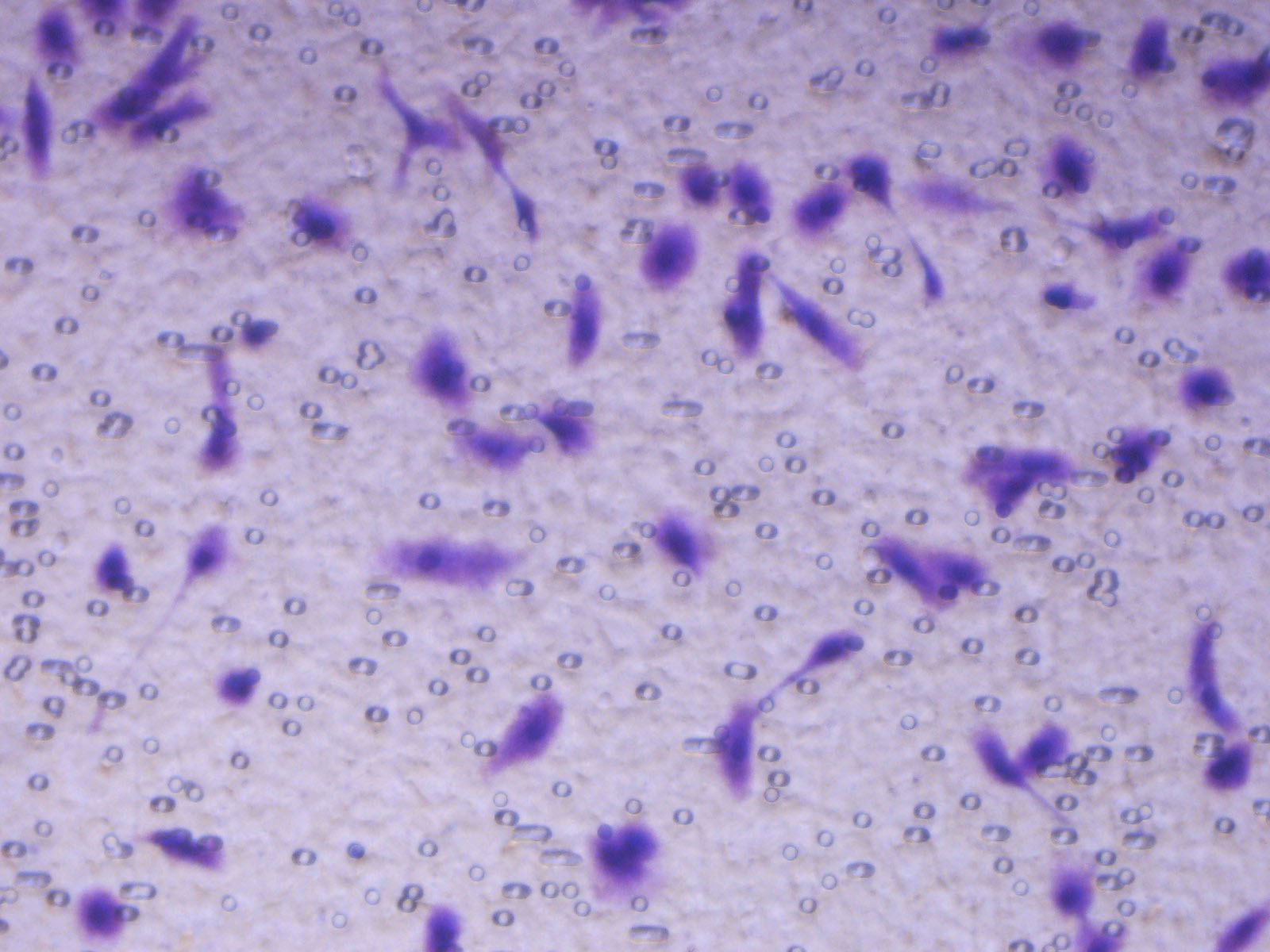

Supplement: Supplementary file 19 — Source Data [file 41467_2023_43282_MOESM19_ESM.zip › Source Data/Source data-Transwell raw images/Invasion/C ES-2/shMPP7-rep3.jpg]

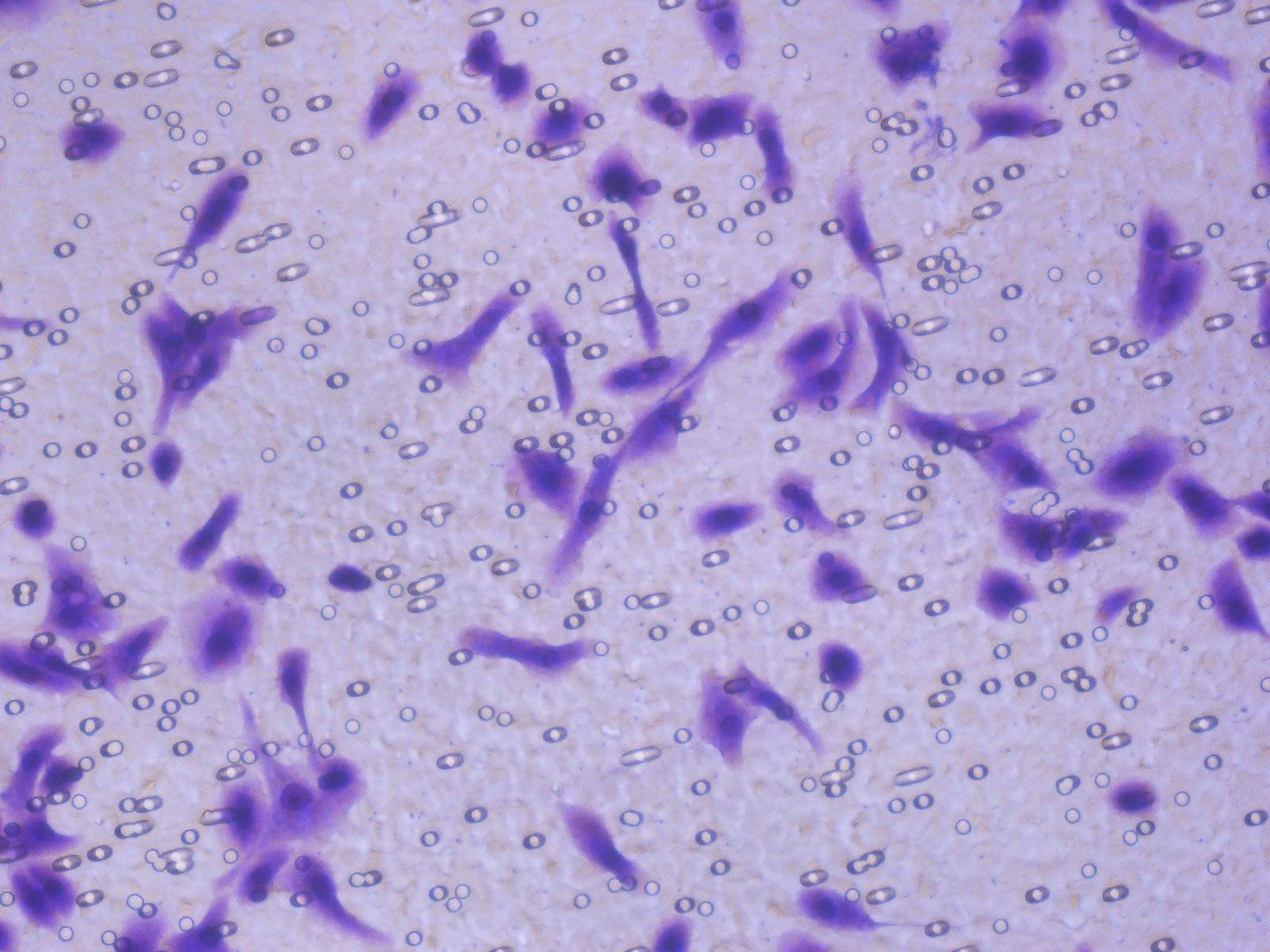

Supplement: Supplementary file 19 — Source Data [file 41467_2023_43282_MOESM19_ESM.zip › Source Data/Source data-Transwell raw images/Invasion/C ES-2/shMPP7-rep4.jpg]

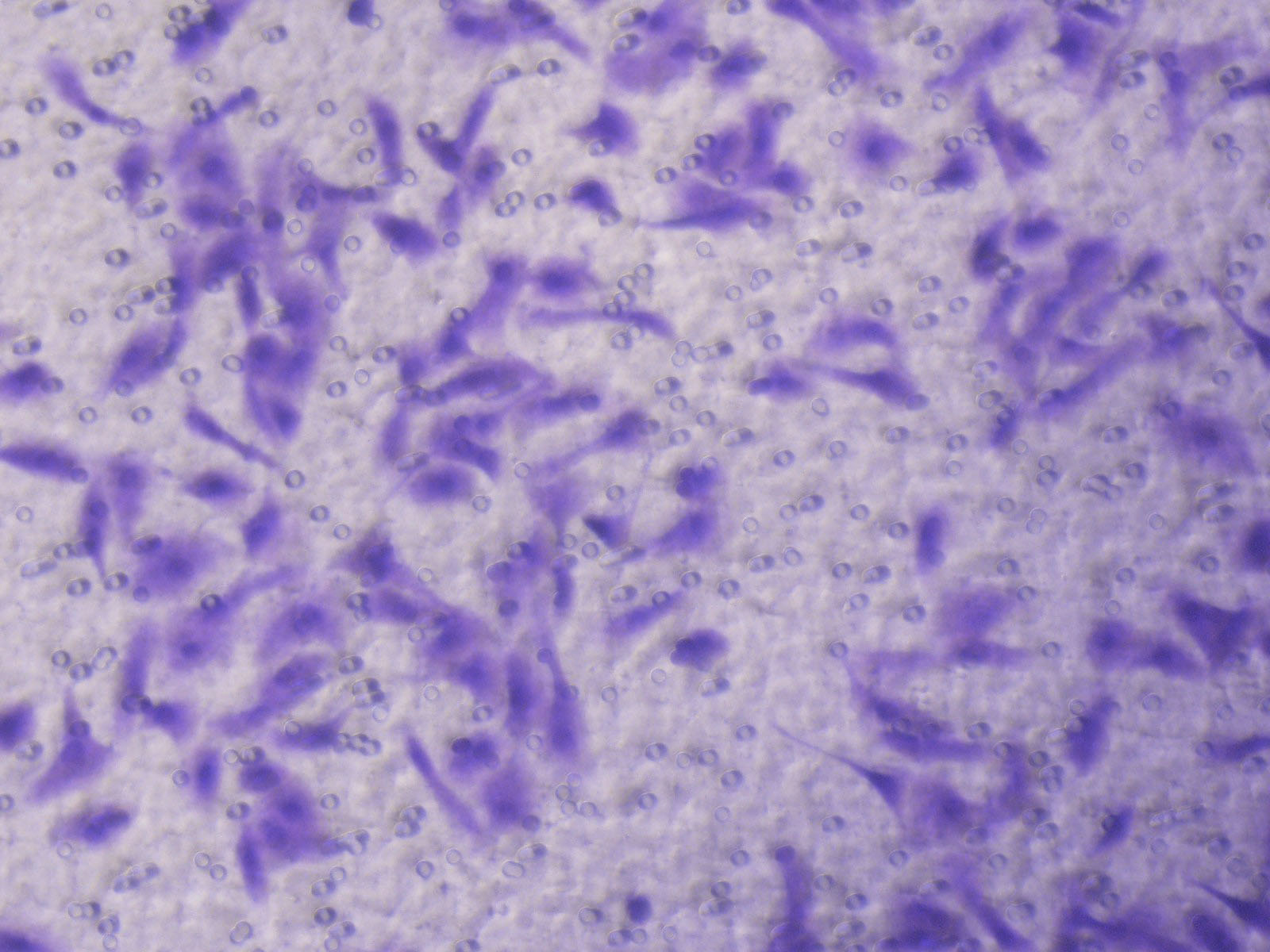

Supplement: Supplementary file 19 — Source Data [file 41467_2023_43282_MOESM19_ESM.zip › Source Data/Source data-Transwell raw images/Invasion/C ES-2/shNC-rep1.jpg]

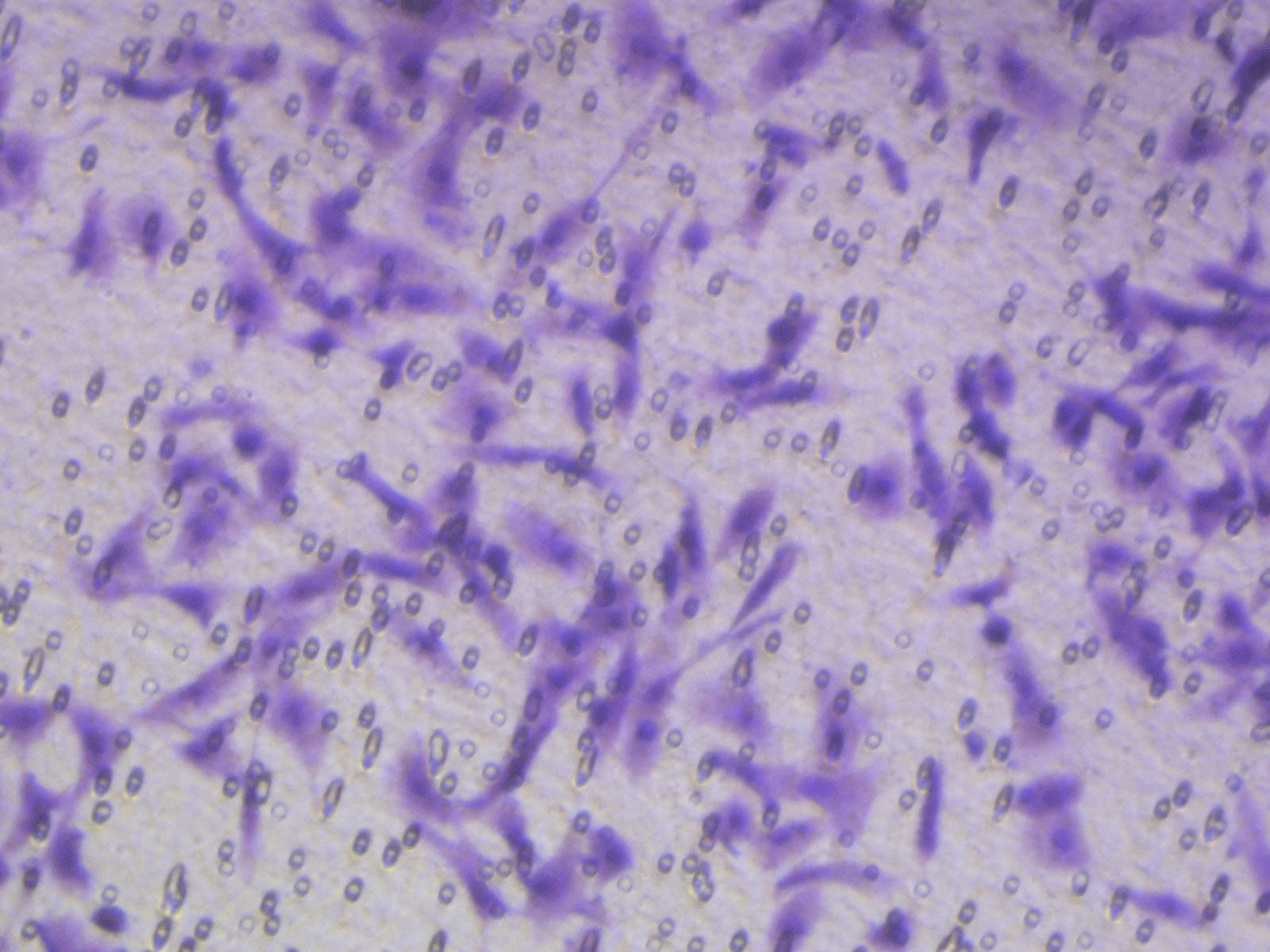

Supplement: Supplementary file 19 — Source Data [file 41467_2023_43282_MOESM19_ESM.zip › Source Data/Source data-Transwell raw images/Invasion/C ES-2/shNC-rep2.jpg]

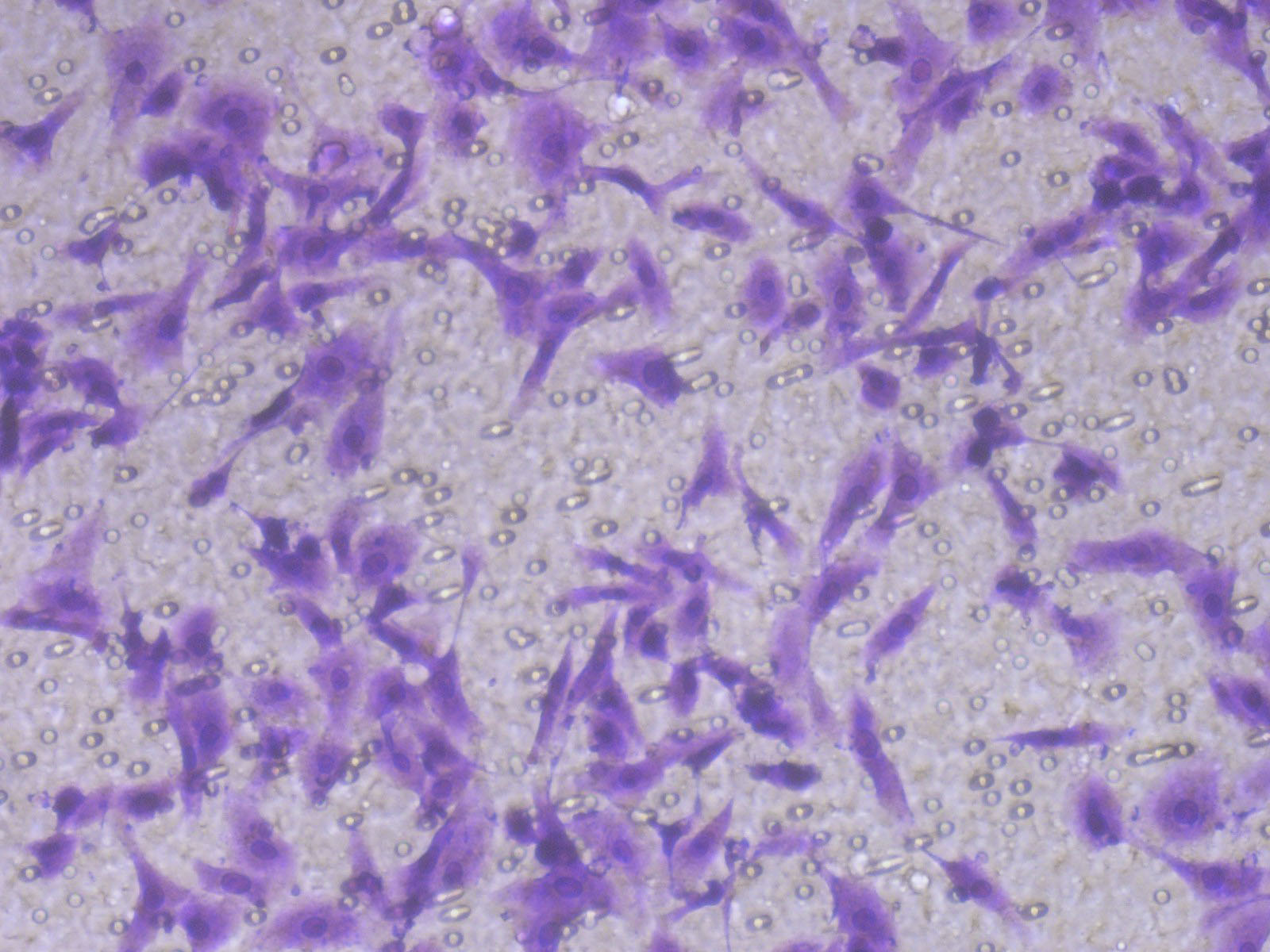

Supplement: Supplementary file 19 — Source Data [file 41467_2023_43282_MOESM19_ESM.zip › Source Data/Source data-Transwell raw images/Invasion/C ES-2/shNC-rep3.jpg]

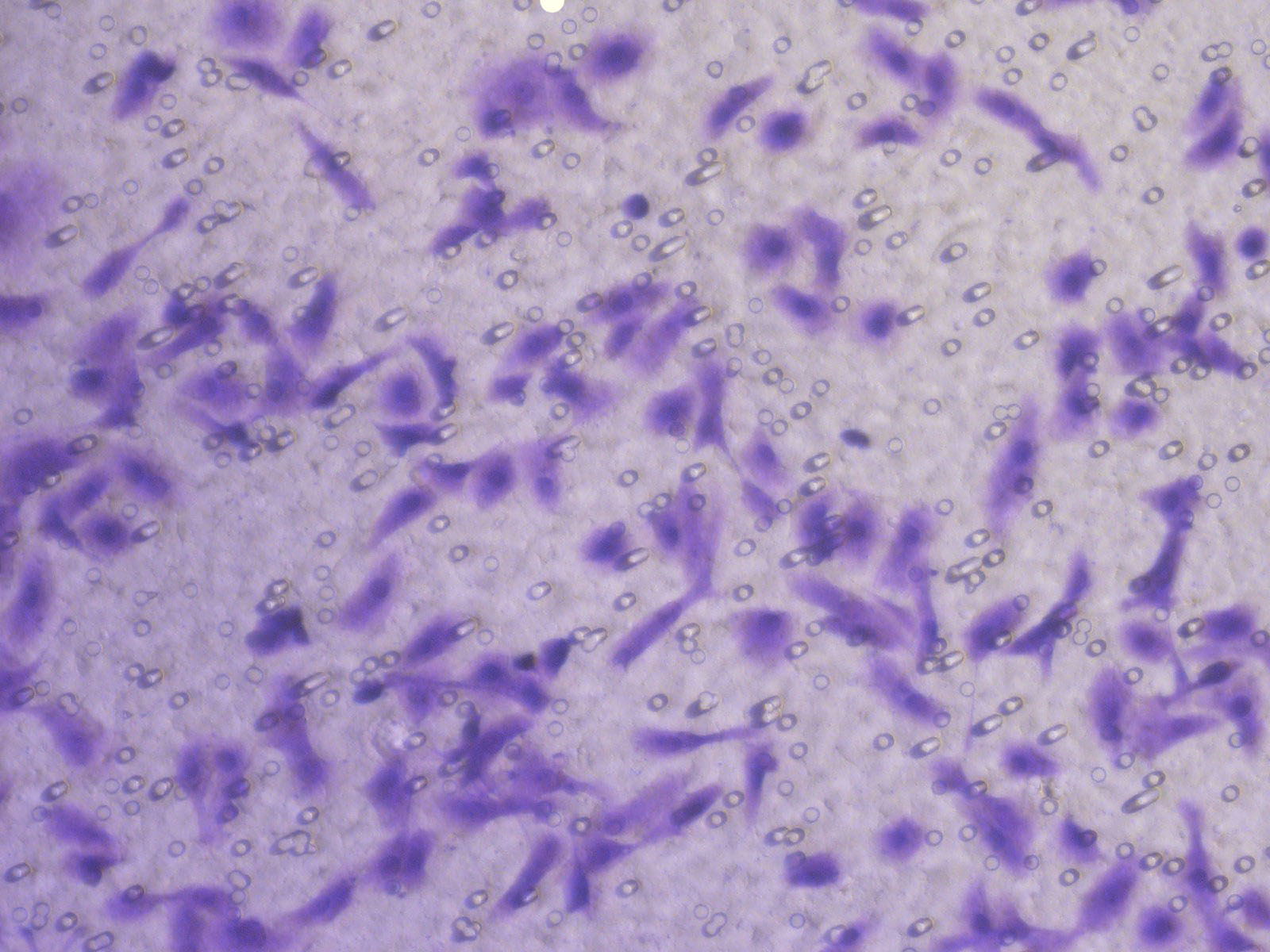

Supplement: Supplementary file 19 — Source Data [file 41467_2023_43282_MOESM19_ESM.zip › Source Data/Source data-Transwell raw images/Invasion/C ES-2/shNC-rep4.jpg]

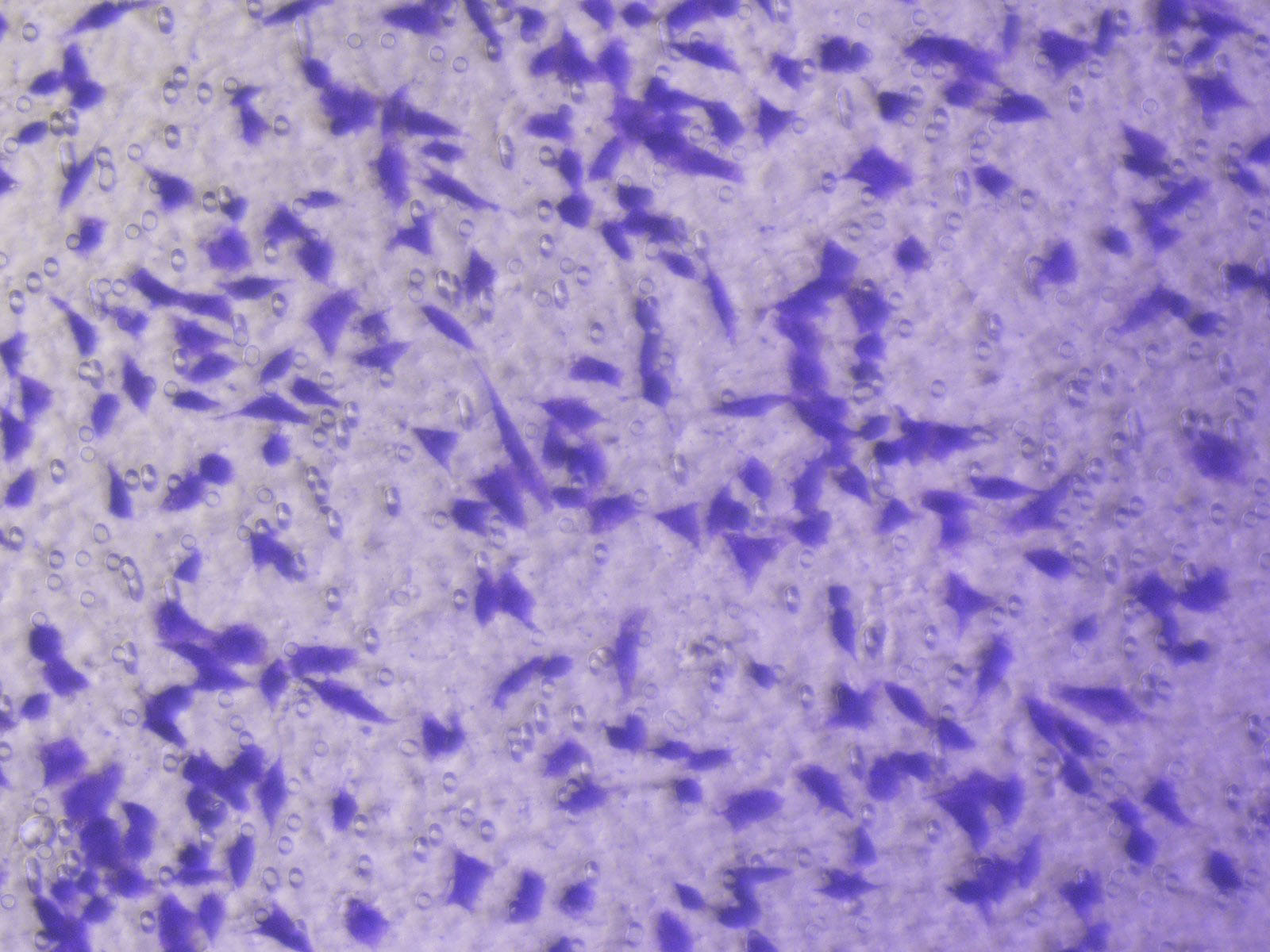

Supplement: Supplementary file 19 — Source Data [file 41467_2023_43282_MOESM19_ESM.zip › Source Data/Source data-Transwell raw images/Migration/A OVCAR-3/Control-rep1.jpg]

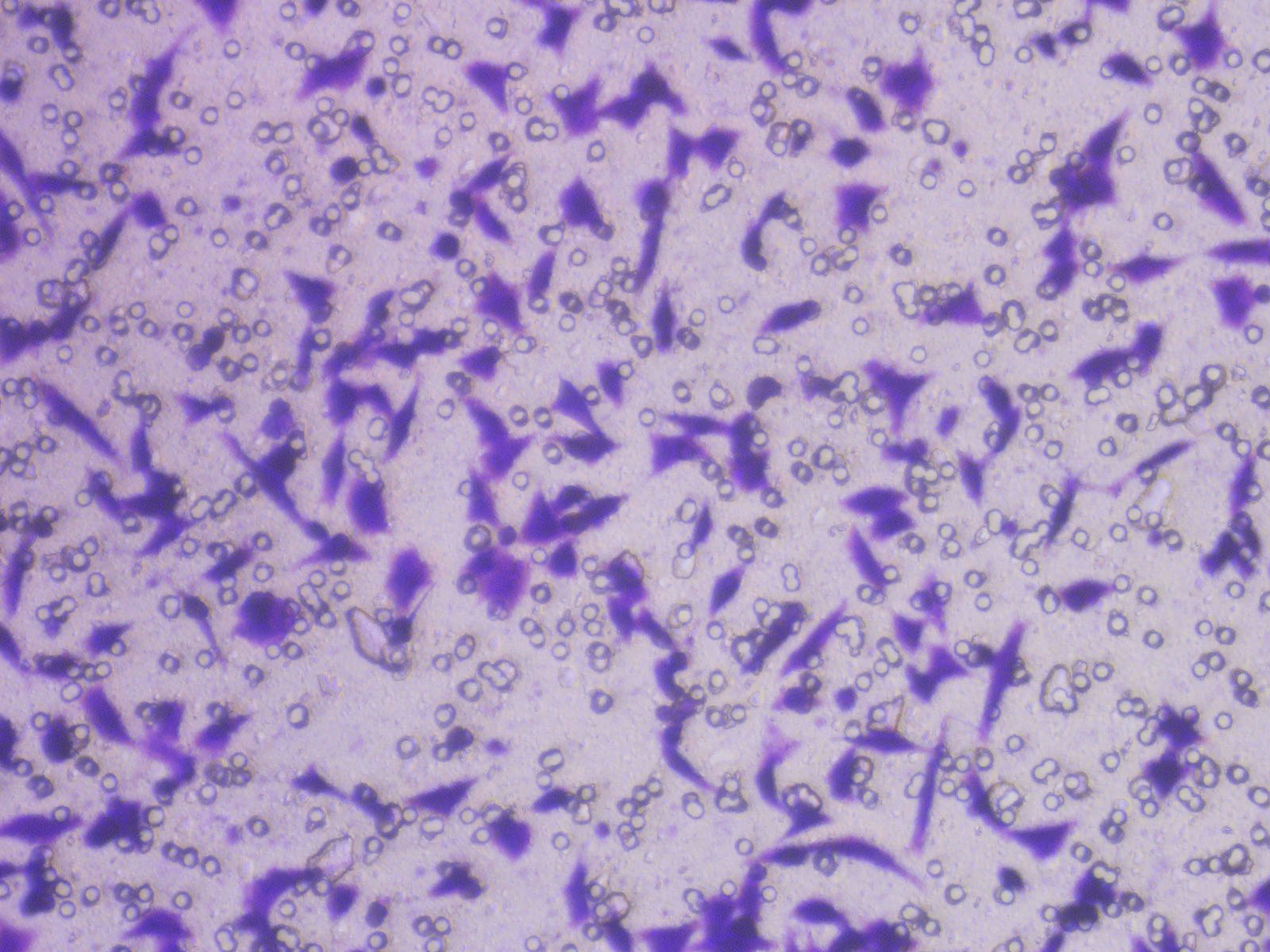

Supplement: Supplementary file 19 — Source Data [file 41467_2023_43282_MOESM19_ESM.zip › Source Data/Source data-Transwell raw images/Migration/A OVCAR-3/Control-rep2.jpg]

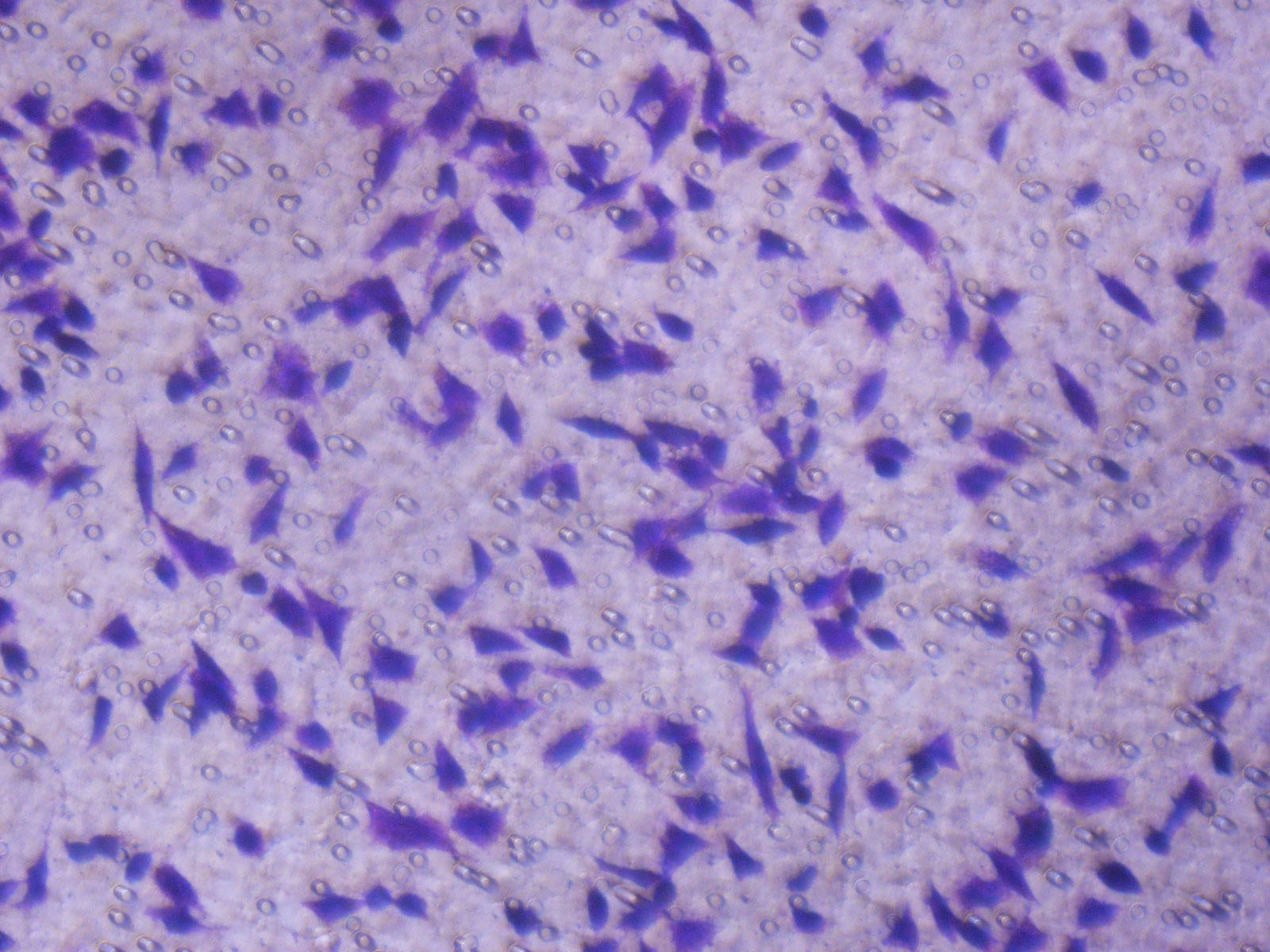

Supplement: Supplementary file 19 — Source Data [file 41467_2023_43282_MOESM19_ESM.zip › Source Data/Source data-Transwell raw images/Migration/A OVCAR-3/Control-rep3.jpg]

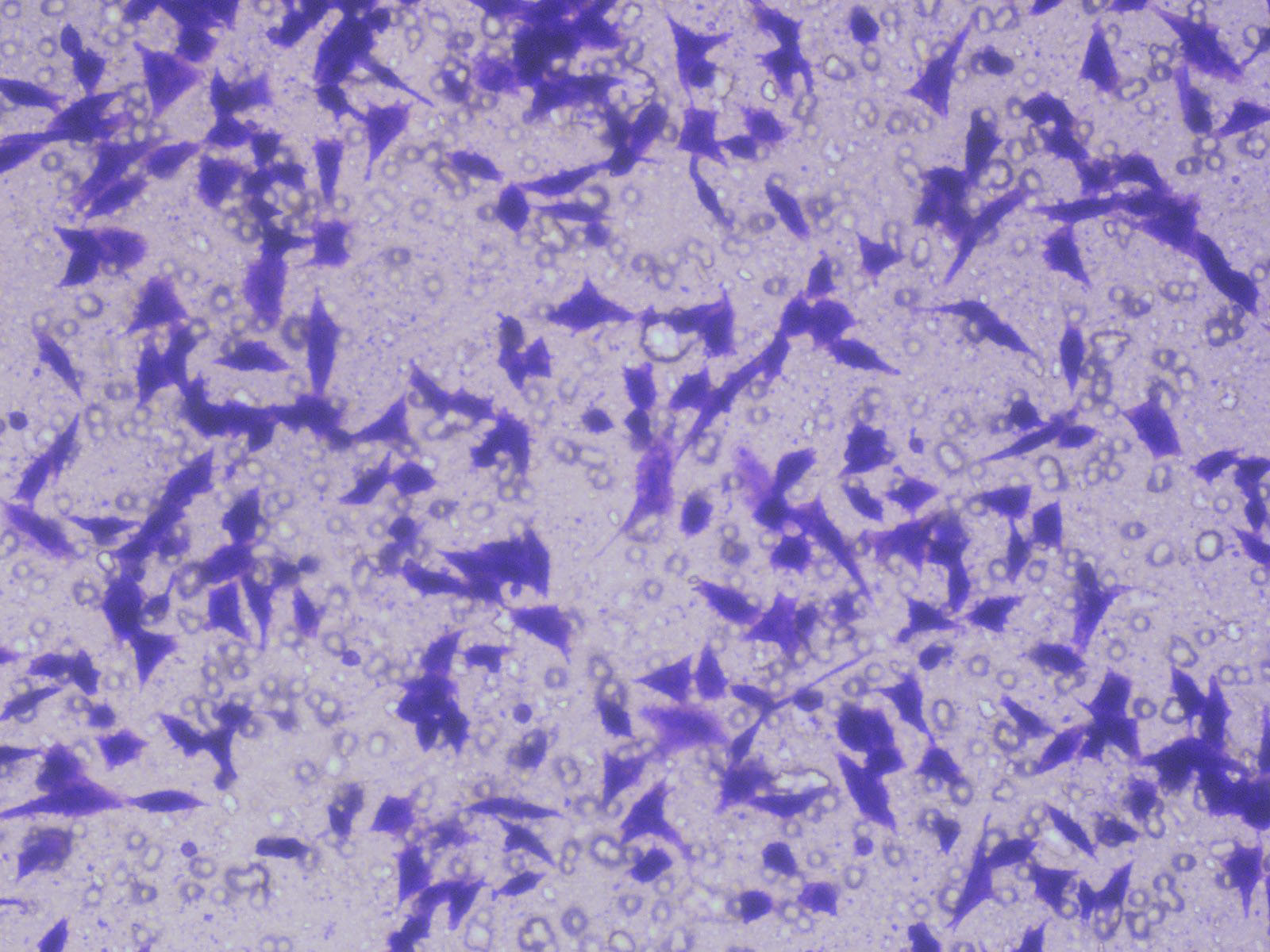

Supplement: Supplementary file 19 — Source Data [file 41467_2023_43282_MOESM19_ESM.zip › Source Data/Source data-Transwell raw images/Migration/A OVCAR-3/Control-rep4.jpg]

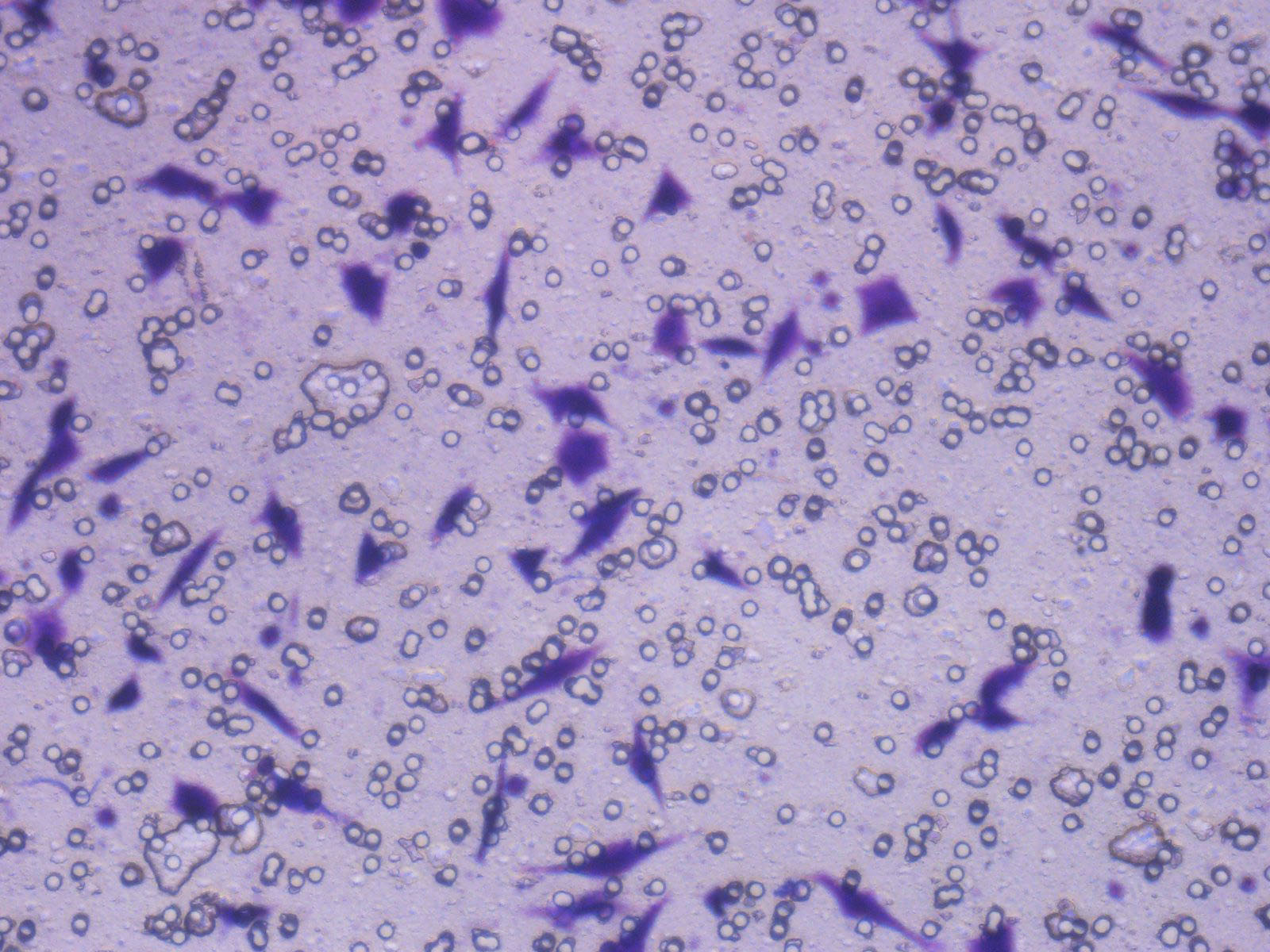

Supplement: Supplementary file 19 — Source Data [file 41467_2023_43282_MOESM19_ESM.zip › Source Data/Source data-Transwell raw images/Migration/A OVCAR-3/shMPP7-rep1.jpg]

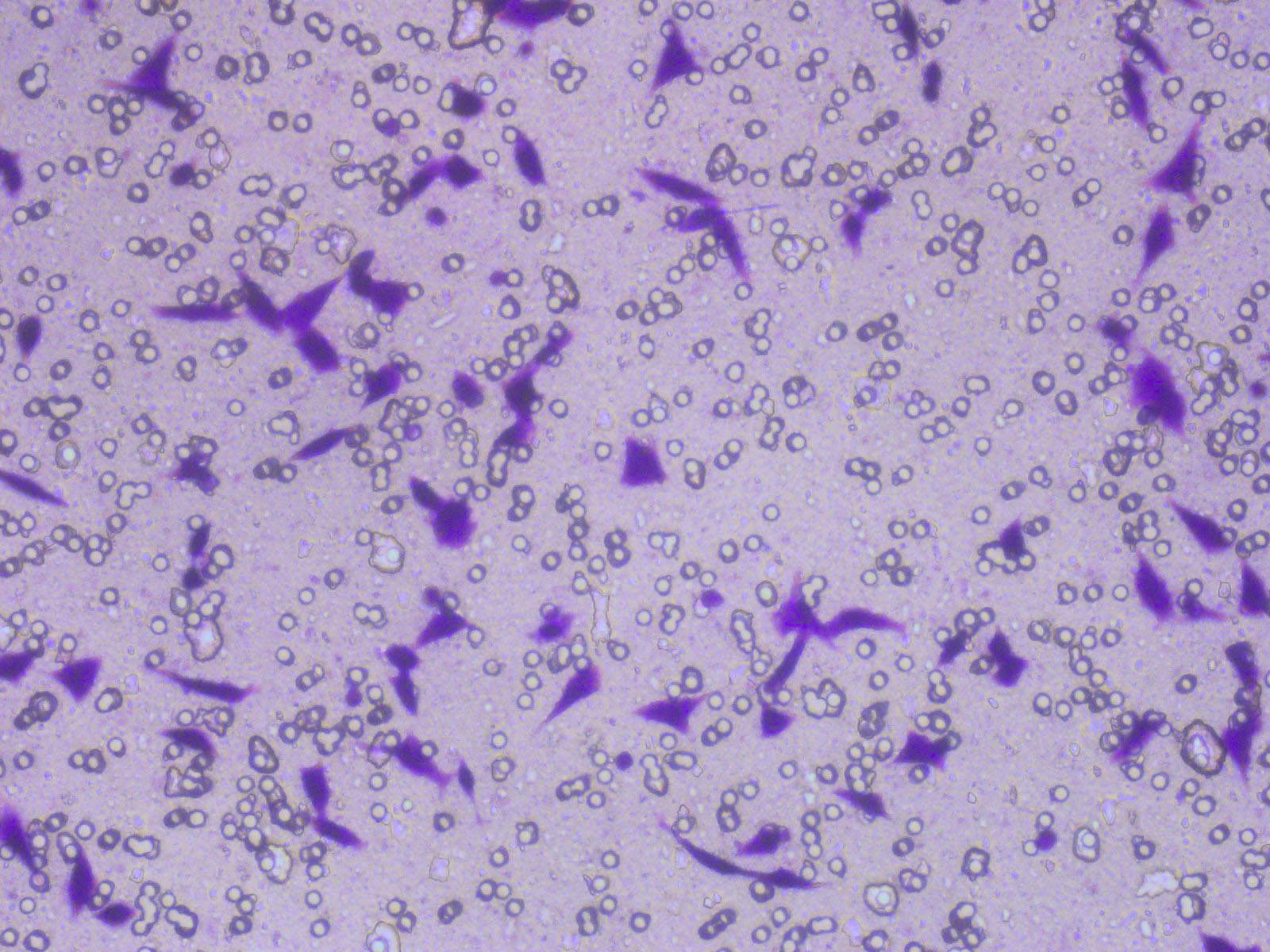

Supplement: Supplementary file 19 — Source Data [file 41467_2023_43282_MOESM19_ESM.zip › Source Data/Source data-Transwell raw images/Migration/A OVCAR-3/shMPP7-rep2.jpg]

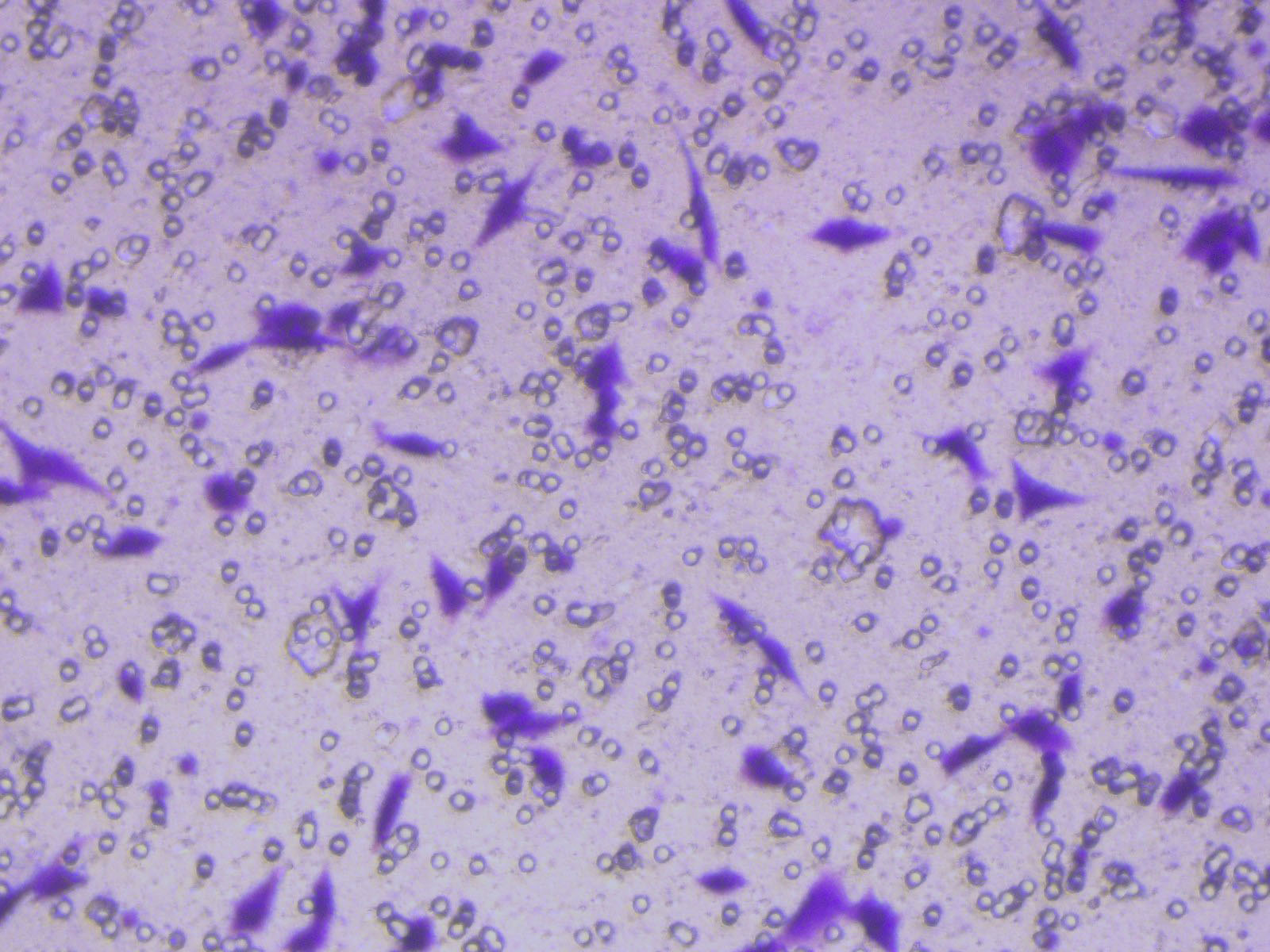

Supplement: Supplementary file 19 — Source Data [file 41467_2023_43282_MOESM19_ESM.zip › Source Data/Source data-Transwell raw images/Migration/A OVCAR-3/shMPP7-rep3.jpg]

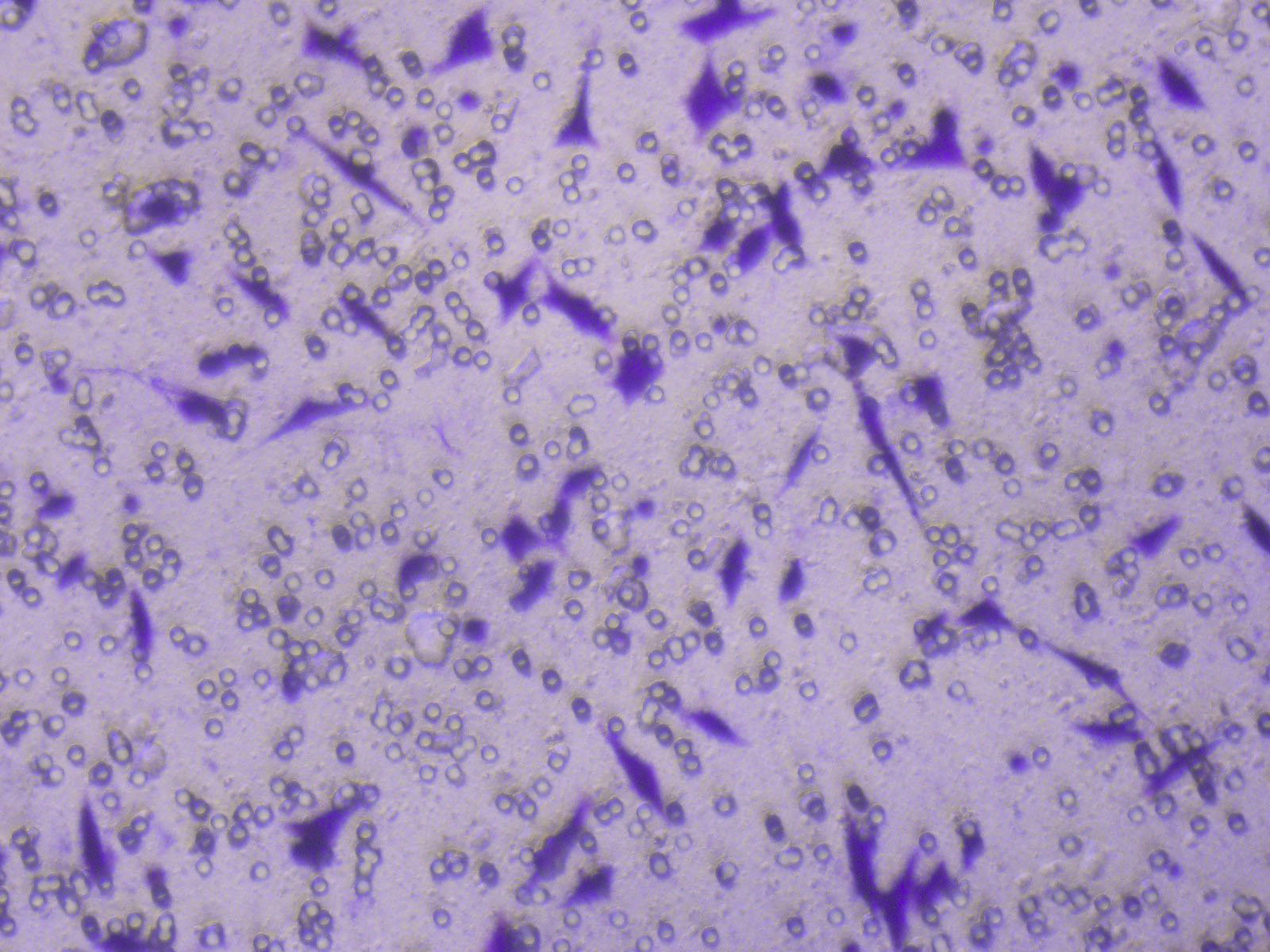

Supplement: Supplementary file 19 — Source Data [file 41467_2023_43282_MOESM19_ESM.zip › Source Data/Source data-Transwell raw images/Migration/A OVCAR-3/shMPP7-rep4.jpg]

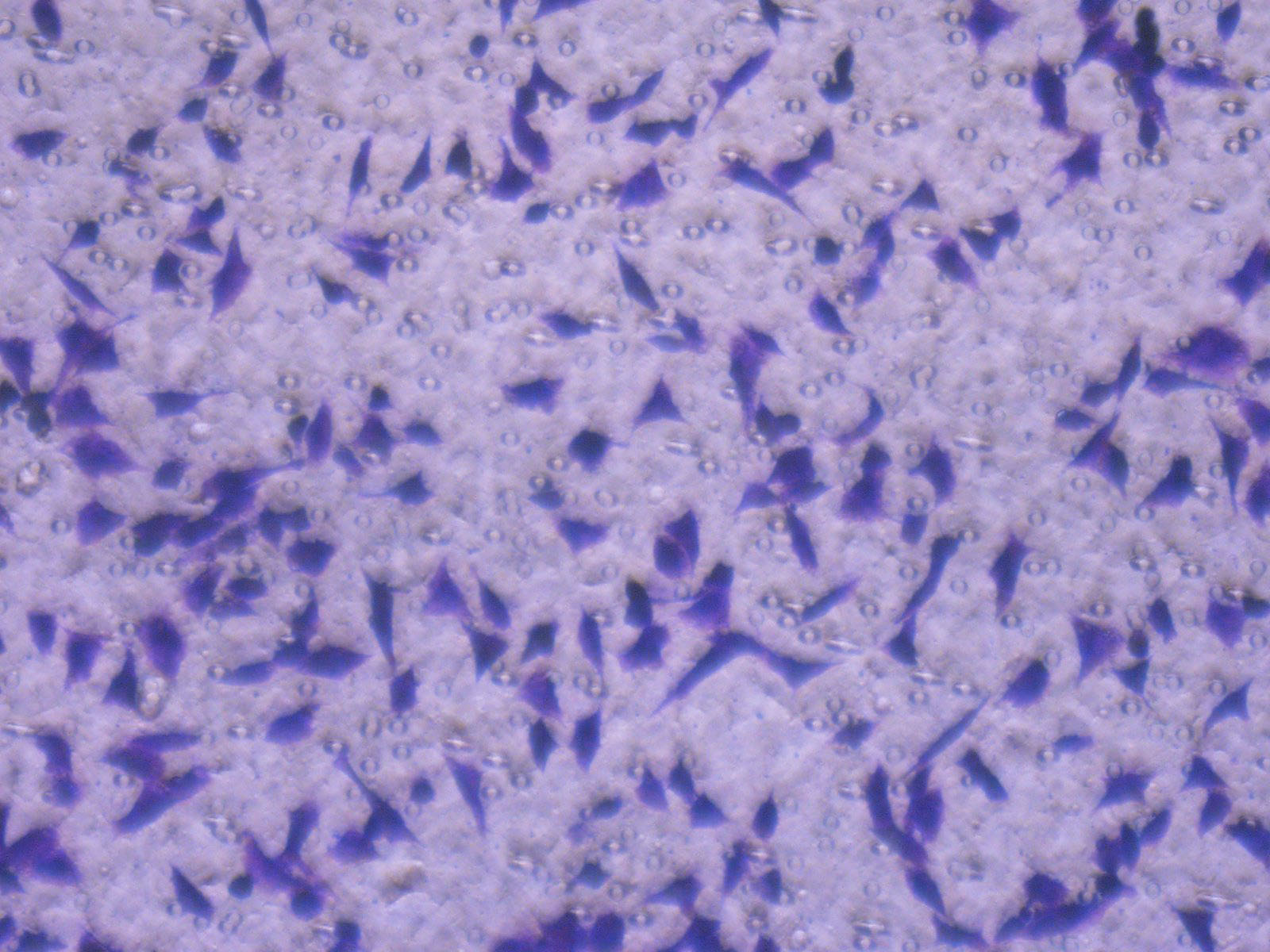

Supplement: Supplementary file 19 — Source Data [file 41467_2023_43282_MOESM19_ESM.zip › Source Data/Source data-Transwell raw images/Migration/A OVCAR-3/shNC-rep1.jpg]

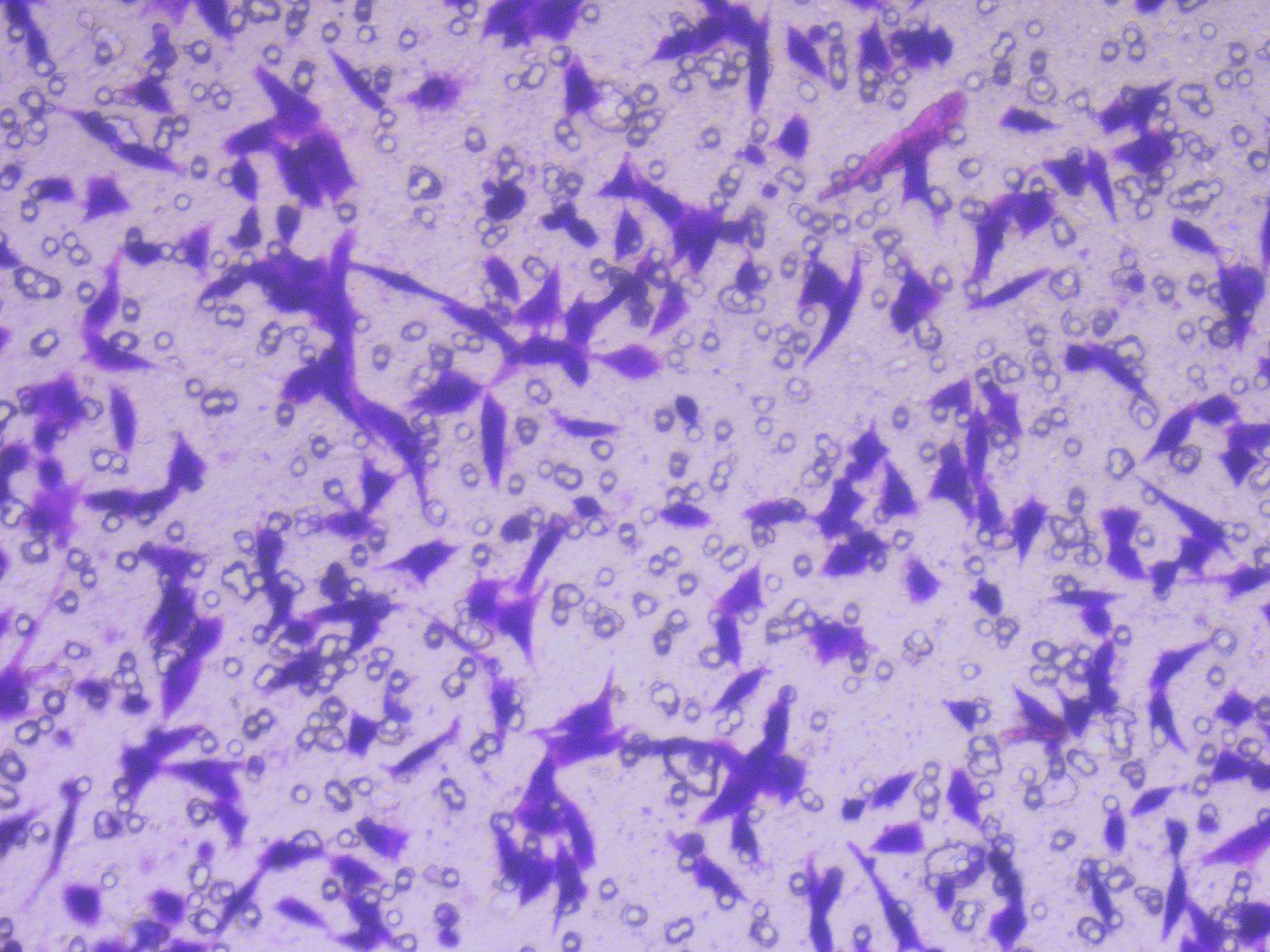

Supplement: Supplementary file 19 — Source Data [file 41467_2023_43282_MOESM19_ESM.zip › Source Data/Source data-Transwell raw images/Migration/A OVCAR-3/shNC-rep2.jpg]

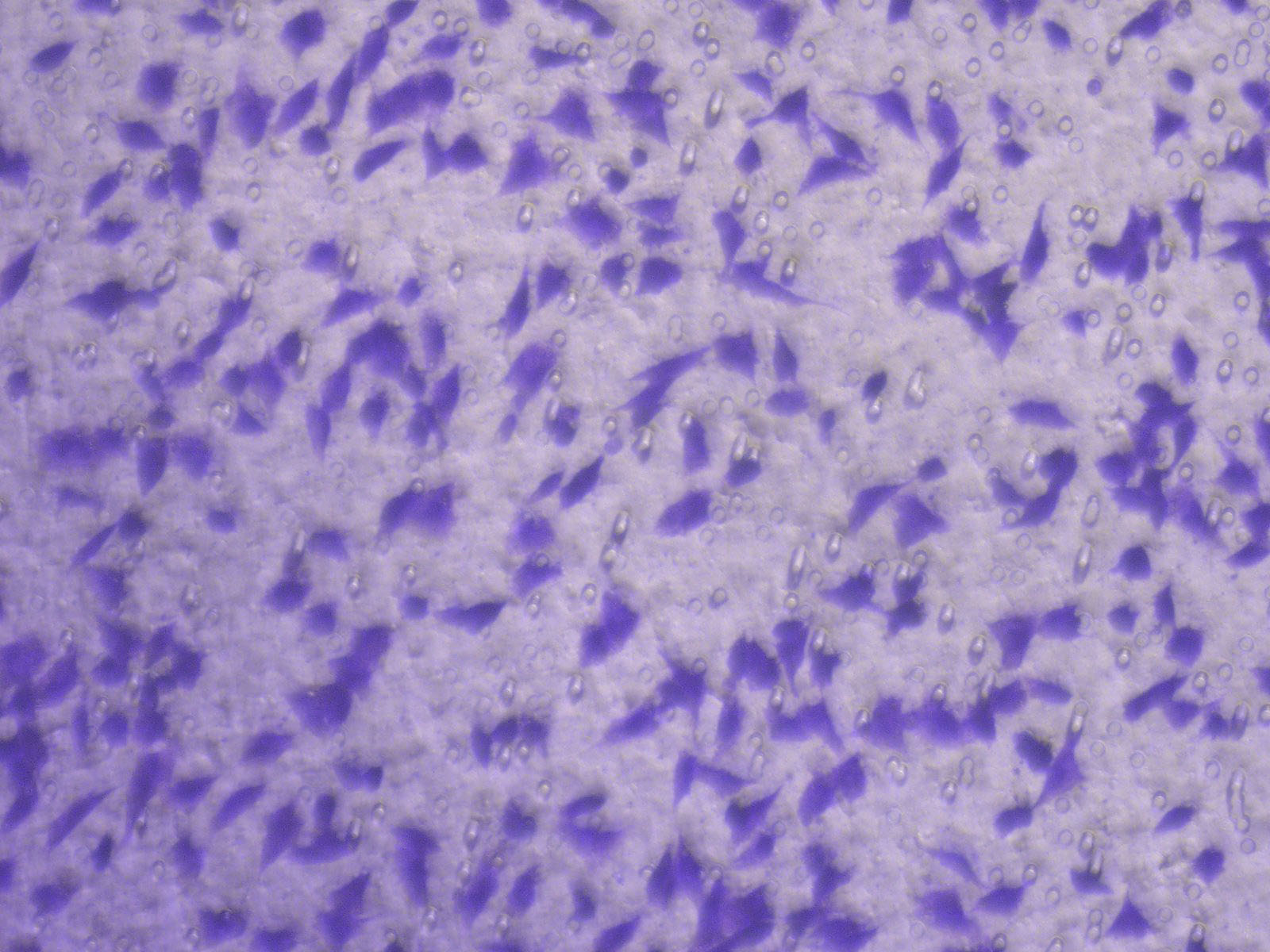

Supplement: Supplementary file 19 — Source Data [file 41467_2023_43282_MOESM19_ESM.zip › Source Data/Source data-Transwell raw images/Migration/A OVCAR-3/shNC-rep3.jpg]

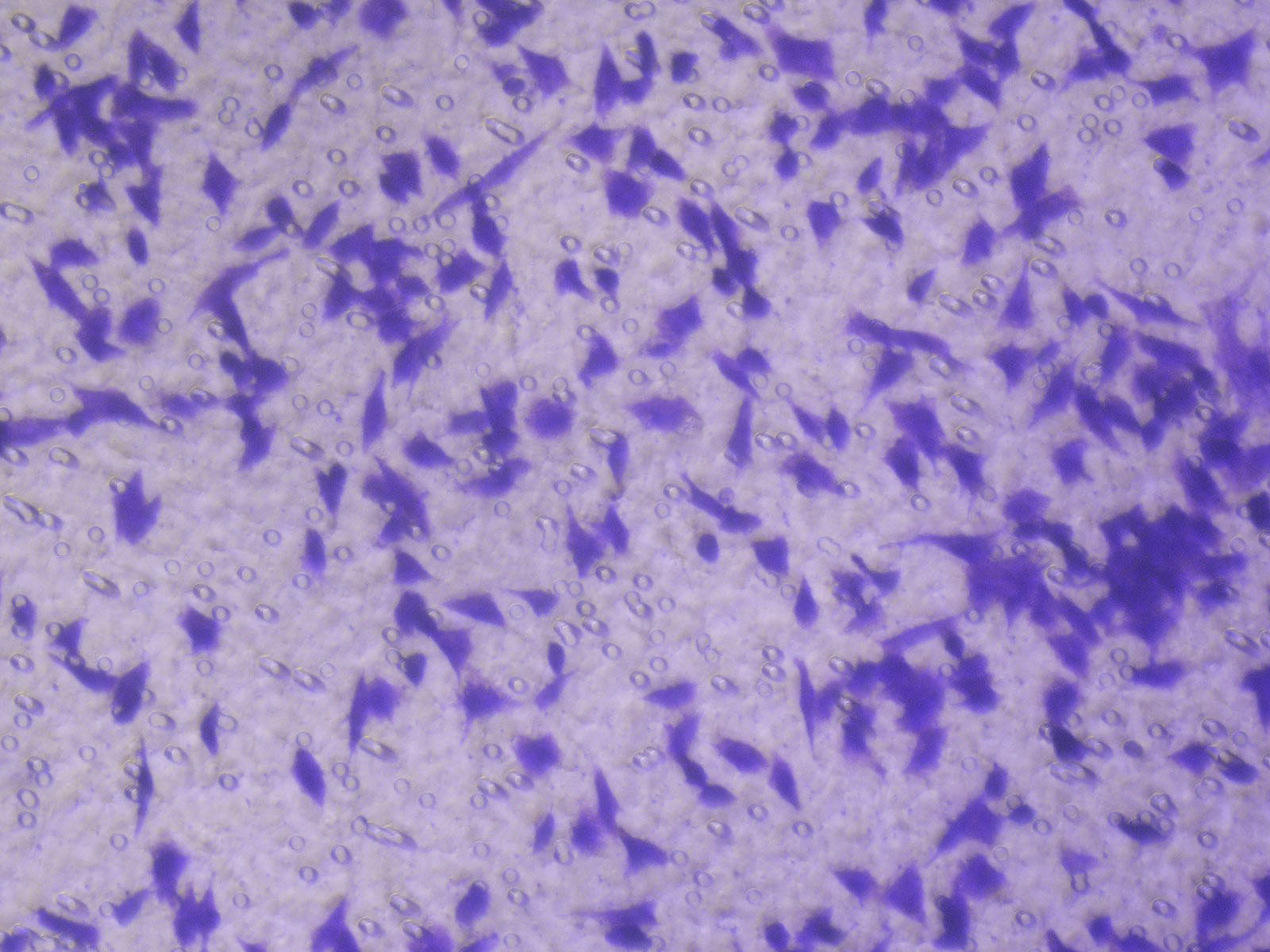

Supplement: Supplementary file 19 — Source Data [file 41467_2023_43282_MOESM19_ESM.zip › Source Data/Source data-Transwell raw images/Migration/A OVCAR-3/shNC-rep4.jpg]

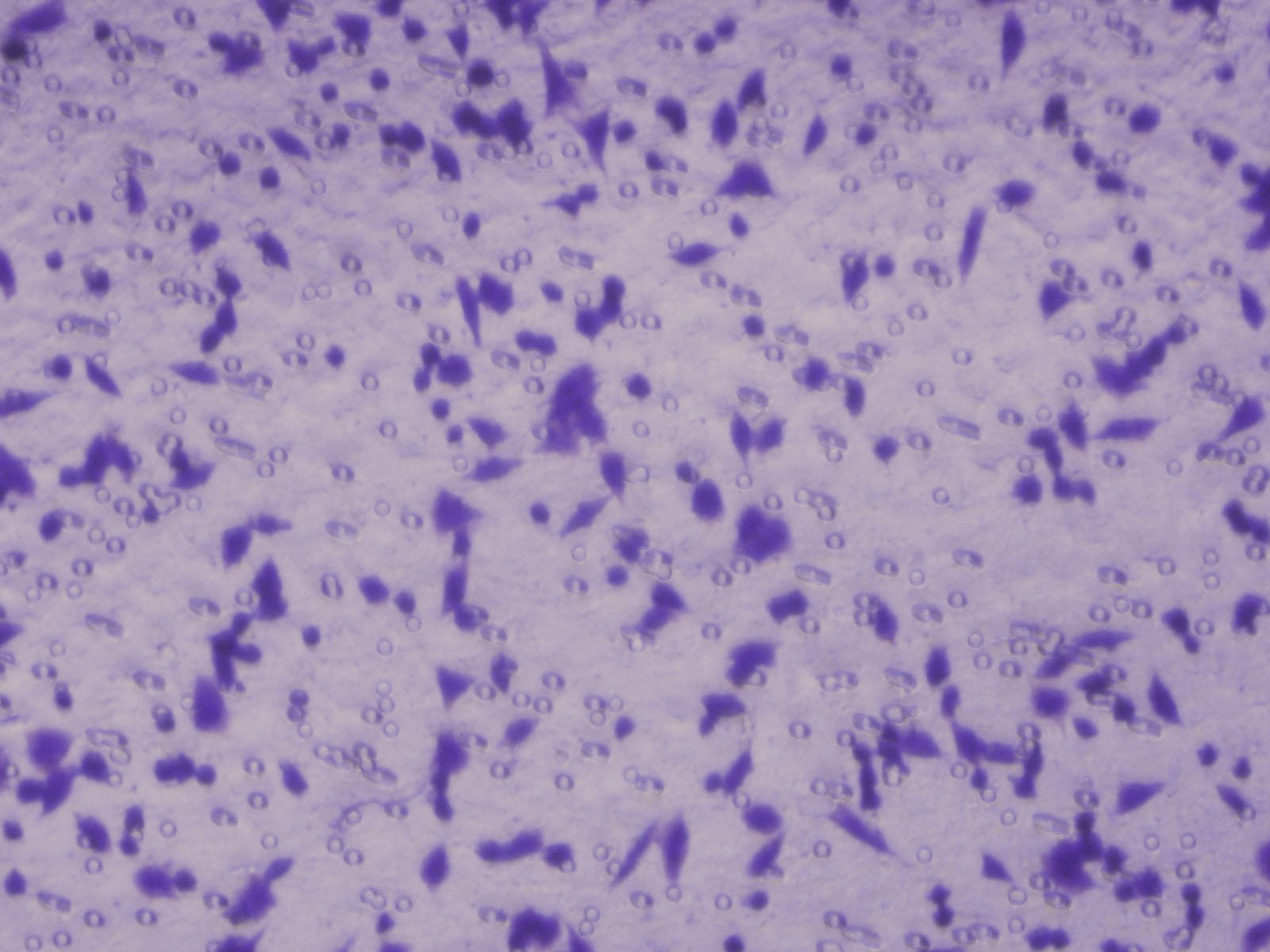

Supplement: Supplementary file 19 — Source Data [file 41467_2023_43282_MOESM19_ESM.zip › Source Data/Source data-Transwell raw images/Migration/B A2780/Control-rep1.jpg]

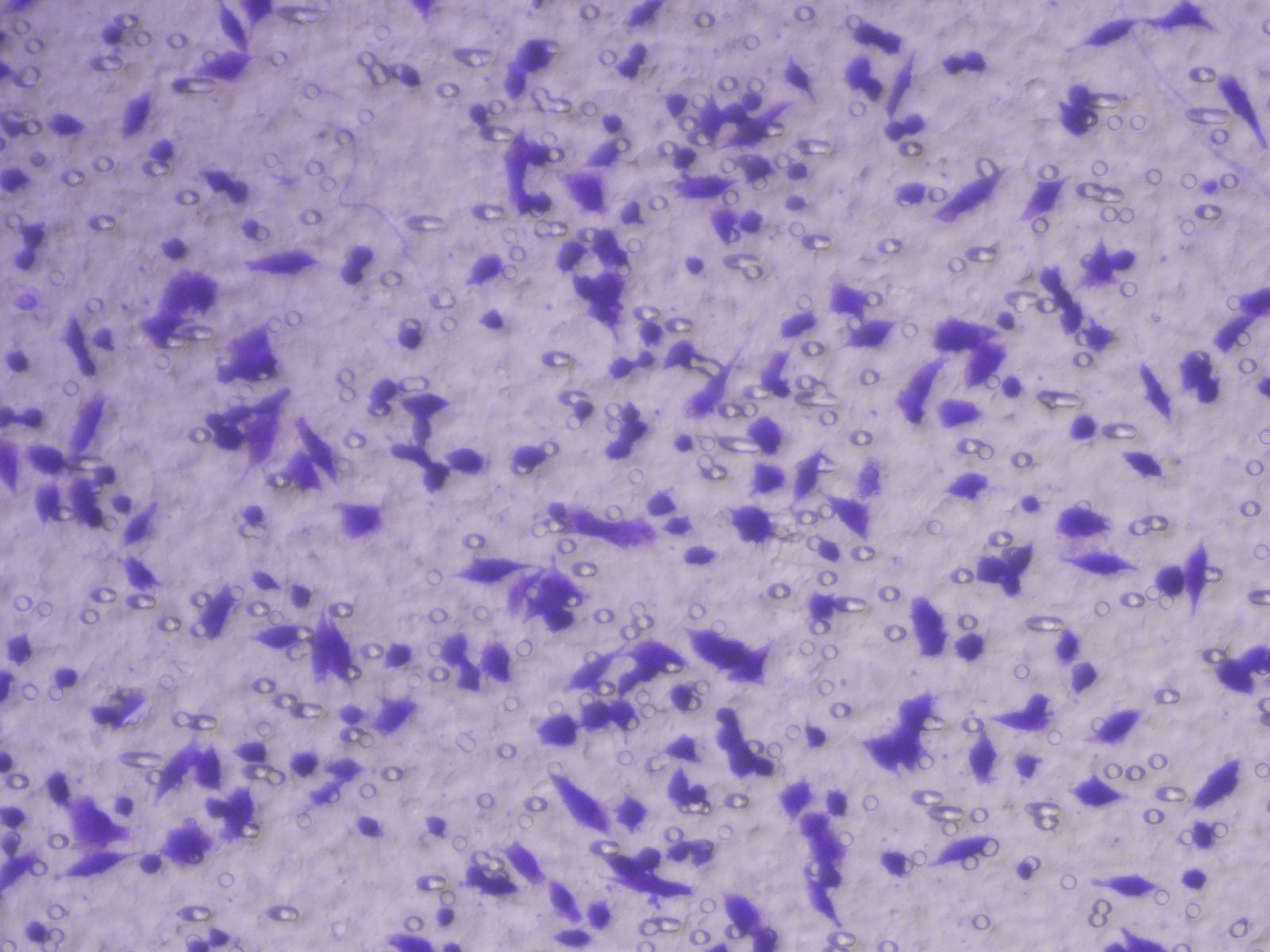

Supplement: Supplementary file 19 — Source Data [file 41467_2023_43282_MOESM19_ESM.zip › Source Data/Source data-Transwell raw images/Migration/B A2780/Control-rep2.jpg]

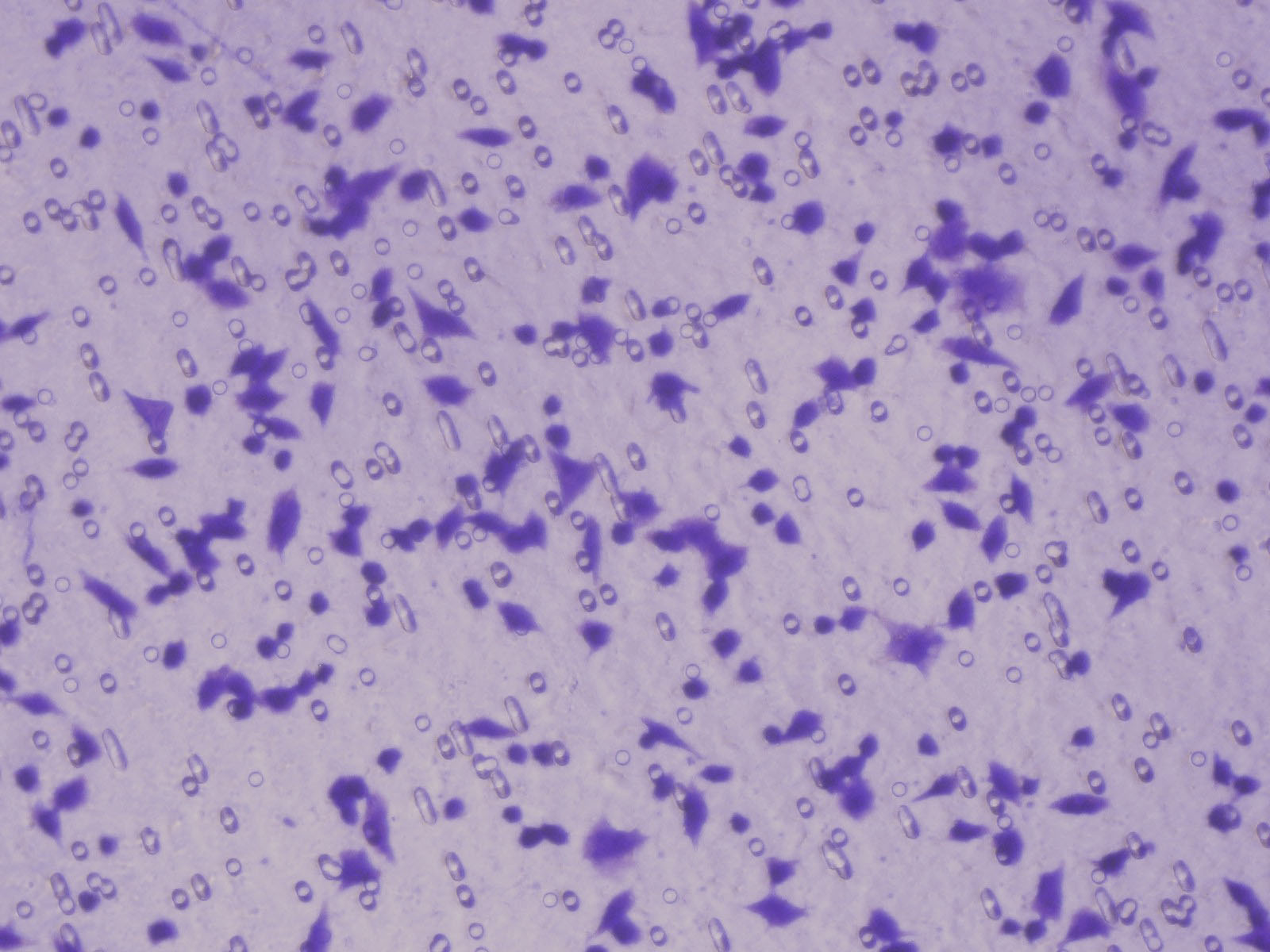

Supplement: Supplementary file 19 — Source Data [file 41467_2023_43282_MOESM19_ESM.zip › Source Data/Source data-Transwell raw images/Migration/B A2780/Control-rep3.jpg]

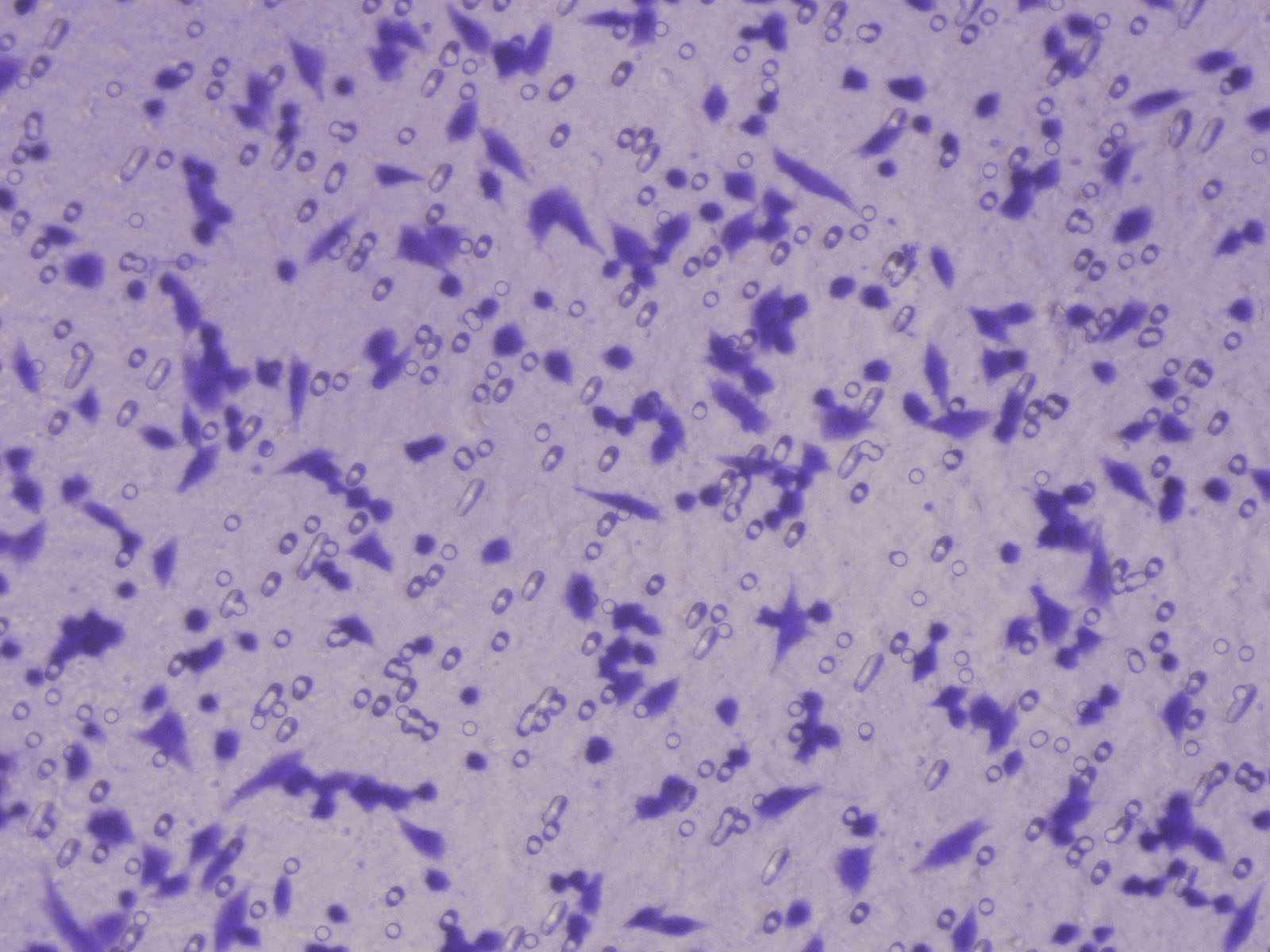

Supplement: Supplementary file 19 — Source Data [file 41467_2023_43282_MOESM19_ESM.zip › Source Data/Source data-Transwell raw images/Migration/B A2780/Control-rep4.jpg]

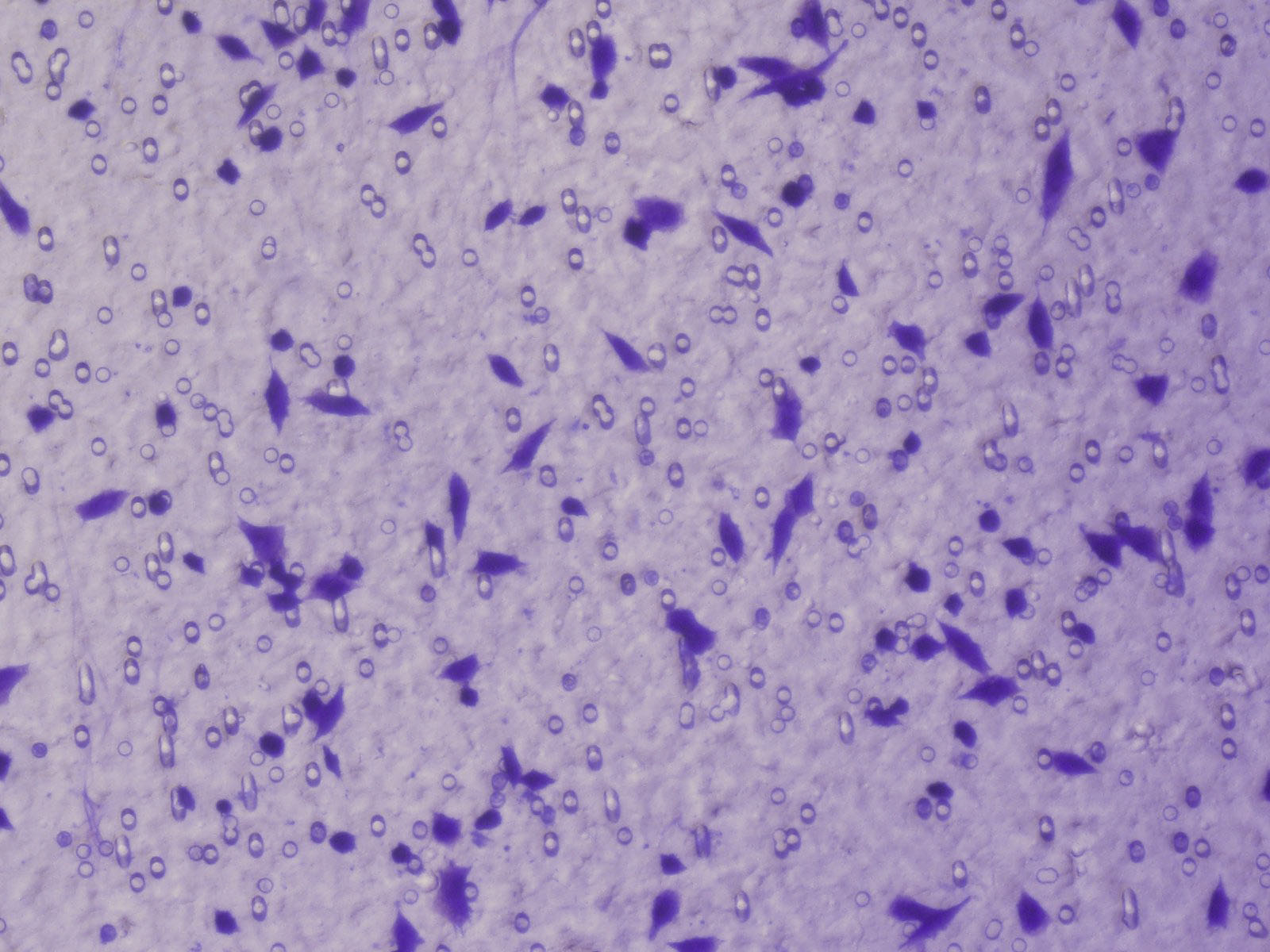

Supplement: Supplementary file 19 — Source Data [file 41467_2023_43282_MOESM19_ESM.zip › Source Data/Source data-Transwell raw images/Migration/B A2780/shMPP7-rep1.jpg]

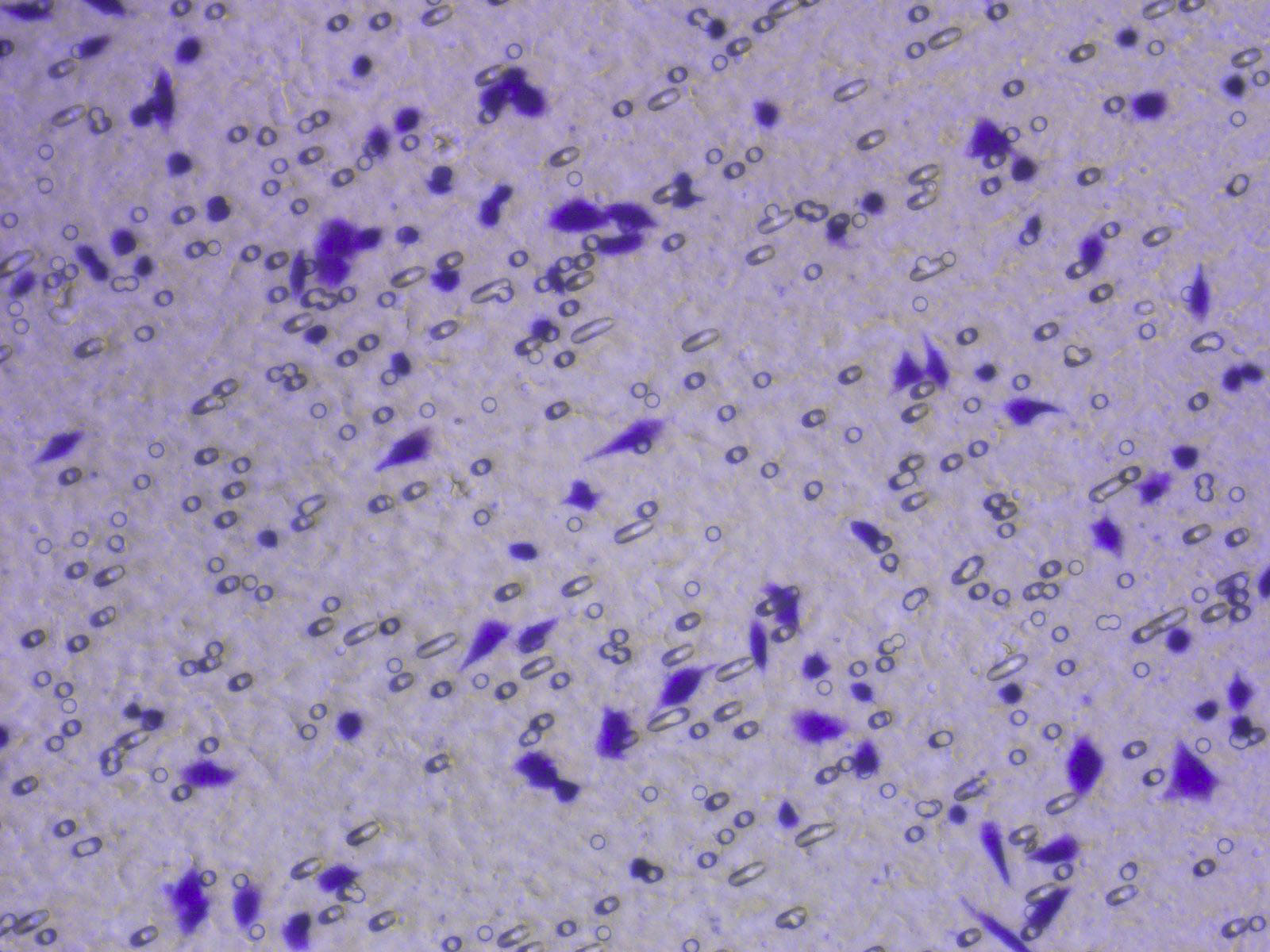

Supplement: Supplementary file 19 — Source Data [file 41467_2023_43282_MOESM19_ESM.zip › Source Data/Source data-Transwell raw images/Migration/B A2780/shMPP7-rep2.jpg]

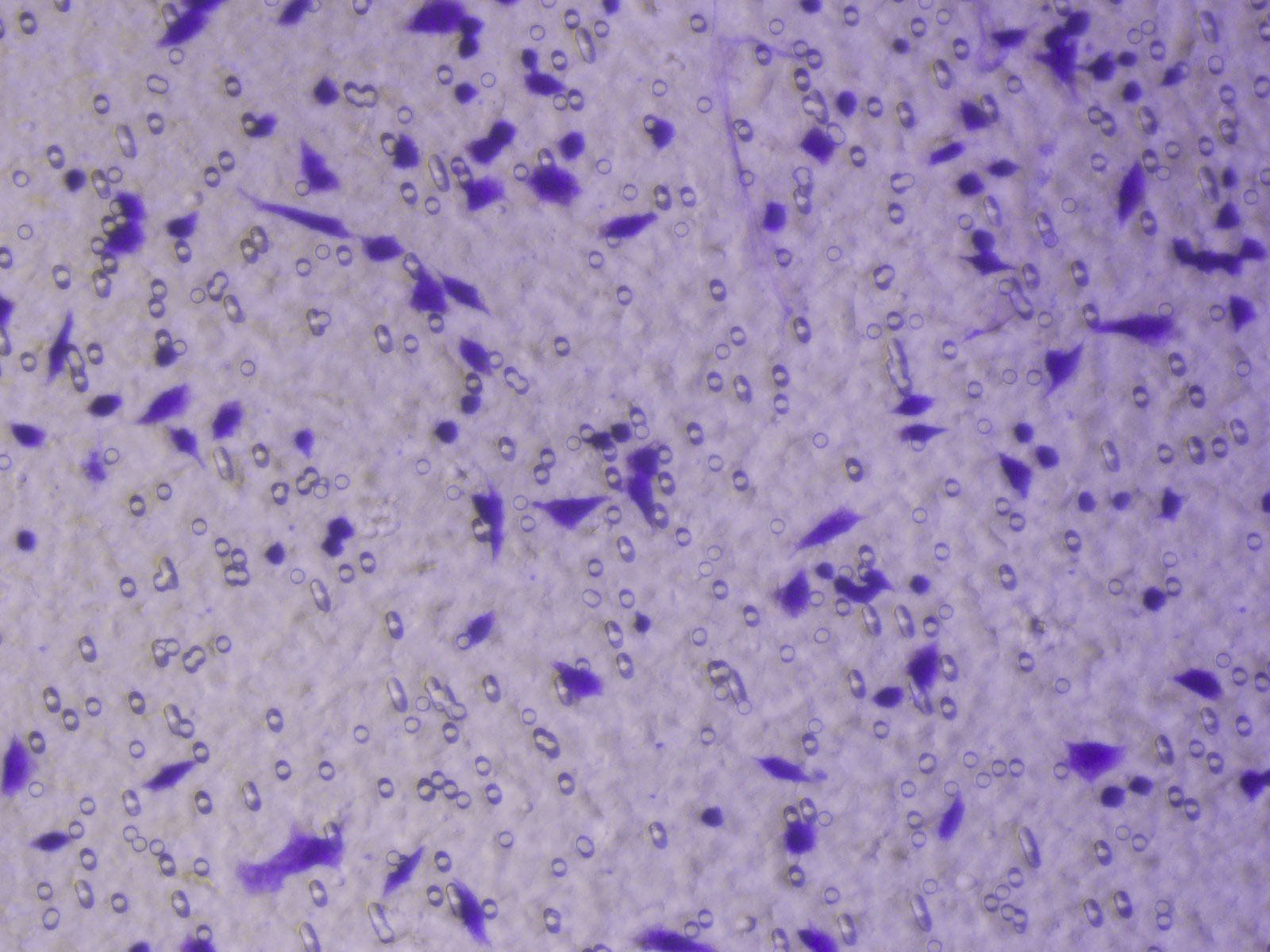

Supplement: Supplementary file 19 — Source Data [file 41467_2023_43282_MOESM19_ESM.zip › Source Data/Source data-Transwell raw images/Migration/B A2780/shMPP7-rep3.jpg]

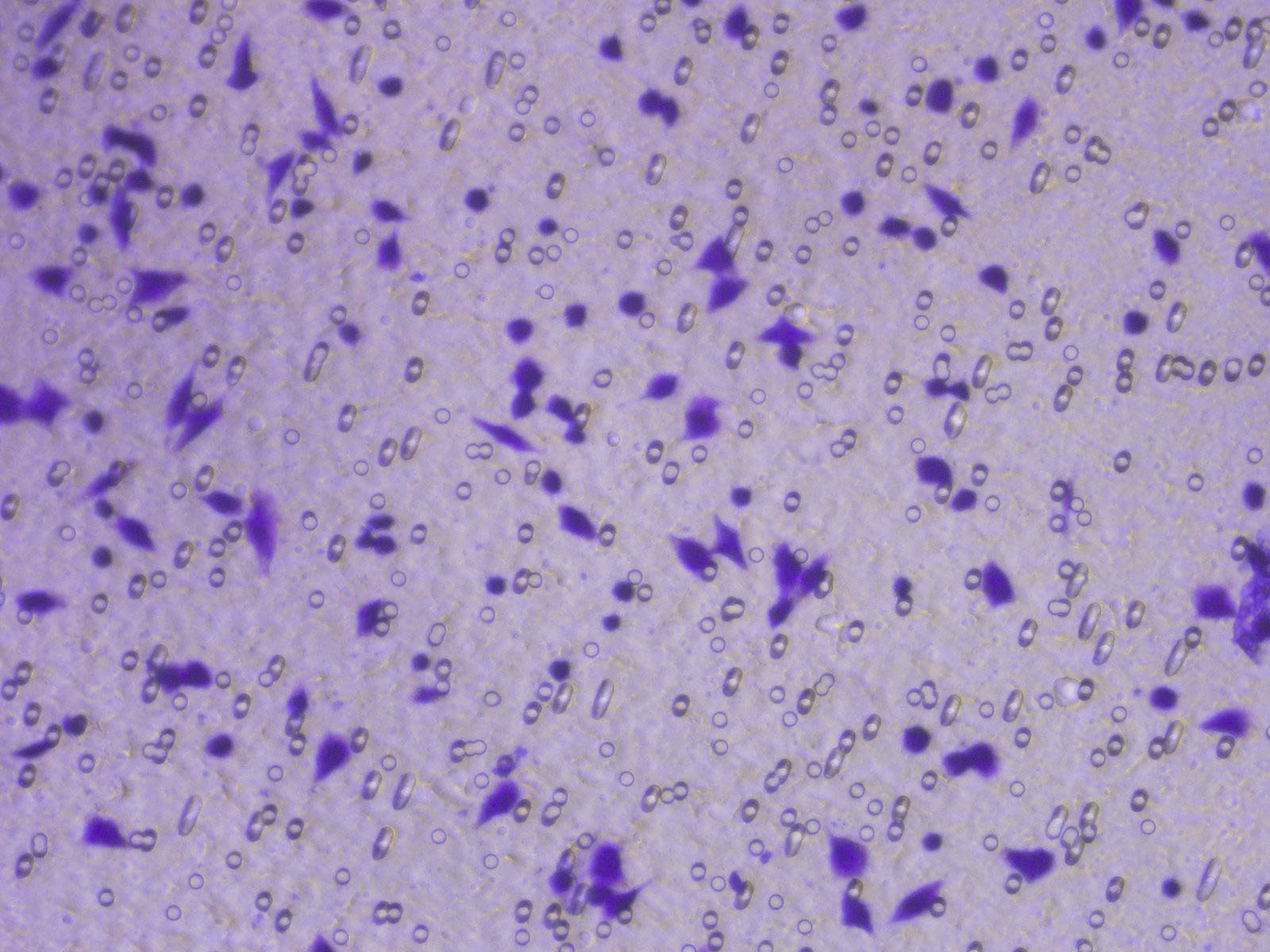

Supplement: Supplementary file 19 — Source Data [file 41467_2023_43282_MOESM19_ESM.zip › Source Data/Source data-Transwell raw images/Migration/B A2780/shMPP7-rep4.jpg]

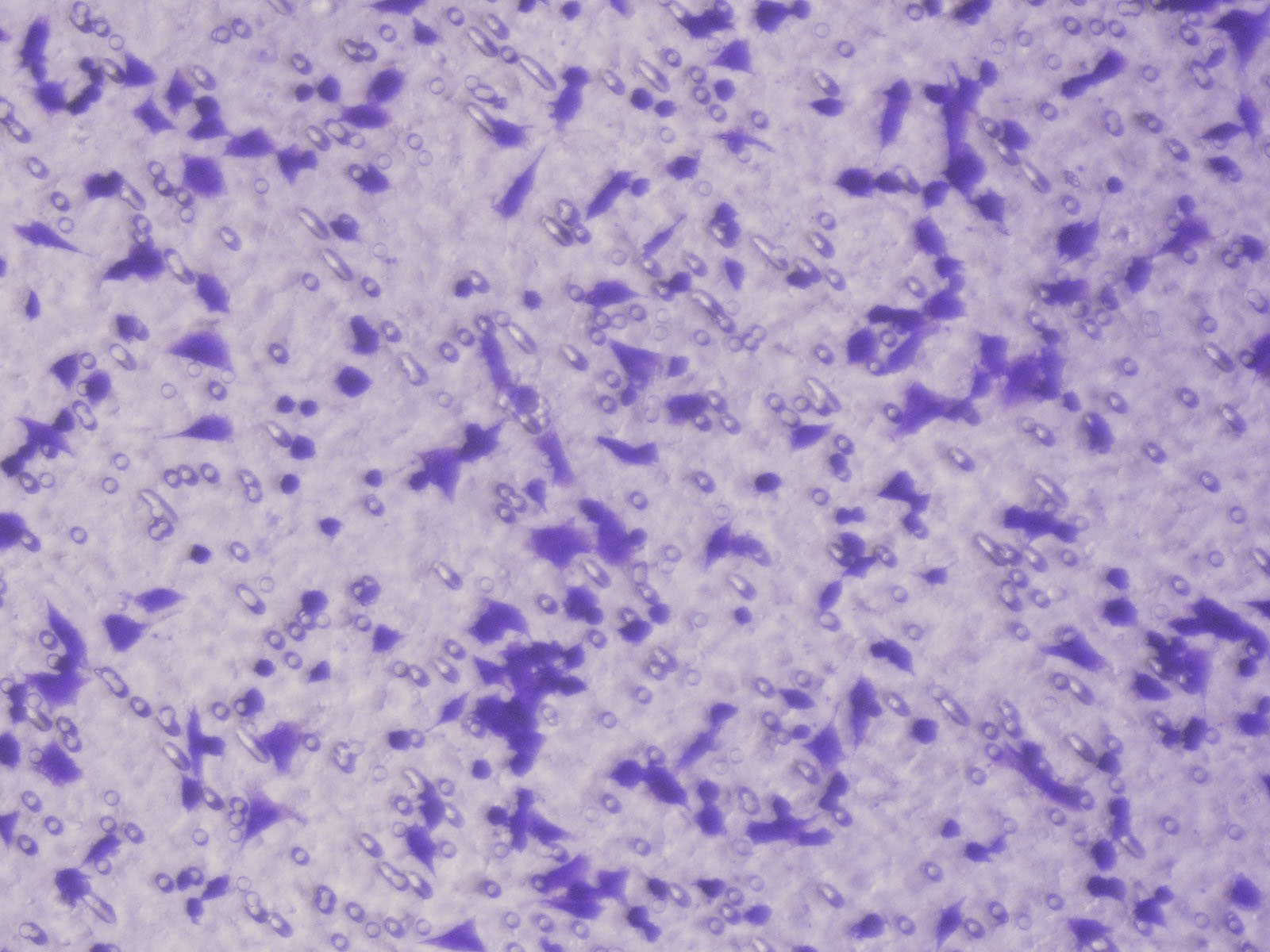

Supplement: Supplementary file 19 — Source Data [file 41467_2023_43282_MOESM19_ESM.zip › Source Data/Source data-Transwell raw images/Migration/B A2780/shNC-rep1.jpg]

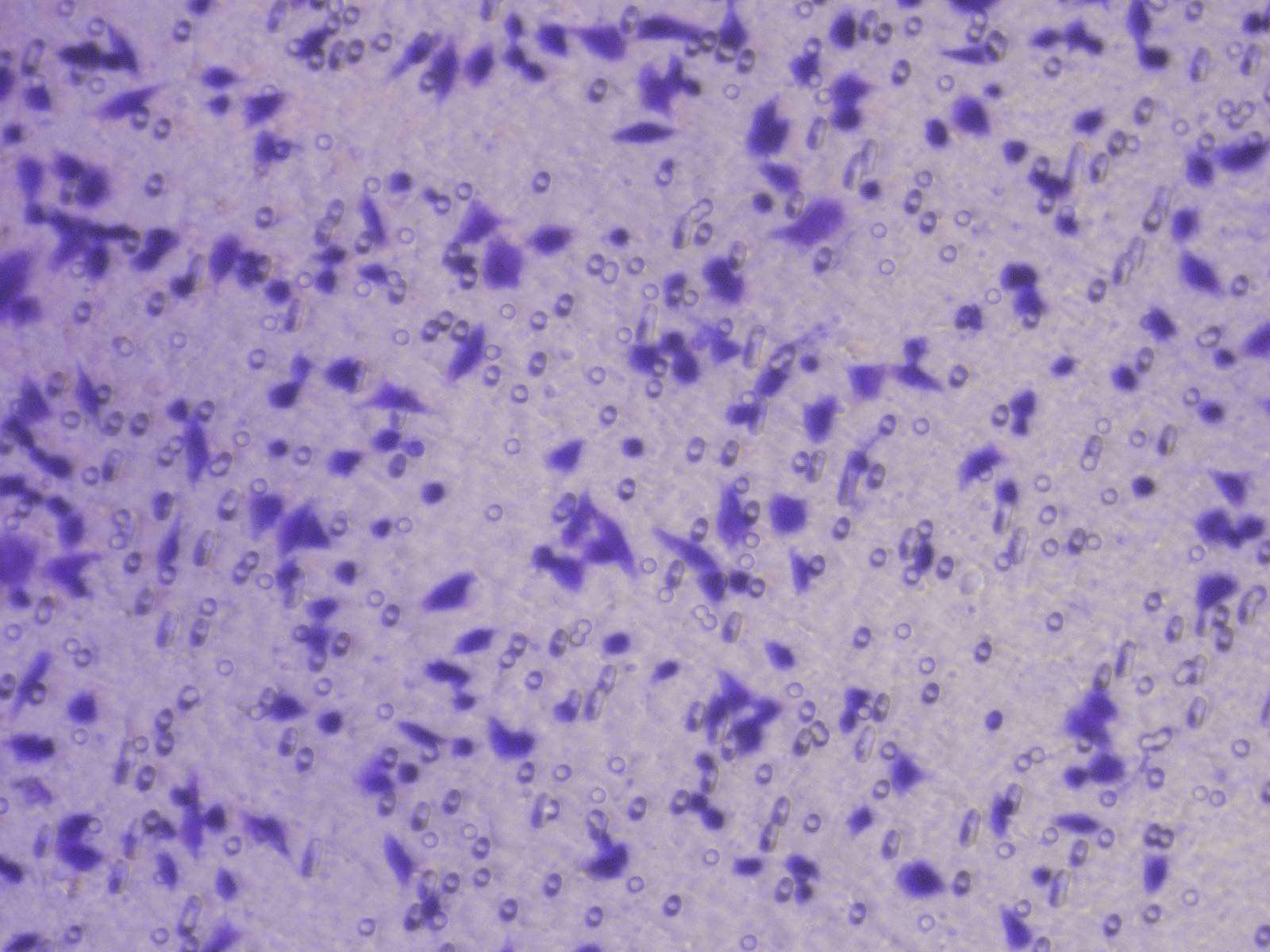

Supplement: Supplementary file 19 — Source Data [file 41467_2023_43282_MOESM19_ESM.zip › Source Data/Source data-Transwell raw images/Migration/B A2780/shNC-rep2.jpg]

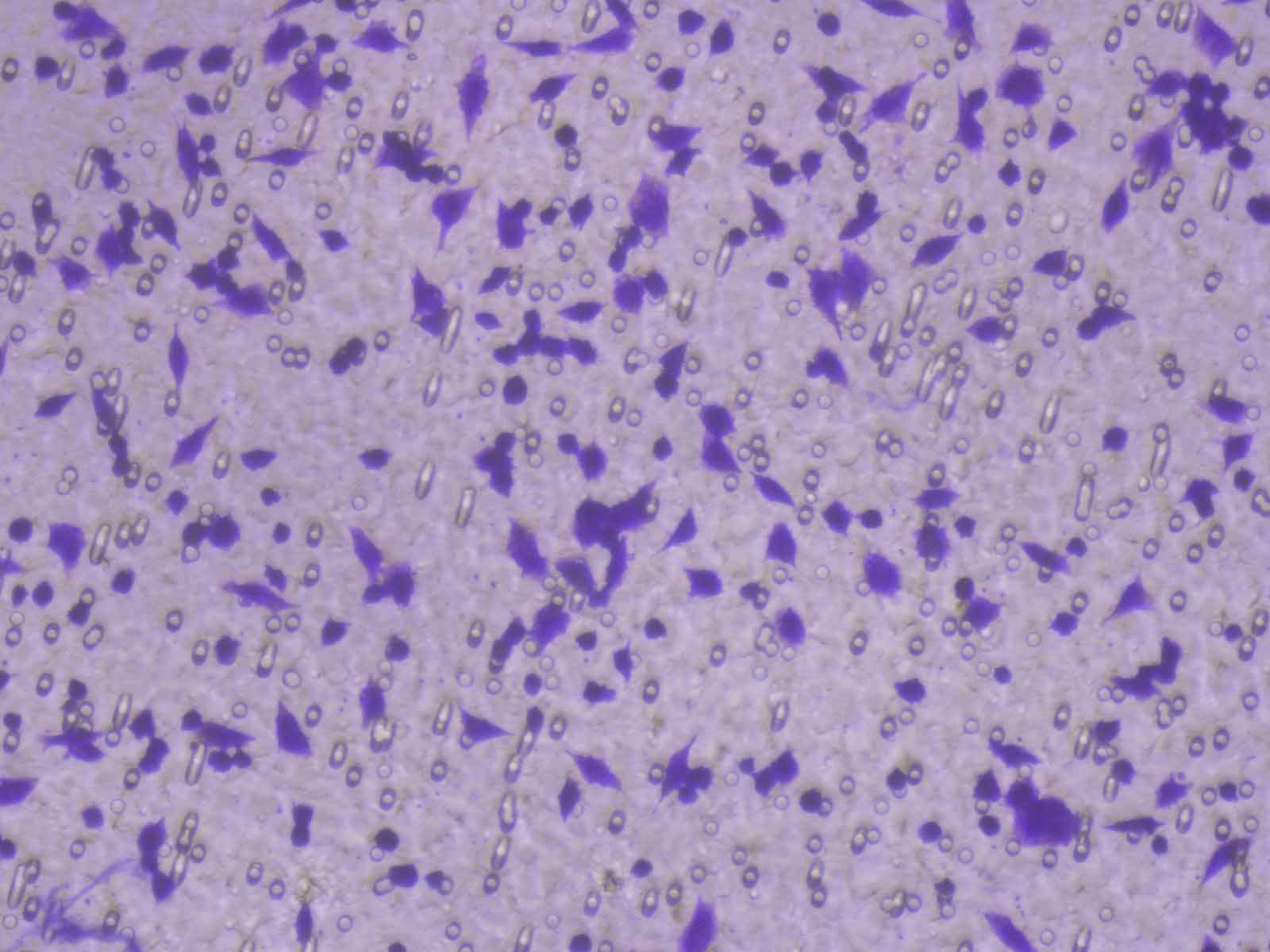

Supplement: Supplementary file 19 — Source Data [file 41467_2023_43282_MOESM19_ESM.zip › Source Data/Source data-Transwell raw images/Migration/B A2780/shNC-rep3.jpg]

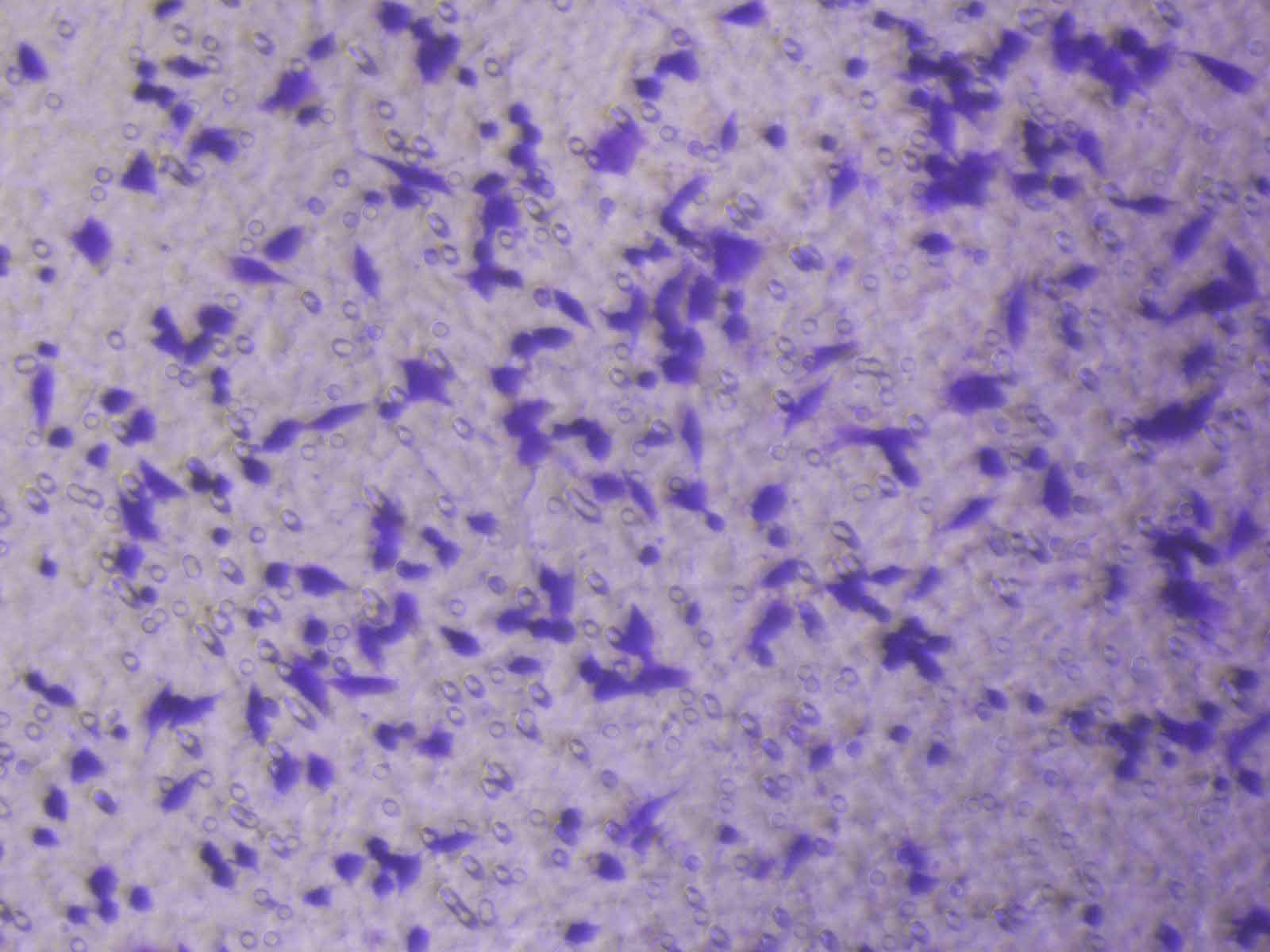

Supplement: Supplementary file 19 — Source Data [file 41467_2023_43282_MOESM19_ESM.zip › Source Data/Source data-Transwell raw images/Migration/B A2780/shNC-rep4.jpg]

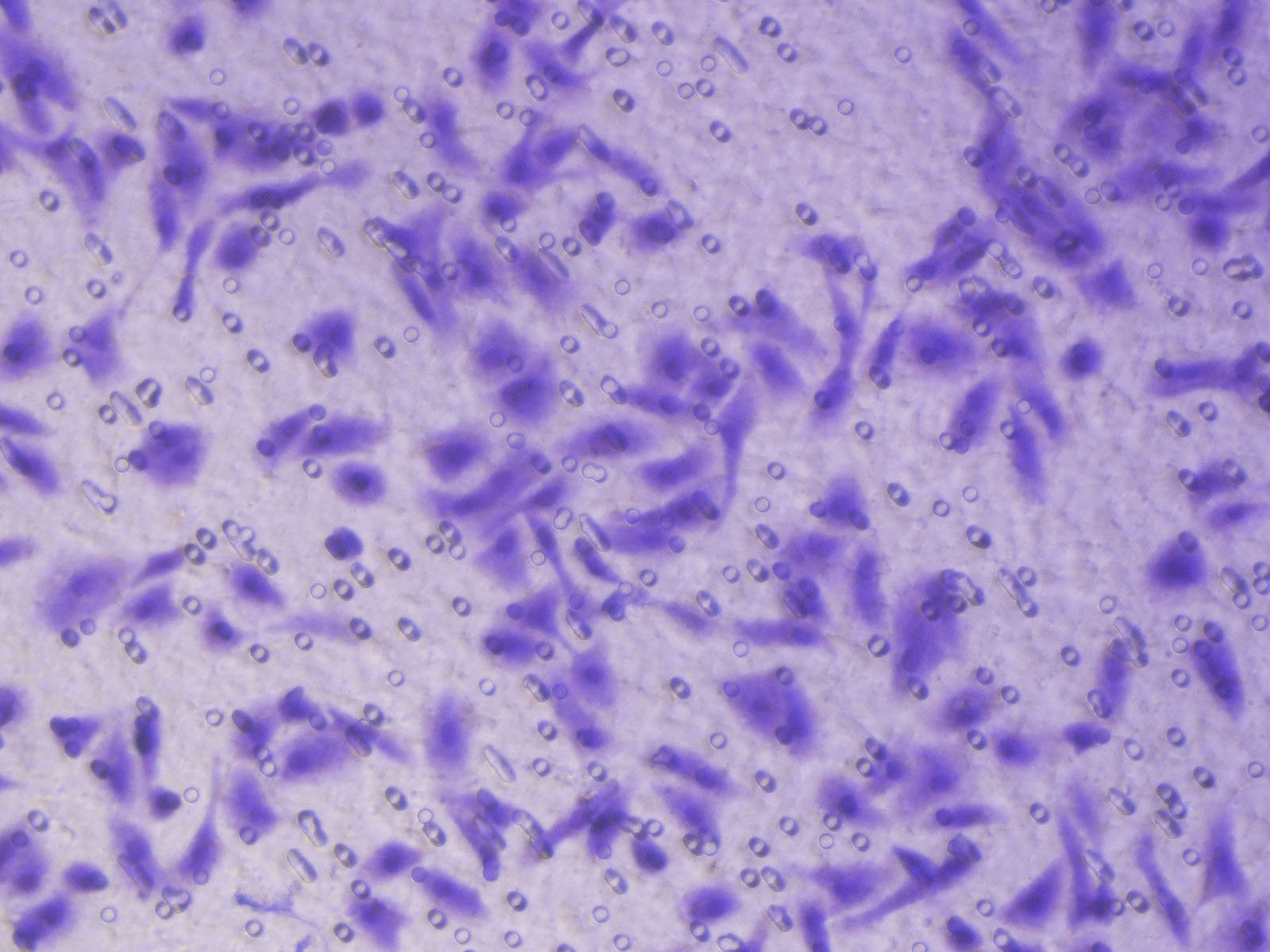

Supplement: Supplementary file 19 — Source Data [file 41467_2023_43282_MOESM19_ESM.zip › Source Data/Source data-Transwell raw images/Migration/C ES-2/Control-rep1.jpg]

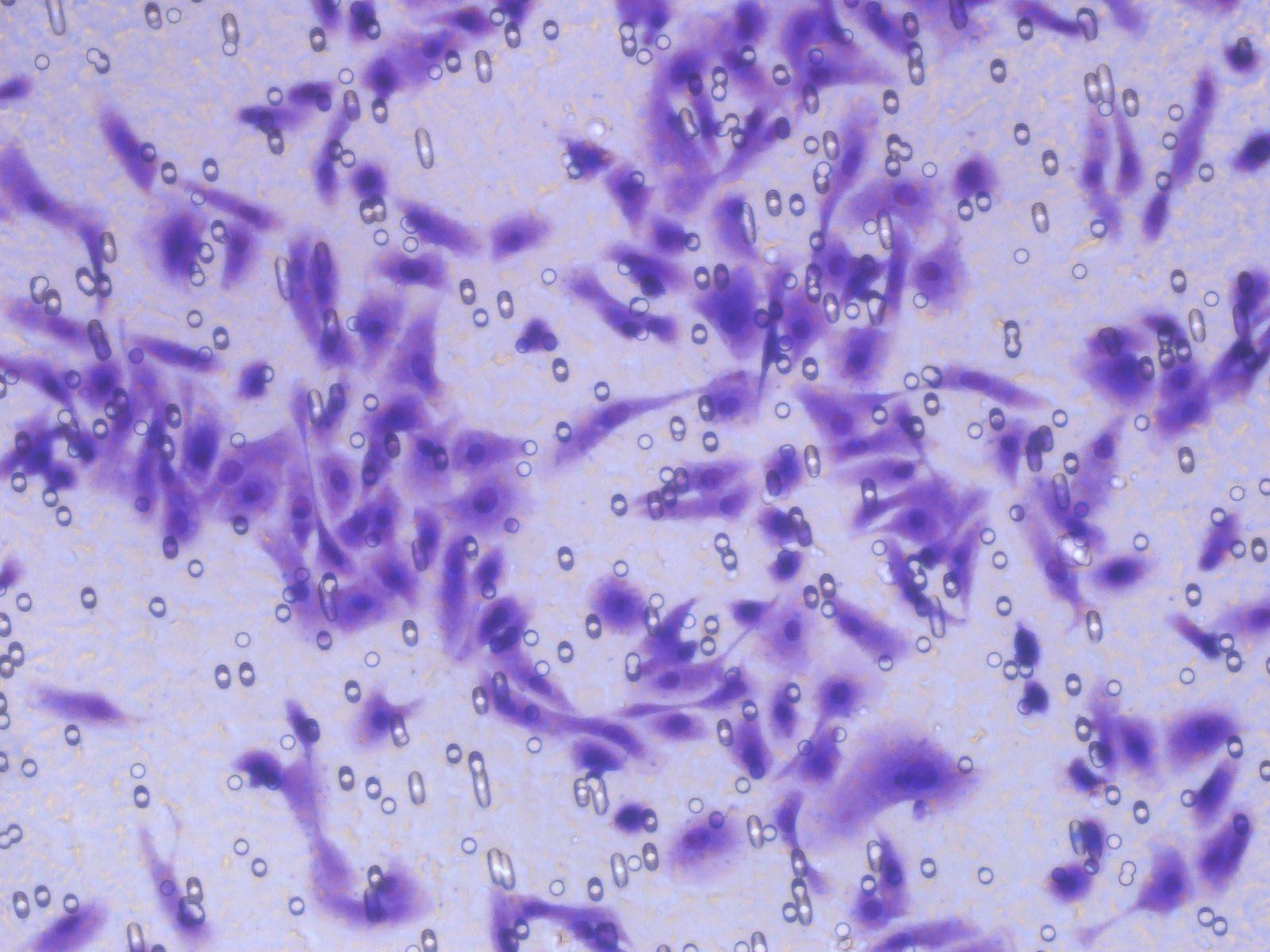

Supplement: Supplementary file 19 — Source Data [file 41467_2023_43282_MOESM19_ESM.zip › Source Data/Source data-Transwell raw images/Migration/C ES-2/Control-rep2.jpg]

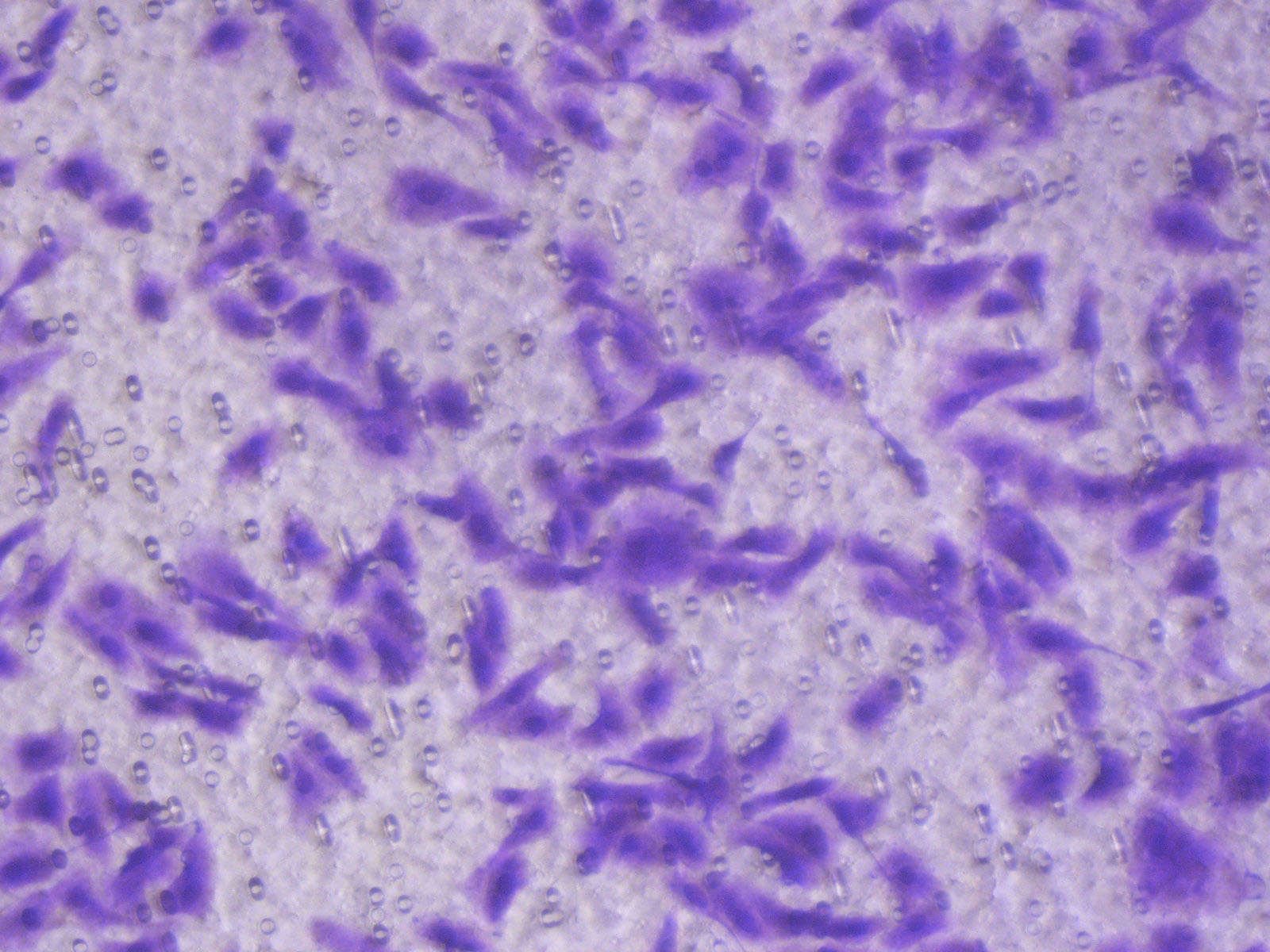

Supplement: Supplementary file 19 — Source Data [file 41467_2023_43282_MOESM19_ESM.zip › Source Data/Source data-Transwell raw images/Migration/C ES-2/Control-rep3.jpg]

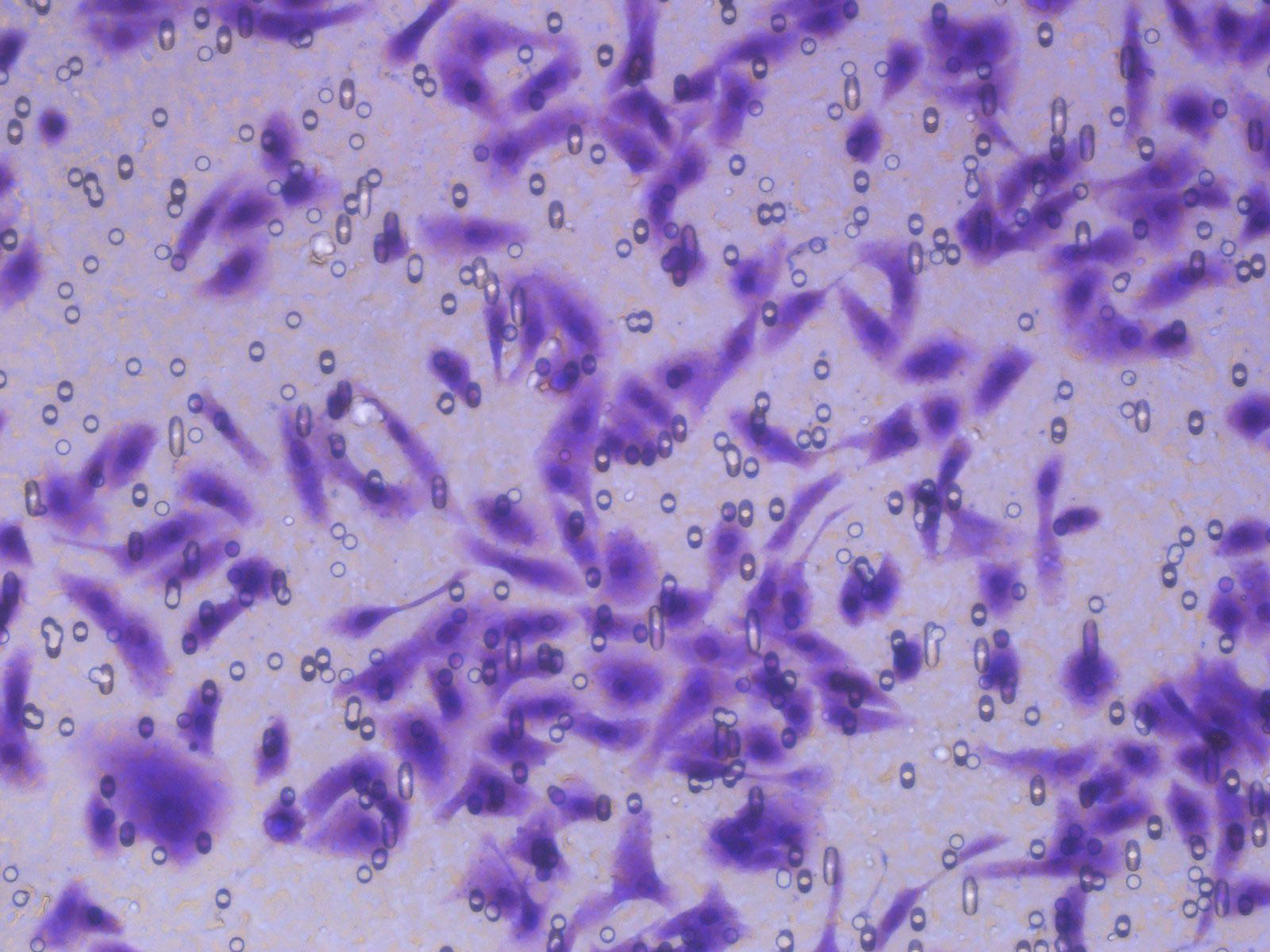

Supplement: Supplementary file 19 — Source Data [file 41467_2023_43282_MOESM19_ESM.zip › Source Data/Source data-Transwell raw images/Migration/C ES-2/Control-rep4.jpg]

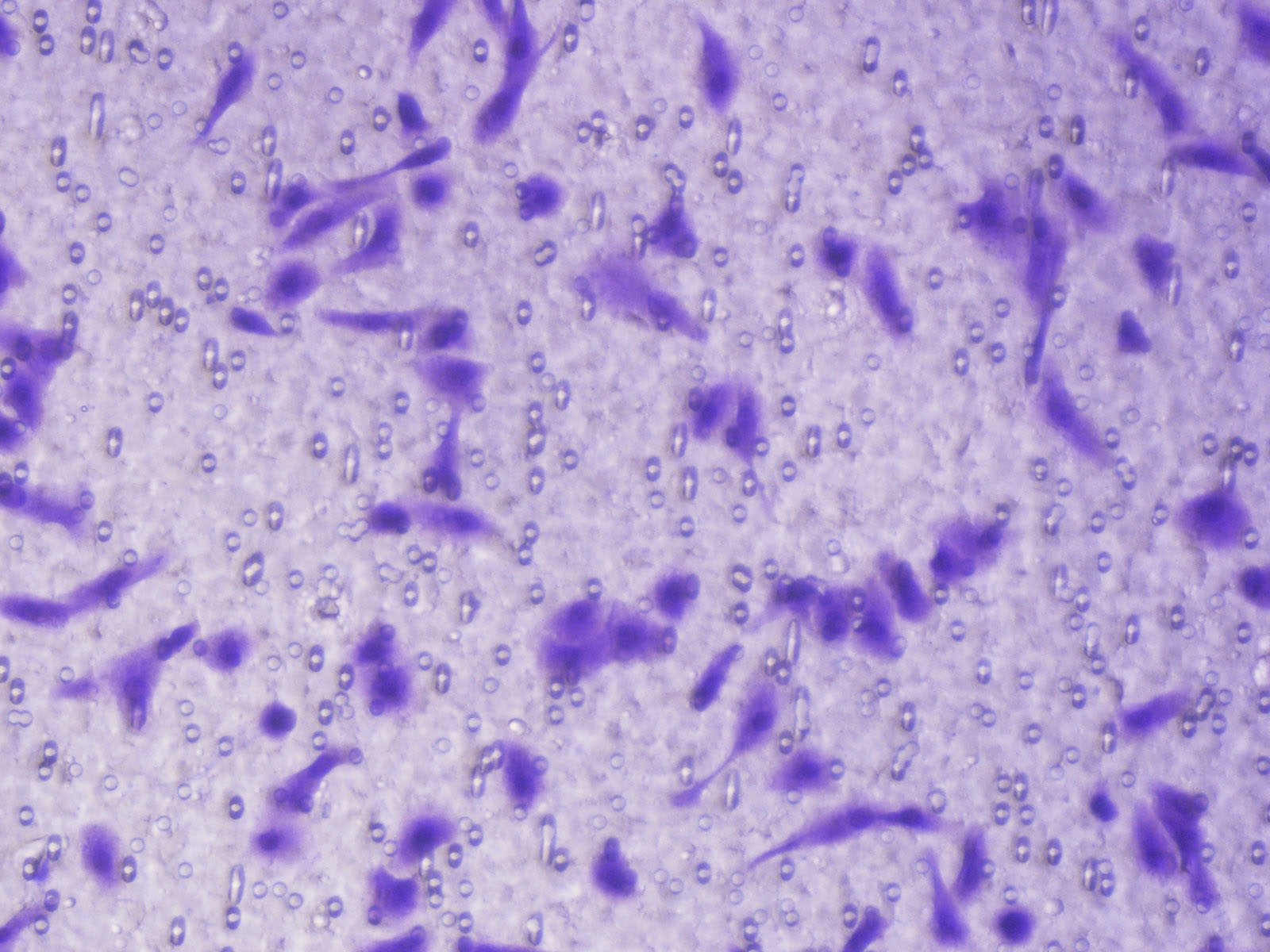

Supplement: Supplementary file 19 — Source Data [file 41467_2023_43282_MOESM19_ESM.zip › Source Data/Source data-Transwell raw images/Migration/C ES-2/shMPP7-rep1.jpg]

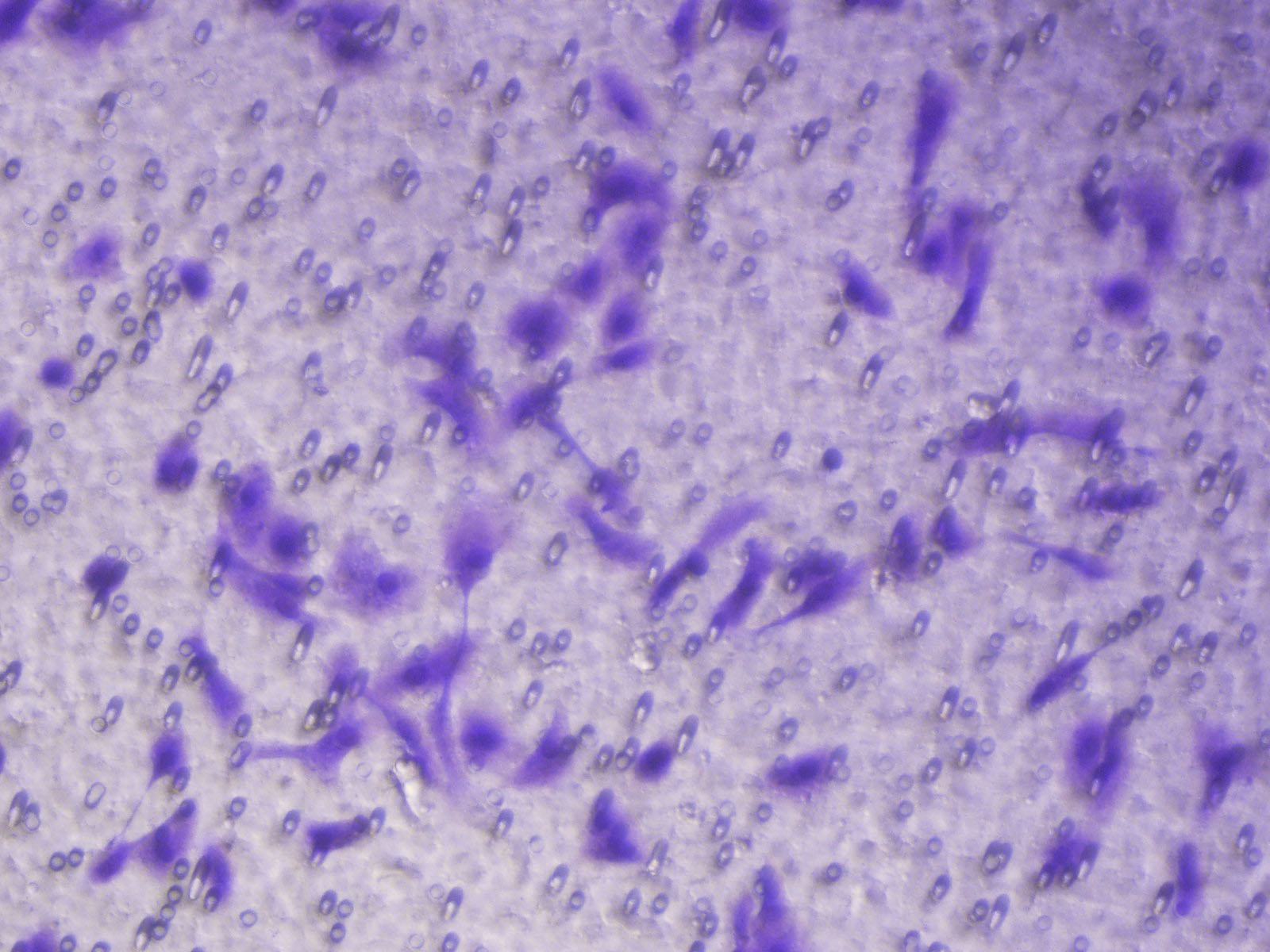

Supplement: Supplementary file 19 — Source Data [file 41467_2023_43282_MOESM19_ESM.zip › Source Data/Source data-Transwell raw images/Migration/C ES-2/shMPP7-rep2.jpg]

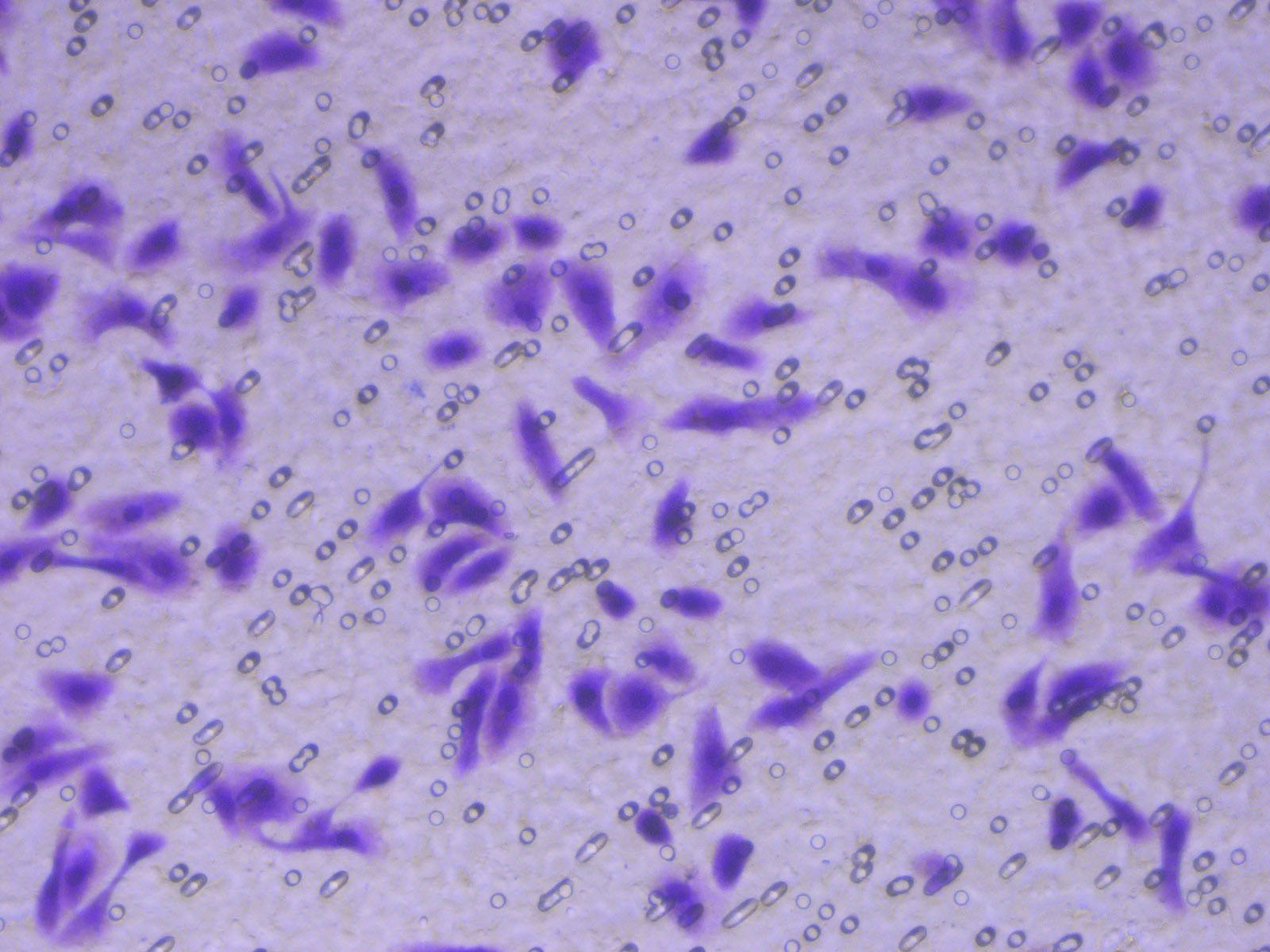

Supplement: Supplementary file 19 — Source Data [file 41467_2023_43282_MOESM19_ESM.zip › Source Data/Source data-Transwell raw images/Migration/C ES-2/shMPP7-rep3.jpg]

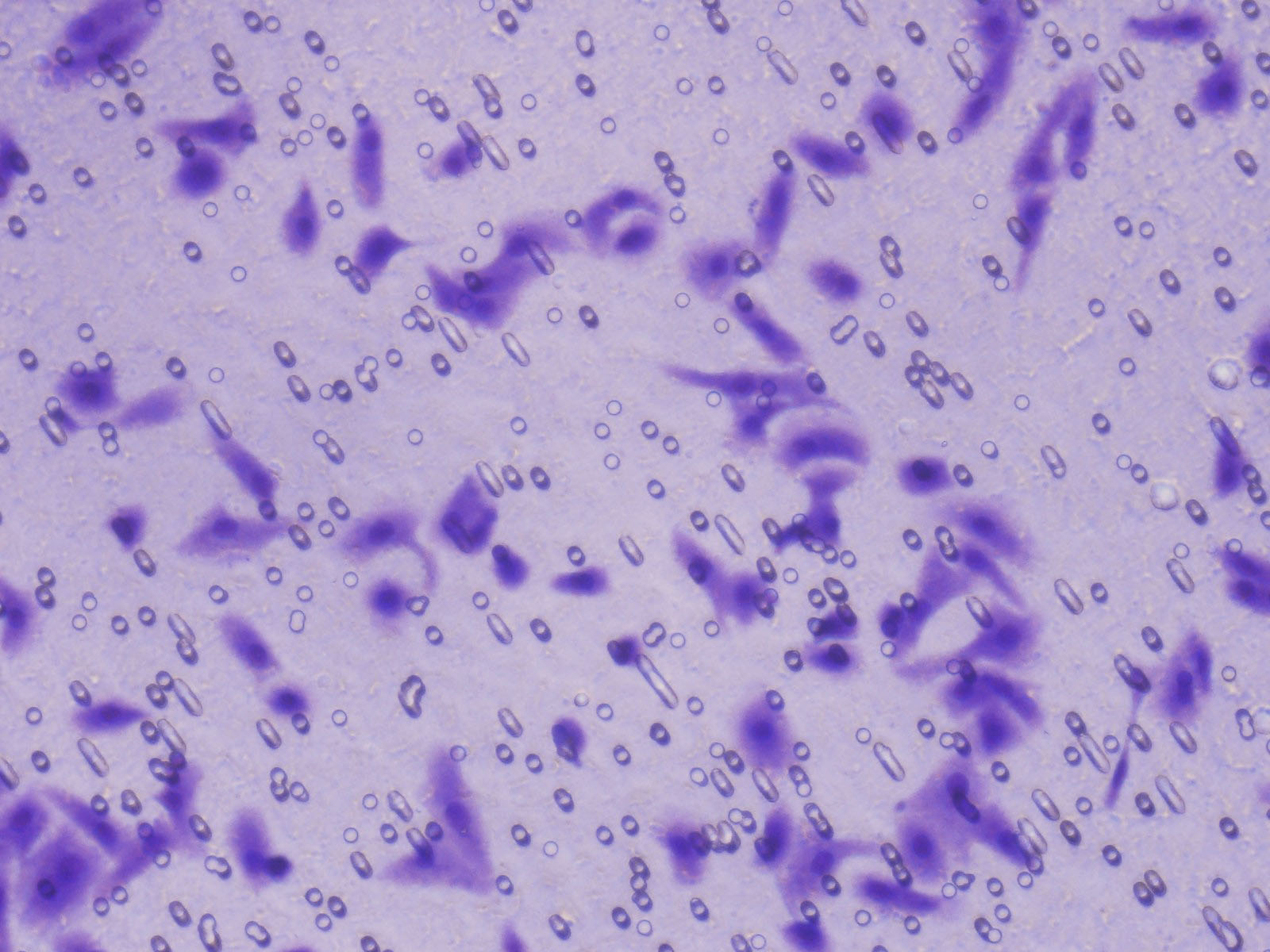

Supplement: Supplementary file 19 — Source Data [file 41467_2023_43282_MOESM19_ESM.zip › Source Data/Source data-Transwell raw images/Migration/C ES-2/shMPP7-rep4.jpg]

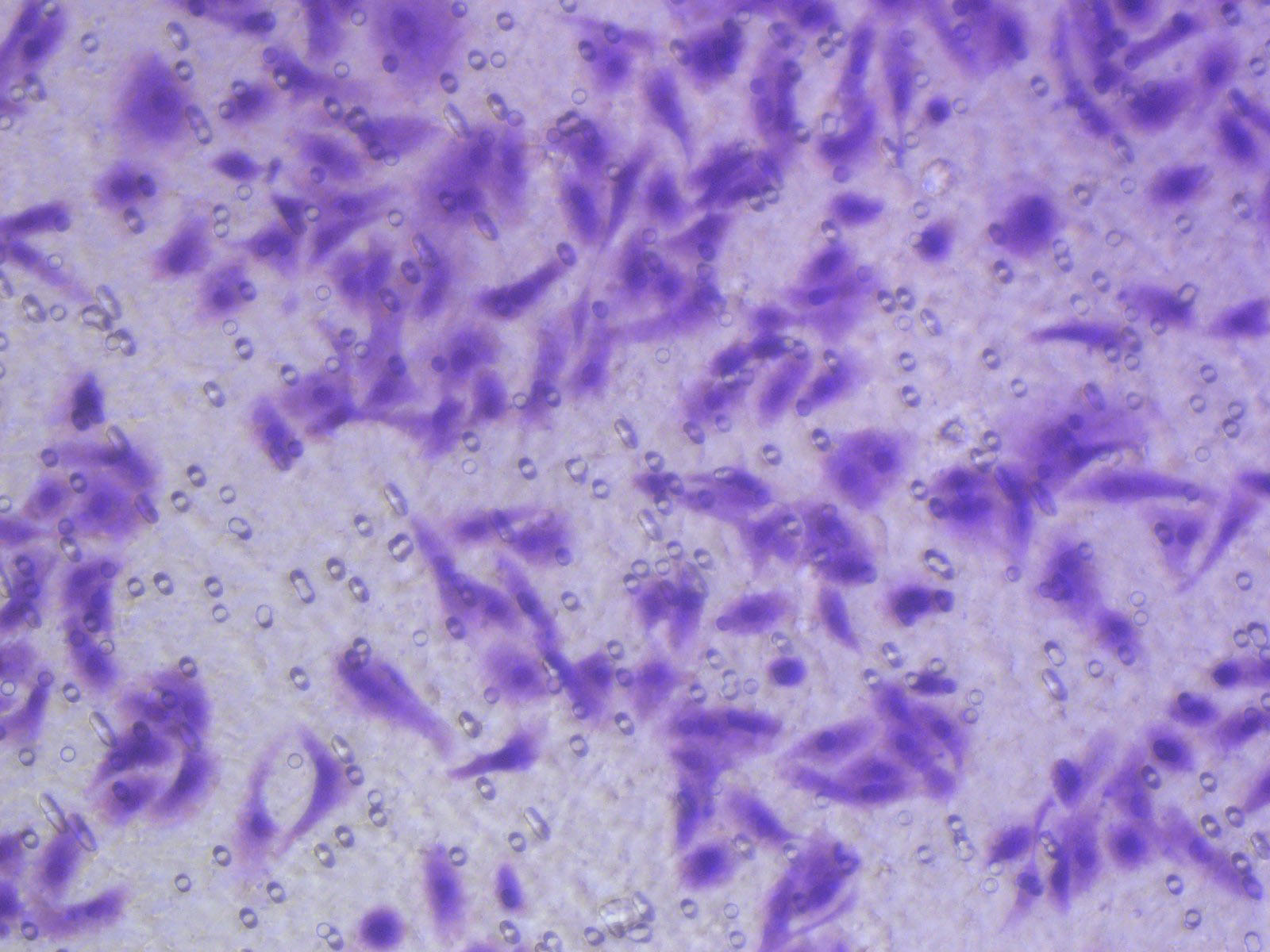

Supplement: Supplementary file 19 — Source Data [file 41467_2023_43282_MOESM19_ESM.zip › Source Data/Source data-Transwell raw images/Migration/C ES-2/shNC-rep1.jpg]

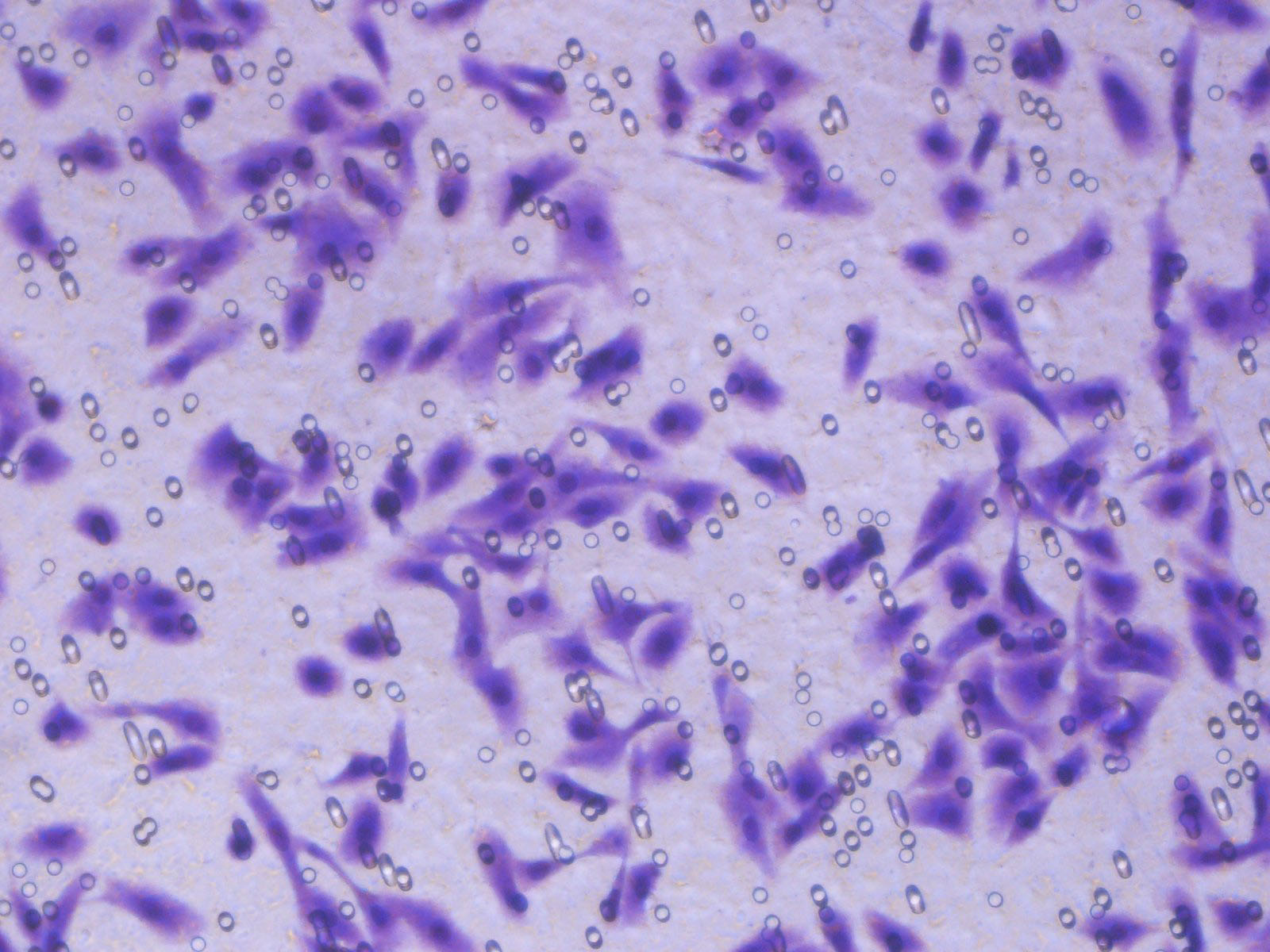

Supplement: Supplementary file 19 — Source Data [file 41467_2023_43282_MOESM19_ESM.zip › Source Data/Source data-Transwell raw images/Migration/C ES-2/shNC-rep2.jpg]

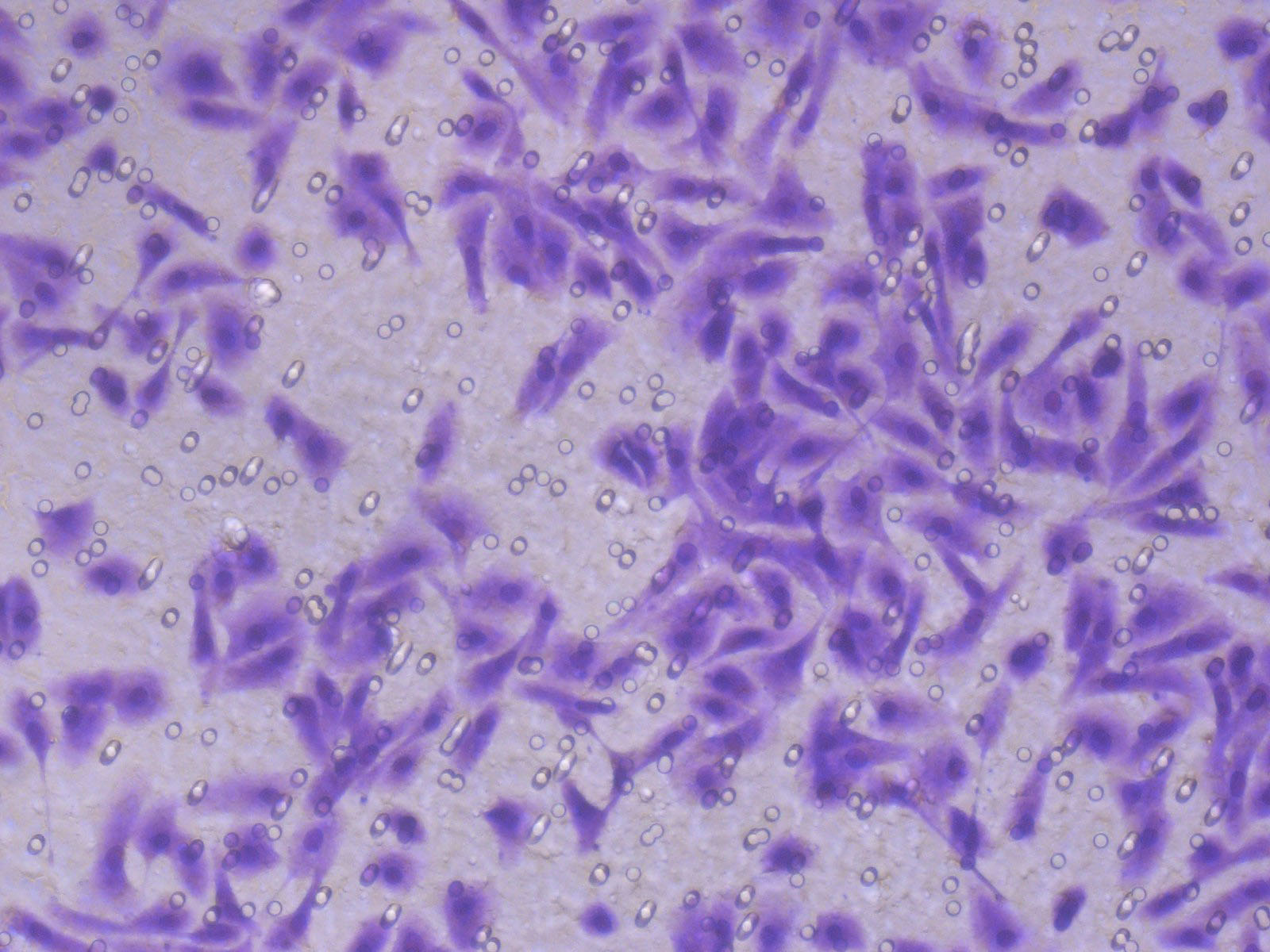

Supplement: Supplementary file 19 — Source Data [file 41467_2023_43282_MOESM19_ESM.zip › Source Data/Source data-Transwell raw images/Migration/C ES-2/shNC-rep3.jpg]

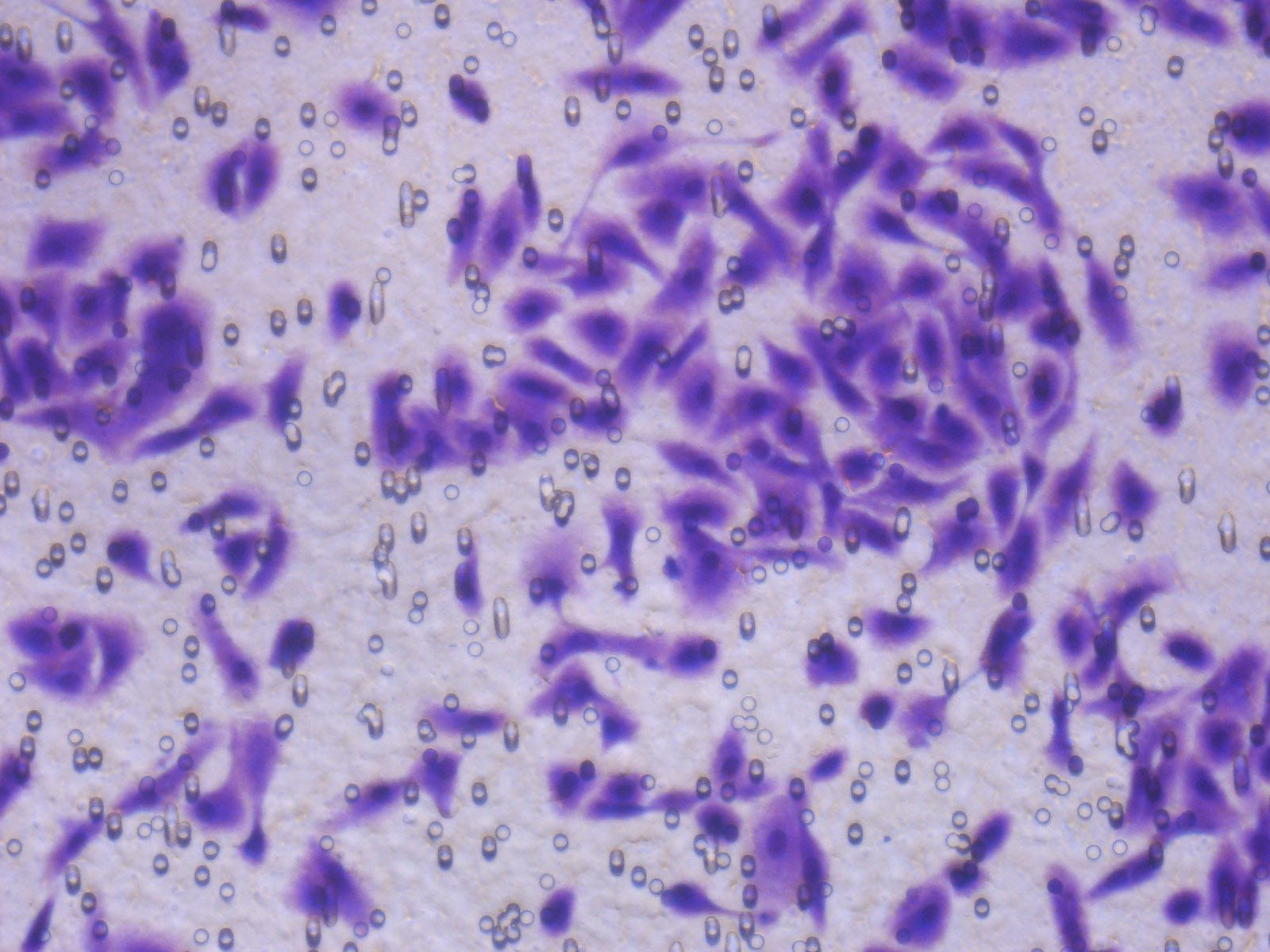

Supplement: Supplementary file 19 — Source Data [file 41467_2023_43282_MOESM19_ESM.zip › Source Data/Source data-Transwell raw images/Migration/C ES-2/shNC-rep4.jpg]
